# Supplementary material for: Structural and functional studies of rabbit SAMD9 reveal a distinct tRNase module that underlies the antiviral activity
Source: PLoS Pathog. 2025 Jul 31;21(7):e1013118. doi: 10.1371/journal.ppat.1013118 (PMC12331169; doi:10.1371/journal.ppat.1013118)
Supplement: S1 Fig — The tree was constructed using the NCBI BLAST Fast Minimum Evolution Tree Builder, with most branches collapsed to highlight only four lagomorph SAMD9 proteins. (B) Multiple sequence alignment of the tRNase domains from the same set of. SAMD9/9L proteins. Acidic (blue) and basic (red) residues are highlighted. Protein accession numbers are shown alongside each sequence. The numbering above the alignment indicates positions in human SAMD9. (C) Phylogenetic tree of the four lagomorph species, constructed using the TimeTree server. (PDF) [file ppat.1013118.s001.pdf]

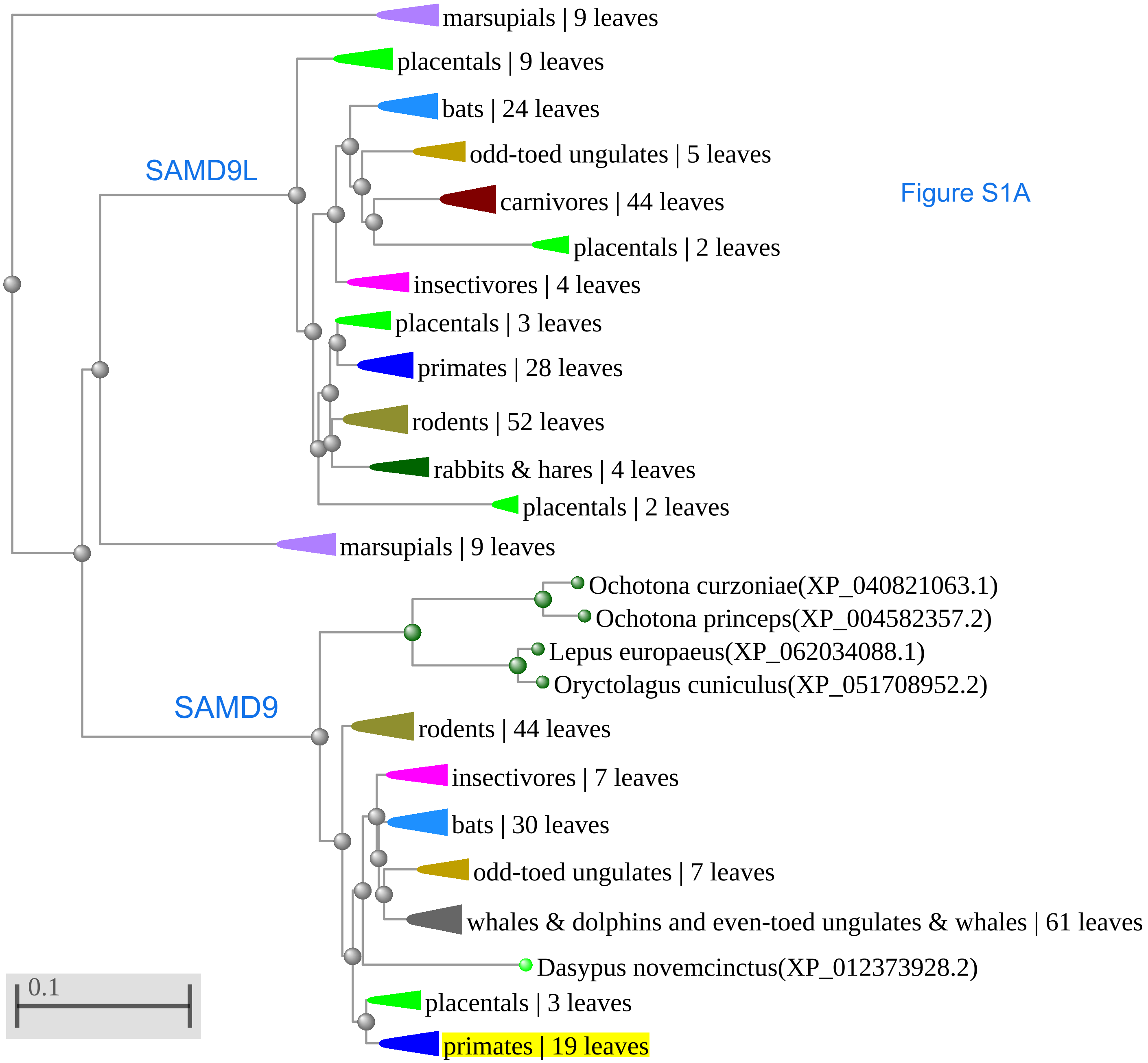
marsupials | 9 leaves
placentals | 9 leaves
bats | 24 leaves
odd-toed ungulates | 5 leaves
carnivores | 44 leaves
placentals | 2 leaves
insectivores | 4 leaves
placentals | 3 leaves
primates | 28 leaves
rodents | 52 leaves
rabbits & hares | 4 leaves
placentals | 2 leaves
marsupials | 9 leaves
SAMD9L
Figure S1A
Ochotona curzoniae(XP_040821063.1)
Ochotona princeps(XP_004582357.2)
Lepus europaeus(XP_062034088.1)
Oryctolagus cuniculus(XP_051708952.2)
SAMD9
rodents | 44 leaves
insectivores | 7 leaves
bats | 30 leaves
odd-toed ungulates | 7 leaves
whales & dolphins and even-toed ungulates & whales | 61 leaves
Dasypus novemcinctus(XP_012373928.2)
placentals | 3 leaves
primates | 19 leaves
0.1

Figure S1B

Reference sequence (1): ref|NP_001180236.1|
Identities normalised by aligned length.
Colored by: property

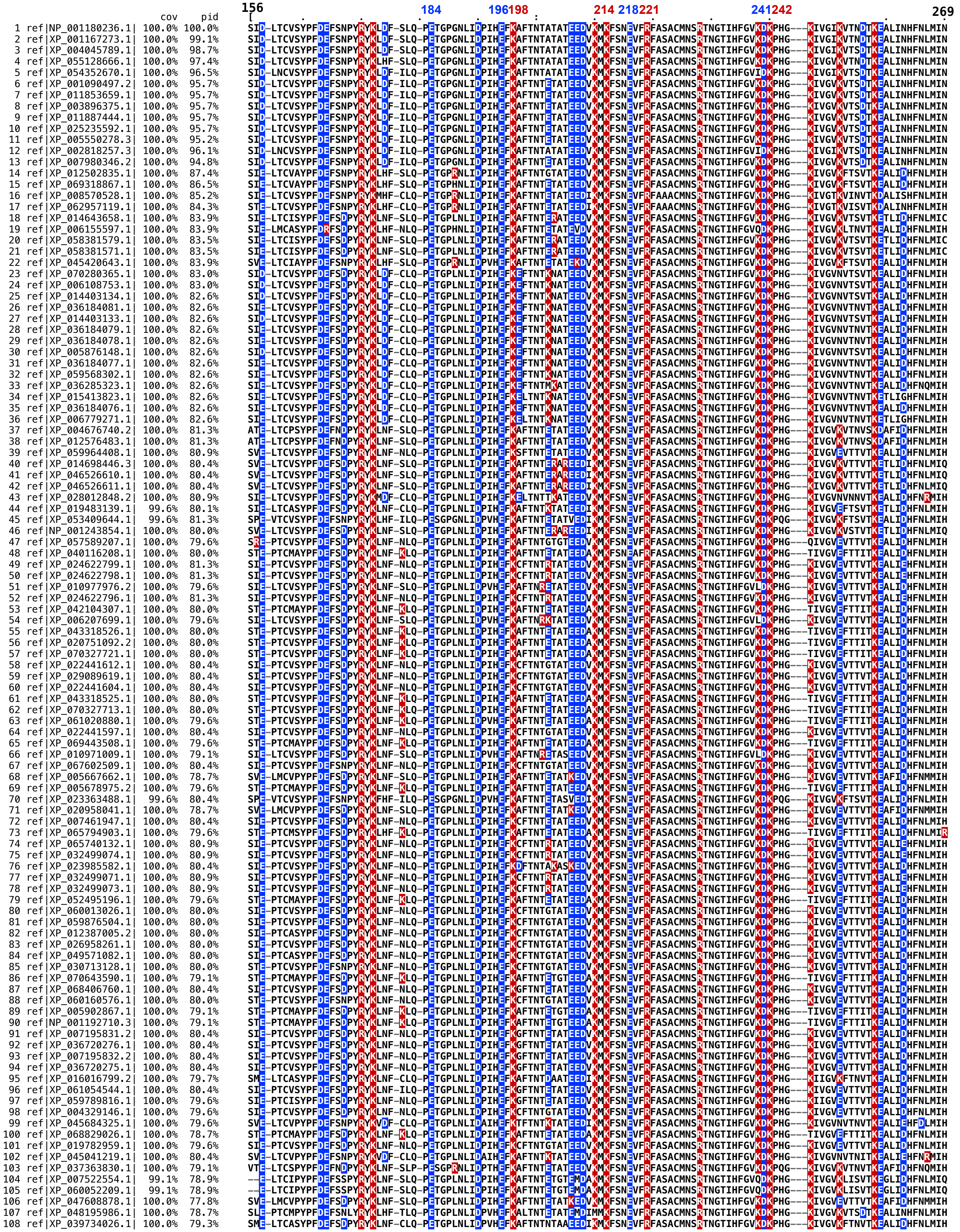

```
109 ref|XP_028381693.1| 100.0%  79.1%   SVE-LTCVPYPFDEFSDPYRYKVDF-CLQ-PETGPLNLIDAIHEFKTFTNTKTATEEDTKMKFSNEVFRFASACMNSRTNGTIHFGVKDKPHG---KIVGVNVTNVTKEALIEHFDLMIH
110 ref|XP_016074271.1| 100.0%  78.3%   SVE-LTCAAYPFDEFSDPYRYKMNF-CLQ-PETGPLNLIDPIHEFKDFTNTNKGTELDAKMKFSNEVFKFASACMNSRTNGTIHFGVIDKPHG---KIVGVSLTTVTKEALINHFNLMIH
111 ref|XP_036992384.2| 100.0%  79.1%   SIE-LTCVPYPFDEFSDPYRYKVDF-YLQ-PETGPLNLIDAIHEFKAFTNTKTDKEEDAKMKFSNEVFRFASACMNSRTNGTIHFGVIDKPHG---KIVGVNVTSVTKEALIQHFNLMIH
112 ref|XP_006911959.1| 100.0%  78.4%   SME-LTCASYPFDEFSDPYRYKLNF-CLQ-PETGPLNLIDPVHEFKAFTNTNTAAEEDIKMKFSNEVFRFASACMNSRTNGTIHFGVKDKPHG---KIVGVKFTNVTKEALIDHFNLMIH
113 ref|XP_011361926.1| 100.0%  78.4%   SME-LTCASYPFDEFSDPYRYKLNF-CLQ-PETGPLNLIDPVHEFKAFTNTNTAAEEDIKMKFSNEVFRFASACMNSRTNGTIHFGVKDKPHG---KIVGVKFTNVTKEALIDHFNLMIH
114 ref|XP_020016740.1| 100.0%  78.7%   SLE-PTCVPYPFDEFNDPYRYKLHF-HLQ-PETGPLNLIDPVHEFKALTNTETATEEDIKMKFSNEVFRFASACMNSRTNGTIHLGVEDKPHG---KIVGIKVTKVTKEDLINHFDLMIH
115 ref|XP_020016739.1| 100.0%  78.7%   SLE-PTCVPYPFDEFNDPYRYKLHF-HLQ-PETGPLNLIDPVHEFKALTNTETATEEDIKMKFSNEVFRFASACMNSRTNGTIHLGVEDKPHG---KIVGIKVTKVTKEDLINHFDLMIH
116 ref|XP_004862414.1|  98.7%  77.1%   ----PTCTPYPFNTFSDPYRYKLHF-ILQ-PETGPQNLIDPVHEFKALKNTETATEEDIKMKFTNETFRFASACMNSRTNGTIHFGVEDKPHG---KIVGIKVTSVTKEALIDHFNWMIH
117 ref|XP_012934202.1|  98.7%  77.1%   ----PTCTPYPFNTFSDPYRYKLHF-ILQ-PETGPQNLIDPVHEFKALKNTETATEEDIKMKFTNETFRFASACMNSRTNGTIHFGVEDKPHG---KIVGIKVTSVTKEALIDHFNWMIH
118 ref|XP_054420471.1|  99.6%  79.6%   STD-LTCVSYPFDEFSDPYRYKLDF-CLQ-PETGPLNLIDPIHEFKELTKTKTATEKDVKMKFSNEVFRFASACMNSRTNGTIHFGVKDKPHG---KIVGVT-TNVTKEALIDHFNQMIC
119 ref|XP_066210460.1| 100.0%  77.8%   STE-PTCAPYPFDKFNEPYCYKLNF-CLP-PETGPHNLIDPIHEYKELTNTEKATEEDVKMKFSNEVFRFASACMNSRTNGTIHLGVKDKPHG---KIVGVNVTNVTKEAFIDHFNLMIH
120 ref|XP_013013899.2|  98.7%  75.8%   ----PTCLPYPFNTFSNPYRYKLHF-ILQ-PETGPQNLIDPIHEFKALTDTATATEKDIKMKFSNETFRFASACMNSRTNGTIHFGVKDKPHG---KIVGMKVTSVTKEALIEHFNSMIP
121 ref|XP_066095023.1| 100.0%  77.4%   STE-PTCAPYPFDKFNEPYCYKLNF-CLP-PETGPRNLIDPIHEYKELTNTEKATEEDVKMKFSNEVFRFASACMNSRTNGTIHLGVKDKPHG---KIVGVNVTNVTKEAFIDHFNLMIH
122 ref|XP_004646297.1|  98.7%  74.9%   ----PTCTPYPFNTFSDPYRYKLHF-ILQ-PETGPQNLIDPAHEFKALTNTETGTEEDTKMKFSNETFRFASACMNSRTNGTIHFGVKDKPHG---EIVGVKVTSVTKEALINHFNCMIH
123 ref|XP_059113322.1| 100.0%  75.8%   SFPGPTCVAYPFNEFSNPYRYKLHF-ILQ-PETGPLNLIDPVHEFKAFTNTATATEEDIKMKFSNETFRFASACMNSRTNGTIHFGVKDKPQG---KIVGVELTTVTKDALIDHFNLMID
124 ref|XP_032944686.1|  98.7%  77.8%   STE-LTCVSYPFDEFSDPYRYKLNF-CLQ-PETGPLNLIDPVHEFKAFTDTKT--EEDAKMKFSNEVFRFASACMNSRTNGTIHLGVKDKPHG---KIVGVELTHVTKEALINHFNQMIH
125 ref|XP_010838083.1|  99.1%  77.0%   STE-PTCMAYPFDEFSDPYRYKLNF-KLQ-PETGPLNLIDPIHEFKDFTNPETAMQ--AKMKFSNEVFRFASACMNSRTNGTIHFGVKDKPHG---IIVGVEFTTITKEALIDHFNLMIH
126 ref|XP_052575709.1| 100.0%  75.3%   SSPGPTCVAYPFNEFSNPYRYKLHF-ILQ-PETGPLNLIDPVHEFKAFTNTATATEKDIKMKFSNETFRFASACMNSRTNGTIHFGVKDKPQG---KIVGVELTTVTKDALIDHFNLMID
127 ref|XP_019585265.1|  98.7%  77.4%   STE-LTCVSYPFDEFSDPYRYKLNF-CLQ-PETGPLNLIDPIHEFKAYTDTKT--EEDAKMKFSNEVFRFASACMNSRTNGTIHLGVKDKPHG---KIVGVELTHVTKEALIDHFNKMIY
128 ref|XP_036037353.1| 100.0%  74.9%   SSPGPTCVPYPFNEFSNPYRYKLHF-TLQ-PETGPLNLIDPVHEFKAFTNTETATEEDIKMKFSNEAFRFASACMNSRTNGTIHFGVKDKPQG---KIVGVELTTVTKDTLINHFSLMIG
129 ref|XP_055445695.1|  99.1%  75.7%   STE-PTCMAYPFDEFSDPYRYKLNF-KLQ-PETGPLNLIDPIHEFKDFTNTETAMQ--AKMKFSNEVFRFASACMNSRTNGTIHLGVKDKPHG---KIVGVEFTTITKEALIDHFNLMIH
130 ref|XP_061269427.1|  99.1%  76.1%   STE-PTCMAYPFDEFSDPYRYKLNF-KLQ-PETGPLNLIDPIHEFKDFTNPETAMQ--AKMKFSNEVFRFASACMNSRTNGTIHFGVKDKPHG---IIVGVEFTTITKEALIDHFNLMIH
131 ref|XP_012881537.1| 100.0%  77.0%   SPE-PTCVPYPFDEFSDPYRYKSHF-ILQ-PETGPLNLIDPVHEFKALTDTERATETDIKMKFSNETFRFASACMNSRTNGTIHFGVKDKPHG---KIIGVKVTSDTKEALINHFNLMIH
132 ref|XP_051033135.1| 100.0%  73.6%   SPPGPTCIAYPFNEFSNPYRYKLHF-ILQ-PETGPLNLIDPVHEFKAFTNTEKATEEDIKMKFSNEIFRFASACMNSRTNGTIHFGVKDKPQG---KIVGVELTTVTKDTLINHFNLMID
133 ref|XP_069852599.1| 100.0%  76.5%   SPE-PTCVPYPFDEFSDPYRYKSHF-ILQ-PETGPLNLIDPVHEFKALTDTERATETDIKMKFSNETFRFASACMNSRTNGTIHFGVKDKPHG---EIIGVKVTSDTKEALINHFNLMIH
134 ref|XP_006078386.4|  99.1%  75.2%   STE-PTCMAYPFDEFSDPYRYKLNF-KLQ-PETGPLNLIDPIHEFKDFTNTETAMQ--AKMKFSNEVFRFASACMNSRTNGTIHLGVKDKPHG---KIVGVEFTTITKEALVDHFNLMIH
135 ref|XP_061269426.1|  99.1%  76.1%   STE-PTCMAYPFDEFSDPYRYKLNF-KLQ-PETGPLNLIDPIHEFKDFTNPETAMQ--AKMKFSNEVFRFASACMNSRTNGTIHFGVKDKPHG---IIVGVEFTTITKEALIDHFNLMIH
136 ref|XP_006078384.4|  99.1%  75.2%   STE-PTCMAYPFDEFSDPYRYKLNF-KLQ-PETGPLNLIDPIHEFKDFTNTETAMQ--AKMKFSNEVFRFASACMNSRTNGTIHLGVKDKPHG---KIVGVEFTTITKEALVDHFNLMIH
137 ref|XP_006991270.1| 100.0%  74.5%   SSPGPTCVAYPFNEFSNPYRYKLHF-ILQ-PETGPLNLIDPVHEFKAFTNTATATEEDIKMKFSNETFRFASACMNSRTNGTIHFGVEDKPQG---KIVGVELTTVTKDALIDHFNLMIE
138 ref|XP_028725617.1| 100.0%  74.5%   SSPGPTCVAYPFNEFSNPYRYKLHF-ILQ-PETGPLNLIDPVHEFKAFTNTATATEEDIKMKFSNETFRFASACMNSRTNGTIHFGVEDKPQG---KIVGVELTTVTKDALIDHFNLMIE
139 ref|XP_008820770.1| 100.0%  76.1%   SLE-PTCIPYPFDEFSSPYRYKLHF-ILQ-PETGPLNLIDPVHEFKALTNTETAKEEDIKMKFSNEIFRFASACMNSRTNGTIHFGVKDKPHG---KIVGMHVTTVTKEALIDHFDLMIH
140 ref|XP_044802364.2|  99.1%  75.2%   STE-PTCMAYPFDEFSDPYRYKLNF-KLQ-PETGPLNLIDPIHEFKDFTNTETAMQ--AKMKFSNEVFRFASACMNSRTNGTIHLGVKDKPHG---KIVGVEFTTITKEALVDHFNLMIH
141 ref|XP_008062458.2| 100.0%  73.9%   FME-ITCAPYPFDESNNQYCYKLGF-SLP-PETGPGNLIDPVREFKSFTNTETATEEDIKMKFSNEIFRFASACMNSRTNGTIHLGVIDKPHG---KIVGVTLTRVTKEALIDHFNQMTH
142 ref|XP_041531686.1| 100.0%  73.6%   SPPGPTCIAYPFNEFSNPYRYKLHF-ILQ-PETGPLNLIDPVHEFKAFTNTEAATEVDIKMKFSNETFRFASACMNSRTNGTIHFGVKDKPQG---KIVGVELTTVTKDALIDHFNLMID
143 ref|XP_040584508.1| 100.0%  73.6%   SPPGPTCTAYPFNEFSNPYRYKLHF-ILQ-PETGPLNLIDPVHEFKDFTNTEKATEEDIKMKFSNEIFRFASACMNSRTNGTIYFGVKDKPQG---KIVGVELTTVTKDTLINHFNLMID
144 ref|XP_021045894.1| 100.0%  73.2%   SFPGPTCIAYPFNEFSSPYRYKLNF-ILQ-PETGPLNLIDPIHEFKALTNTESATEKDIKMKFSNEVFRFASACMNSRTNGTIHFGVEDKPHG---KIVGVELTTVTKDALIDHFNLMIA
145 ref|XP_048292645.1| 100.0%  74.0%   SPPGPTCVAYPFNEFSNPYRYKLHF-ILQ-PETGPLNLIDPVHEFKAFTNTETATEVDIKMKFSNETFRFASACMNSRTNGTIHFGVKDKPQG---KIVGVELTMVTKDALIDHFNLMID
146 ref|XP_021045323.1| 100.0%  73.6%   SFPGPTCIAYPFNEFSSPYRYKLNF-ILQ-PETGPLNLIDPIHEFKAFTNTEKATEEDIKIKFSNEVFRFASACMNSRTNGTIHFGVEDKPHG---KIVGVELTTVTKDALIDHFNLMIA
147 ref|XP_042540450.1| 100.0%  76.1%   SPE-PTCVPYPFDEFSHPYRYKSHF-ILQ-PETGPLNLIDPVHEFKALTDTERATEIDIKMKFSNETFRFASACMNSRTNGTIHFGVKDKPHG---EIIGLKVTSDTKEALINHFNLMIH
148 ref|XP_047418909.1| 100.0%  75.7%   SLE-PTCIPYPFDKFSDPYRYKLHF-ILQ-PETGSLNLIDPAHEFKALTDTKRATEEDIKMKFSNEVFRFAAACMNSRTNGTIHFGVEDKPHG---RIVGVQIAKVTKEALIEHFHKMIH
149 ref|XP_034375203.1| 100.0%  73.6%   SSPGPTCIAYPFNEFSNPYRYKLHF-ILQ-PETGPLNLIDPIHEFKALTNTETATKEDIKMKFSNEVFRFASACMNSRTNGTIHFGVKDKPQG---EIVGVELTTVTKDALIDHFNLTIA
150 ref|XP_038176268.1| 100.0%  73.6%   SPSGPTCVAYPFNEFSNPYRYKLHF-ILQ-PETGPLNLIDPVHEFKAFTNTETATEVDIKMKFSNETFRFASACMNSRTNGTIHFGVKDKPQG---KIVGVELTKVTKDALIDHFNLMID
151 ref|XP_038176270.1| 100.0%  73.6%   SPSGPTCVAYPFNEFSNPYRYKLHF-ILQ-PETGPLNLIDPVHEFKAFTNTETATEVDIKMKFSNETFRFASACMNSRTNGTIHFGVKDKPQG---KIVGVELTKVTKDALIDHFNLMID
152 ref|XP_028634992.1| 100.0%  73.6%   SSPGPTCIAYPFNEFSNPYRYKLHF-ILQ-PETGPLNLIDPIHEFKAFTNTETATEEDIKMKFSNEVFRFASACMNSRTNGTIHFGVKDKPQG---EIVGVELTTVSKDALIDHFNLTIA
153 ref|XP_032762876.1| 100.0%  72.7%   ASPGPTCIAYPFNEFSNPYRYKLHF-ILQ-PETGPLNLIDPVHEFKALTNTETATEEDIKMKFSNEVFRFASACMNSRTNGTIHFGVQDKPQG---RIVGMELTTVTKDALIDHFNLMIV
154 ref|XP_057634583.1| 100.0%  73.2%   SPPGPTCVAYPFNEFSHSYRYKLHF-ILQ-PETGPLNLIDPVHEFKAFTNTEAATEVDIKMKFSNETFRFASACMNSRTNGTIHFGVKDKPQG---KIVGVELTTVTKDAIIDHFNLMID
155 ref|NP_001386282.1| 100.0%  72.3%   ASPGPTCIAYPFNEFSNPYRYKLHF-ILQ-PETGPLNLIDPVHEFKALTNTETATEEDIKMKFSNEVFRFASACMNSRTNGTIHFGVQDKPQG---RIVGMELTTVTKDALIDHFNLMIV
156 ref|XP_015362209.1| 100.0%  76.1%   CLE-PTCIPYPFDESSDPYRYKLHF-ILQ-PETGSRNLIDPAREFKAFTNTKTATEEDIKMKFSNEVFRFASACMNSRTNGTIHLGVEDKPHG---KIVGVQITKVTKEALIEHFHKMIH
157 ref|XP_019059962.1|  98.7%  73.1%   ----PTCTPYPFNTFSDPYRYKLHF-ILQ-PETGPHNLIDPVHEFKALKNIETATEGDIKMKFSNETFRFASACMNSRTNGTIHFGVEDKPHG---KIVGVKFTSVTKEALIDHFNWMIH
158 ref|XP_046311157.1| 100.0%  76.1%   CLE-PTCIPYPFDESSDPYRYKLHF-ILQ-PETGSRNLIDPAREFKAFTNTKTATEEDIKMKFSNEVFRFASACMNSRTNGTIHLGVEDKPHG---KIVGVQITKVTKEALIEHFHKMIH
159 ref|XP_027775683.2| 100.0%  76.1%   CLE-PTCIPYPFDESSDPYRYKLHF-ILQ-PETGSRNLIDPAREFKAFTNTKTATEEDIKMKFSNEVFRFASACMNSRTNGTIHLGVEDKPHG---KIVGVQITKVTKEALIEHFHKMIH
160 ref|XP_026247365.1| 100.0%  75.7%   CLE-PTCIPYPFDESSDPYRYKLHF-ILQ-PETGSRNLIDPAREFKAFTNTKTATEEDIKMKFSNEVFRFASACMNSRTNGTIHLGVEDKPHG---KIVGVQITKVTKEALIEHFHKMIH
161 ref|XP_005340593.1| 100.0%  76.1%   CLE-PTCIPYPFDESSDPYRYKLHF-ILQ-PETGSRNLIDPAREFKALTNTKTATEEDIKMKFSNEVFRFASACMNSRTNGTIHLGVEDKPHG---KIVGVQITKVTKEALIEHFHKMIH
162 ref|XP_007652776.1| 100.0%  71.9%   SPPGPTCIAYPFNEFSNPYRYKLHF-ILQ-PETGPLNLIDPVHEFKAFTNTEKASEEDIKMKFSNEIFRFASACMNSRTNGTIHFGVKDKPQG---RIVGVDLSTVTKDTLINHFNLMID
163 ref|XP_055468986.1| 100.0%  71.9%   SSPGPTCIAYPFNEFSNPYRYKLHF-ILQ-PETGPLNLIDPVHEFKAFTNTEKATEEDIKMKFSNETFRFASACMNSRTNGTIHFGVKDKPQG---KIVGVELNQVTKDALINHFILMID
164 ref|XP_021508904.1| 100.0%  71.0%   SSPGPTCIAYPFNEFNNPYRYKLHF-TLQ-PETGPLNLIDPVHEFKAFTNTEKATEEDIKMKFSNETFRFASACMNSRTNGTIHFGVKDKPQG---KIVGVELNKVTKDALINHFILMID
165 ref|XP_012791283.2| 100.0%  70.9%   STE-QTCIAYPFDEFSDPYRYKENF-VLQ-PETGPLNLIDPIHEFKAFKNIEKGTKEDIKIKFSNEVFKFASACMNSRTNGTIHFGVEDKPHG---KIIGVRFIAITKEVLIDHFNQIIH
166 ref|XP_005388082.1|  98.7%  72.2%   ----PTCTPYPFDTFGDTYRYKLHF-ILQ-PETGPQNLIDPVHEFKALTNTETATEQDTKMKFSNETFRFASACMNSRTNGTIHFGVKDKPHG---KIVGVKFISVTKEALVDHFNCMIQ
167 ref|XP_052029157.1| 100.0%  73.6%   SSLGPTCIAYPFDEFSNPYRYKLHF-ILQ-PETGPLNLIDPVHEFKEFTNTRTATEQDIKMKFSNEVFRFASACMNSRTNGTIHFGVKDKPQG---EIVGVALTTVTKDDLIDHFNLMIA
168 ref|XP_055974093.1| 100.0%  70.0%   STE-VTCIAYPFDEFNDPYRYKENF-ILQ-PETGPLNLIDPIHEFKAFKNIEKGTEEDIKMKFSNEVFRFASACMNSRTNGTIHFGVEDKPHG---KIIGVRFTAITKEALIDHFNQIID
169 ref|XP_040821063.1|  99.1%  70.9%   PIE-PSCRAHPFNRFDELWRYKSNF-ILQ-PETGPQNLIDPIHEFKALTNTEKATEEDVKMKFSNEVFEFASACMNSRTNGTIHFGVKDKPHG---QIVGVKLANITKNALIDHFDKMID
170 ref|XP_045016549.1| 100.0%  68.7%   SPE-PSCFSYPFDEFHDSFRYKLHF-ILQ-PETGPLNLIDPVHEFKLLTNTETATEEDIMMKFSNETFKFASACMNSRTNGTIHFGVQDKPHG---KIVGIKVTTETKHSLINHFHKMIP
171 ref|XP_051007939.1| 100.0%  69.3%   SSPGPTCTPYPFNEFSNPYRYKLHF-ILQ-PETGPLNLIDPVHEFKALTNTDTATEEDIKMKFSNETFRFASACMNTRTNGTIHFGVQDKPQG---KIVGVELIRVTKDALIDHFNVMIS
172 ref|XP_004582357.2|  99.1%  69.1%   PIE-PSCRTHPFNRFDELWRYKSNF-ILQ-PETGPHNLIDPIHEFKALTNTEKATEEDVKMKFSNEVFEFASACMNSRTNGTIHFGVKDKPHG---QIVGVKLANITKNALIDHFDRMIG
173 ref|XP_062034088.1|  99.6%  68.8%   AKE-PSCRSHPFNKFDEQWHYKLHF-ILQ-PETGPHNLIDPIHEYKAFTNAEKTTEEDGKMKFSNEVFEFASACMNSRTNGTIHFGVKDKPHG---QIVGVKLASITKNALIDHFDKMIP
174 ref|XP_051708952.2|  99.6%  68.0%   AKE-PSCRSHPFNKFDEQWRYKLHF-ILQ-PETGPHNLIDPIHEYKAFTNAEKNTEEDGKMKFSNEVFEFASACMNSRTNGTIHFGVKDKPQG---QIVGVKLASITKNALIDHFDEMIP
175 ref|XP_012373928.2|  98.7%  73.5%   VVE-PTCPSHPFDEFNDLYNYKLHF-ILQ-PETGPLNLIDPIHEFKAFTNTEKAKEEDIKMKFSNEVFRFASACMNSRTNGTIYFGVMDTPHG---KIVG---TKPNKAALISHFNLMIE
176 ref|XP_015331500.1|  78.7%  79.0%   CLE-PTCIPYPFDESSDPYRYKLHF-ILQ-PETGSRNLIDPAREFKAFTNTKTATEEDIKMKFSNEVFRFASACMNSRTNGTIHLGVEDKPHG---KIVGVQITKVTKEALIEHFHKMIH
177 ref|XP_005340592.1|  98.7%  64.4%   QTEQLTCMSYPFDHFHDSHRYIEHY-ILQ-PETGPLNLIDPIHEFKALTNTDTATEEDIKMKFSNEVFRFASACMNSRTNGTIHFGVKDKPHG---EITGVKVTS--KDAFINHFNLMIK
178 ref|XP_015362203.1|  98.7%  64.4%   QTEQLTCMSYPFDHFHDSHRYIEHY-ILQ-PETGPLNLIDPIHEFKALTNTDTATEEDIKMKFSNEVFRFASACMNSRTNGTIHFGVKDKPHG---EIIGVKVTN--KDAFINHFNLMIK
179 ref|XP_046311151.1|  98.7%  64.4%   QTEQLTCMSYPFDHFHDSHRYIEHY-ILQ-PETGPLNLIDPIHEFKALTNTDTATEEDIKMKFSNEVFRFASACMNSRTNGTIHFGVKDKPHG---EIIGVKVTN--KDAFINHFNLMIK
180 ref|XP_050641313.1|  98.7%  64.0%   KPEQLTCMPYPFDQFHDSHRYIEHY-TLQ-PETGALNLIDPIHEFKALTNTETATEADIKMKFSNEVFRFASACMNSRTNGTIHFGVKDKPHG---EIVGVKITS--KDAFIDHFNLMIK
181 ref|XP_005550273.1|  98.7%  64.4%   KPEQLTCMPYPFDQFHDSHRYIEHY-TLQ-PETGALNLIDPIHEFKALTNTETATEADIKMKFSNEVFRFASACMNSRTNGTIHFGVKDKPHG---EIVGVKITS--KDAFIDHFNLMIK
182 ref|XP_014989728.2|  98.7%  64.4%   KPEQLTCMPYPFDQFHDSHRYIEHY-TLQ-PETGALNLIDPIHEFKALTNTETATEADIKMKFSNEVFRFASACMNSRTNGTIHFGVKDKPHG---EIVGVKITS--KDAFIDHFNLMIK
183 ref|XP_062956902.1|  98.7%  64.8%   KTELLTCMPYPFDQFHDSKRYIENY-ILQ-PETGPLNLIDPIHEFKALTNTETATEEDIKMKFSNEVFRFASACMNSRTNGTIHFGVKDEPHG---AIVGVKVTS--KDAFIDHFNLMIK
184 ref|XP_027775676.2|  98.7%  64.4%   QTEQLTCMSYPFDHFHDSHRYIEHY-ILQ-PETGPLNLIDPIHEFKALTNTDTATEEDIKMKFSNEVFRFASACMNSRTNGTIHFGVKDKPHG---EIIGVKVTN--KDAFINHFNLMIK
185 ref|XP_008570541.1|  98.7%  64.4%   KTELLTCMPYPFDQFHDSKRYIENY-ILQ-PETGPLNLIDPVHEFKALTNTETATEEDIKMKFSNEVFRFASACMNSRTNGTIHFGVKDKPHG---EIVGVKVTS--KDVFIDHFNLMIR
186 ref|XP_008259950.2|  98.7%  63.6%   QTERLTCMPYPFDQFHDSQRYIEHY-ILQ-PETGPLNLIDPIHEFKALTNTAAATEEDIKMKFSNEVFRFASACMNSRTNGTIHFGVKDKPHG---EIVGVKISS--KDVLINHFNVMIK
187 ref|XP_065398409.1|  98.7%  64.0%   KPEQLTCMPYPFDQFHDSHRYIEHY-TLQ-PETGALNLIDPIHEFKALTNTETATEADIKMKFSNEVFRFASACMNSRTNGTIHFGVKDKPHG---EIVGVKINS--KDAFIDHFNLMIK
188 ref|XP_011729134.2|  98.7%  64.0%   KPEQLTCMPYPFDQFHDSHRYIEHY-TLQ-PETGALNLIDPIHEFKALTNTETATEADIKMKFSNEVFRFASACMNSRTNGTIHFGVKDKPHG---EIVGVKINS--KDAFIDHFNLMIK
189 ref|XP_027973811.1|  98.7%  63.1%   ETEQLTCMPYPFDQFHNSQRYIEHS-ILQ-PETGPLNLIDPIHEFKALTNTEAATEKDIKMKFSNEVFRFASACMNSRTNGTIHFGVKDRPHG---QIVGVKVTS--KDAFINHFNIMIR
190 ref|XP_014643659.1|  98.7%  63.1%   ETEQLACMPYPFDQFHDSHRYIEHY-ILQ-PETGPLNLIDPIHEFKALTNTETATEKDIKMKFSNEVFRFASACMNSRTNGTIHFGVKDKPHG---EIVGVKVTS--KDSFIDHFNIMIK
191 ref|XP_032108853.1|  98.7%  64.0%   KPEQLTCMPYPFDQFHDSQRYIEHY-ILQ-PETGPLNLIDPIHEFKALTDTERATEEDIKMKFSNEVFRFASACMNSRTNGTIHFGVKDKPHG---KIVGVNITS--KDVFIDHFNLMIK
192 ref|XP_066094273.1|  98.3%  63.3%   KPEQLTCIPYPFDHFHCSHRYIEHYVL-Q-PETGPLNLIDPVHEFKAFTNTENATEKDIKMKFSNEVFRFTSACMNSRTNGTIHFGVKDKPHG---EIVGVQVTS--KEAFIDHFNLMIK
193 ref|XP_028381763.1|  98.7%  63.1%   KPEQLTCMPHPFDQPQSSHRYIEHY-LLP-PETGPGNLIDPIHEYKALTHTDTATEKDIRMKFSNEVFRFASACMNSRTNGTIHFGVKDKPHG---EIVGVKVTS--KAAFIDHFNQMIT
194 ref|XP_046526743.1|  98.7%  63.6%   QAEQLTCMPYPFDQFHDSQRYIEHY-ILQ-PETGPLNLIDPIHEFKALTNTETATEKDIKMKFSNEAFRFASACMNSRTNGTIHFGVKNKPHG---EIVGVKVTS--KDSFIDHFNVMIK
195 ref|XP_058381591.1|  98.7%  63.1%   ETEQLACMPYPFDQFHDSHRYIEHY-ILQ-PETGPLNLIDPIHEFKALTNTETATEKDIKMKFSNEVFRFASACMNSRTNGTIHFGVKDKPHG---EIVGVKVTS--KDSFIDHFNIIIK
196 ref|XP_027429555.1|  98.7%  62.3%   ETEQLTCMPYPFDQFHNSQRYIEHS-ILQ-PETGPLNLIDPIHEFKALTNTEAATEKDIKMKFSNEVFRFASACMNSRTNGTIHFGVKDRPHG---QIVGVKVTS--KDAFINHFNIMIR
197 ref|XP_017380582.1|  98.7%  63.6%   KPEQLTCMPYPFDQFHDSQRYIEHY-ILQ-PETGPLNLIDPIHEFKALTDTERATEEDIKMKFSNEVFRFASACMNSRTNGTIHFGVKDKPHG---EIVGVNITS--KDVFIDHFNLMIK
198 ref|XP_066210387.1|  98.3%  63.3%   KPERLTCIPYPFDHFHSSHRYIEHYVL-Q-PETGPLNLIDPVHEFKAFTNTENATEKDIKMKFSNEVFRFTSACMNSRTNGTIHFGVKDKPLG---EIVGVQVTN--KEAFIDHFNLMIK
199 ref|XP_016074281.1|  98.7%  64.4%   KIEQLTCMPYPFDQFHSCHRYIEHY-LIQ-PETGPLNLIDPIHEFKALTNTEAATEKDIRMKFSNEVFRFASACMNSRTNGTIHFGVMDKPHG---EIVGVQVTN--KNDFIDHFNTMIK
200 ref|NP_001244057.1|  98.7%  63.6%   QAEQLTCMPYPFDQFHDSQRYIEHY-ILQ-PETGPLNLIDPIHEFKALTNTETATEKDIKMKFSNEAFRFASACMNSRTNGTIHFGVKNKPHG---EIVGVKVTS--KDSFIDHFNVMIK
201 ref|XP_040821033.1|  98.7%  62.7%   QAEQLTCMPYPFDQFHASHRYIERH-CLQ-PETGPLNLIDPIHEFKALTNTETATEEDVKMKFSNEVFRFASACMNSRTNGTIHFGVKDNPHG---EIVGVNISS--KDALIDHFNLMIK
202 ref|XP_063098771.1|  98.3%  62.0%   KTENLTCPPYPFDNFHDGQRYIEHY-ILQ-PETGPLNLIDPIHEFKALTNTENATEEDLKMKFSNETFRFASACMNSRTNGTIHFGVKDKPH---GEIVGVKVTS--KDAFIDHFNLMIK
203 ref|XP_007980344.1|  98.7%  64.0%   KPEQLTCMPYPFDQFHDSHRYIEHY-TLQ-PETGALNLIDPIHEFKALTNTETATEADIKMKFSNEVFRFASACMNSRTNGTIHFGVKDKPHG---EIVGVKITS--KDAFIDHFNLMIK
204 ref|XP_070471752.1|  98.7%  63.6%   QAEQLTCMPYPFDQFHDSQRYIEHY-ILQ-PETGPLNLIDPIHEFKALTNTETATEKDIKMKFSNEAFRFASACMNSRTNGTIHFGVKNKPHG---EIVGVKVTS--KDSFIDHFNVMIK
205 ref|XP_017812450.3|  98.7%  63.6%   KPEQLTCMPYPFDQFHDSHRYIEHY-TLQ-PETGALNLIDPIHEFKALTNTETATEADIKMKFSNEVFRFASACMNSRTNGTIHFGVKDKPHG---EIVGVKITS--KDAFIDHFNLMIK
206 ref|XP_011853657.1|  98.7%  63.6%   KPEQLTCMPYPFDQFHDSHRYIEHY-TLQ-PETGALNLIDPIHEFKALTNTETATEADIKMKFSNEVFRFASACMNSRTNGTIHFGVKDKPHG---EIVGVKITS--KDAFIDHFNLMIK
207 ref|XP_026247361.1|  98.7%  63.1%   QTEQLTCMSYPFDHFHDSHRYIEHY-ILQ-PETGPLNLIDPIHEFKALTNTDTATEEDIKMKFSNEVFRFASACMNSRTNGTIHFGVKDKPHG---EIIGVKVTS--KDAFINHFNLMIK
208 ref|XP_012306232.2|  98.7%  63.1%   KPEQLTCMPYPFDQFHDSQRYIEHY-TLQ-PETGPLNLIDPIHEFKALTDTERATEEDIKMKFSNEVFRFASACMNSRTNGTIHFGVKDKPHG---EIVGVNITS--KDAFIDHFNLMIK
209 ref|XP_024432967.2|  98.7%  62.3%   KPEQLTCMPHPFDQAQSSHRYIEHY-LLP-PETGPGNLIDPIHEYKALTNTDTATEKDIKMKFSNEVFRFASACMNSRTNGTIHFGVKDKPHG---EIVGVKVTS--KAAFVDHFNRMIT
210 ref|XP_008062456.1|  98.7%  64.4%   NMGQPTCMPYPFDEFHNSHRYIEHC-ILQ-PETGPLNLIDPIHEFKALTNTDTATKEDIMMKFSNEVFRFASACMNSRTNGTIHFGVKDKPHG---EIVGVKVTS--KDAFIDHFNHMIK
211 ref|XP_020016743.1|  98.7%  61.9%   KTEQLTCMTYPFDHFHESHRYTEHH-ILQ-PETGPLNLIDPIHEFKALTNTERATEEDIKMKFTNEVFRFASACMNSRTNGTIHFGVKDKPHG---EIVGVKVTS--KNVFIDHFNLMIK
212 ref|XP_045684040.1|  98.7%  62.3%   KPEQLTCMPHPFDQPQSSHRYIEHY-LLP-PETGPGNLIDPIHEYKALTHTDTATEKDIRMKFSNEVFRFASACMNSRTNGTIHFGVKDKPHG---EIVGVKVTS--KAAFIDHFNQMIT
213 ref|XP_011929253.1|  98.7%  63.1%   KPEQLTCMPYPFDQFHDSHRYIEHY-TLQ-PETGALNLIDPIHEFKALTNTETATEADIKMKFSNEVFRFASACMNSRTNGTIHFGVKDKPHG---EIVGVKITS--KDAFIDHFNLMIK
214 ref|XP_004582358.2|  98.7%  62.3%   QAGQLTCMPYPFDQFHASHRYIERH-LLQ-PETGPLNLIDPIHEFKALTNTGTATEEDIKMKFSNEVFRFASACMNSRTNGTIHFGVRDNPHG---EIVGVNISS--KDALIDHFNLMIK
215 ref|XP_053511032.1|  98.7%  62.7%   KPEQLTCMPHPFDQPQSSHQYIEHY-LLP-PETGPGNLIDPIHEYKALTNTDTATEKDIKMKFSNEVFRFASACMNSRANGTIHFGVKDKPHG---EIVGVKVTS--KAAFIDHFNRMIT
216 ref|XP_025237015.1|  98.7%  63.1%   KPEQLTCMPYPFDQFHDSHRYIEHY-TLQ-PETGALNLIDPIHEFKALTNTETATEADIKMKFSNEVFRFASACMNSRTNGTIHFGVKDKPHG---EIVGVKITS--KDAFIDHFNLMIK
217 ref|XP_009451890.1|  98.7%  63.1%   KPEQLTCMPYPFDQFHDSHRYIEHY-TLQ-PETGALNLIDPIHEFKALTNTETATEVDIKMKFSNEVFRFASACMNSRTNGTIHFGVKDKPHG---EIVGVKITS--KAAFIDHFNVMIK
218 ref|NP_001290425.1|  98.7%  63.1%   KPEQLTCMPYPFDQFHDSHRYIEHY-TLQ-PETGALNLIDPIHEFKALTNTETATEVDIKMKFSNEVFRFASACMNSRTNGTIHFGVKDKPHG---EIVGVKITS--KAAFIDHFNVMIK
219 ref|XP_008960246.3|  98.7%  63.1%   KPEQLTCMPYPFDQFHDSHRYIEHY-TLQ-PETGALNLIDPIHEFKALTNTETATEVDIKMKFSNEVFRFASACMNSRTNGTIHFGVKDKPHG---EIVGVKITS--KAAFIDHFNVMIK
220 ref|XP_012415619.1|  98.7%  61.4%   ETEQLTCMPYPFDQFHNSQRYIEHS-ILQ-PETGPLNLIDPIHEFKALTNTEAATEKDIKMKFSNEVFRFASACMNSRTNGTIHFGVKDRPHG---QIVGVKVTS--KDAFIHHFNIMIR
221 ref|XP_008684459.2|  98.7%  62.3%   ETEQLTCMPYPFDQFHNSQRYIEHS-ILQ-PETGPLNLIDPIHEFKALTNTEAATEKDIKMKFSNEVFRFASACMNSRTNGTIHFGVKNKPHG---QIVGVKVTS--KDAFIDHFNVMIR
222 ref|XP_036902380.1|  98.7%  61.4%   KPEQLTCMPHPFDQPQSSHWYIEHY-LLP-PETGPGNLIDPIHEYKALTNTDTGTEKDIKMKFSNEVFRFTSACMNSRANGTIHFGVKDKPHG---EIVGVKVTS--KAAFIDHFNQMIK
223 ref|XP_026361422.1|  98.7%  62.3%   ETEQLTCMPYPFDQFHNSQRYIEHS-ILQ-PETGPLNLIDPIHEFKALTNTEAATEKDIKMKFSNEVFRFASACMNSRTNGTIHFGVKNKPHG---QIVGVKVTS--KDAFIDHFNVMIR
```

224	ref|XP_002918280.1|	98.7%	62.3%	ETEQLTCMPYPFDQFHNSQRYIEHS-ILQ-PETGPLNLIDPIHEFKALTNTEAATEKDIKMKFSNEVFRFASACMNSRTNGTIHFGVKNKPHG---QIVGVKVTS--KDAFIDHFNVMIR
225	ref|XP_054351924.1|	98.7%	62.7%	KPEQLTCMPYPFDQFHDSHRYIEHY-TLQ-PETGALNLIDPIHEFKALTNTETATEADINMKFSNEVFRFASACMNSRTNGTIHFGVKDKPHG---EIVGVKITN--KASFIDHFNVMIK
226	ref|XP_009241290.3|	98.7%	62.7%	KPEQLTCMPYPFDQFHDSHRYIEHY-TLQ-PETGALNLIDPIHEFKALTNTETATEADINMKFSNEVFRFASACMNSRTNGTIHFGVKDKPHG---EIVGVKITN--KASFIDHFNVMIR
227	ref|XP_037692600.1|	98.7%	62.7%	KTERLTCMPYPFDRYHEGQRYTEHY-ILP-SETGTLNFIDPIHEFKALTNTEMATEKDIKMKFSNEVFRFASACMNSRTNGTIHFGVKDKPHG---EIVGVTVTN--KEAFIDHFNLMIR
228	ref|XP_037692601.1|	98.7%	62.7%	KTERLTCMPYPFDRYHEGQRYTEHY-ILP-SETGTLNFIDPIHEFKALTNTEMATEKDIKMKFSNEVFRFASACMNSRTNGTIHFGVKDKPHG---EIVGVTVTN--KEAFIDHFNLMIK
229	ref|XP_010343634.2|	98.7%	62.7%	KPEQLTCMPYPFDQFHDSQRYIEHY-TLQ-PETGPLNLIDPIHEFKALTDTERATEEDIKMKFSNEVFRFASACMNSRTNGTIHFGVKDKPHG---EIVGVNITS--KDVFIDHFNLMIK
230	ref|XP_021545299.1|	98.7%	62.7%	ETEQLTCMPYPFDQFHNSQRYIEHR-ILQ-PETGPLNLIDPIHEFKALTNTEAATEKDIKMKFSNEVFRFASACMNSRTNGTIHFGVKDRPHG---QIVGVKVTS--KDAFIDHFNIMIR
231	ref|XP_047418554.1|	98.7%	61.9%	KTEQLTCMPYPFDNFCDSRRYIEHY-ILQ-PETGPLNLIDPIHEFEALTNTDTATEEDIKMKFSNEVFRFASACMNSRTNGTIHFGVKDKPHG---EIIGVKVTR--KDVFINHFNLMIK
232	ref|XP_047418561.1|	98.7%	61.9%	KTEQLTCMPYPFDNFCDSRRYIEHY-ILQ-PETGPLNLIDPIHEFEALTNTDTATEEDIKMKFSNEVFRFASACMNSRTNGTIHFGVKDKPHG---EIIGVKVTR--KDVFINHFNLMIK
233	ref|XP_025728670.1|	98.7%	61.2%	ETQQLTCMPYPFDRFHNSQRYIEHS-ILQ-PETGPLNLIDPIHEFKALTNTEAATEKDIKMKFSNEVFRFASACMNSRTNGTIHFGVKDRPHG---QIVGVKVTC--KDAFINHFNIMIK
234	ref|XP_018887315.2|	98.7%	62.3%	KPEQLTCMPYPFDQFHDSHRYIEHY-TLQ-PETGALNLFDPIHEFKALTNTETATEVDIKMKFSNEVFRFASACMNSRTNGTIHFGVKDKPHG---EIVGVKITS--KAAFIDHFNVMIK
235	ref|XP_010601767.1|	98.3%	62.9%	KTEYLTCMPYPFDNFHDSQRYIEHY-ILQ-PETGPLNLIDPIHEFKALTNTENATEEDLKMKFSNEAFRFASACMNSRTNGTIHFGVKDKPH---GEIVGVKVTI--KEAFIDHFNLMIK
236	ref|XP_036902379.1|	98.7%	61.0%	KPEQLTCMPHPFDQPQSSHWYIEHY-LLP-PETGPGNLIDPIHEYKALTNTDTGTEKDIKMKFSNEVFRFTSACMNSRANGTIHFGVKDKPHG---EIVGVKVTS--KAAFIDHFNQMIK
237	ref|XP_070280369.1|	98.7%	63.3%	KAEQLTCMPYPFDQFHSSHRYIEHYLLIQ-PETGPLNLIDPLHEFKALTNTDTATEKDIKMKFSNEVFRFASACMNSRTNGTIHFGILDKPHG---EIVGMEVTS--KDAFIDHFNIMIK
238	ref|XP_032616947.1|	98.7%	62.7%	KPEQLTCMPYPFDQFHDSHRYIEHY-TLQ-PETGALNLIDPIHEFKALTNTETATEADIKMKFSNEVFRFASACMNSRTNGTIHFGVKDNPHG---EIVGVKITS--KAAFIDHFNVMIK
239	ref|XP_030677948.1|	98.7%	62.7%	KPEQLTCMPYPFDQFHDSHRYIEHY-TLQ-PETGALNLIDPIHEFKALTNTETATEADIKMKFSNEVFRFASACMNSRTNGTIHFGVKDNPHG---EIVGVKITS--KAAFIDHFNVMIK
240	ref|XP_036184071.1|	98.7%	63.3%	KAEQLTCMPYPFDQFHSSHRYIEHYLLIQ-PETGPLNLIDPIHEFKALTNTDTATEKDIKMKFSNEVFRFASACMNSRTNGTIHFGILDKPHG---EIVGMEVTS--KDAFIDHFNIMIK
241	ref|XP_004776483.1|	98.7%	62.3%	QTIHLTCMPYPFDQFHNSQRYIEHG-ILQ-PETGPLNLIDPIHEFKALTNTEAATEKDIKMKFSNEVFRFASACMNSRTNGTIHFGVKNTPHG---QIVGVKVTN--KDAFIDHFNIMIR
242	ref|XP_004389713.1|	98.7%	63.1%	KTEQLTCMPYPFDQFHDGRRYIEHY-MLQ-PETGPLNLIDPIHEFKALTNTDMATEKDIQMKFSNEVFRFASACMNSRTNGTIHFGVKDKPHG---EIVGVKVTS--KDAFIDHFNLMIK
243	ref|XP_049749840.1|	98.7%	62.7%	KTEQLTCMPYPFDQFHDSQRYTEHY-ILQ-PETGPLNLIDPIHEFKALTNTDTATEKDIHMKFSNEVFRFASACMNSRTNGTIHFGVKDKPHG---EIVGVKVTS--KDAFINHFNLMIK
244	ref|XP_059252834.1|	98.7%	62.3%	QTIHLTCMPYPFDQFHNSQRYIEHG-ILQ-PETGPLNLIDPIHEFKALTNTEAATEKDIKMKFSNEVFRFASACMNSRTNGTIHFGVKNTPHG---QIVGVKVTN--KDAFIDHFNIMIR
245	ref|XP_033050274.1|	98.7%	62.7%	KPEQLTCMPYPFDKFHDSHRYIEHY-TLQ-PETGALNLIDPIHEFKALTNTETATEVDIKMKFSNEVFRFASACMNSRTNGTIHFGVKDKPHG---EIVGVKITS--KDAFIDHFNLMIK
246	ref|XP_055128669.1|	98.7%	62.3%	KPEQLTCMPYPFDQFHDSHRYIEHY-TLQ-PETGALNLIDPIHEFKALTNTETATEADIKMKFSNEVFRFASACMNSRTNGTIHFGVKDNPHG---EIVGVKITS--KAAFIDHFNVMIK
247	ref|XP_010351460.2|	98.7%	62.7%	KPEQLTCMPYPFDKFHDSHRYIEHY-TLQ-PETGALNLIDPIHEFKALTNTETATEVDIKMKFSNEVFRFASACMNSRTNGTIHFGVKDKPHG---EIVGVKITS--KDAFIDHFNLMIK
248	ref|XP_019585266.1|	98.7%	62.4%	KTEQLTCMPYPFDQFHESKSYIEHYLLAQ-PETGPLNLIDPIHEFKALTNTETATEKDIKMKFSNEVFRFASACMNSRTNGTIHFGVKDKPHG---EIVGVEVTS--KNAFVDHFNLMIK
249	ref|XP_046953376.1|	98.3%	62.3%	ETEQLTCMPYPFDQFHNSQRYIEHS-ILQ-PETGPLNLIDPIHEFKALTNTEAATEKDIKMKFSNEVFRFAAACINSRTNGTIHFGVKNKPHG---QIVGVKVTS--KDAFIDHFNITIR
250	ref|XP_011361946.1|	98.7%	61.9%	KTEQLTCMPYPFDQFHDSQRYIEHY-LIQ-PETGPLNLIDPIHEFKALTNTETATEKDIKMKFSNEVFRFTSACMNSRTNGTIHFGVKDKPHG---EIVGVEVPS--KDAFIDHFNQMIK
251	ref|XP_040328613.1|	98.3%	62.3%	ETEQLTCMPYPFDQFHNSQRYIEHS-ILQ-PETGPLNLIDPIHEFKALTNTEAATEKDIKMKFSNEVFRFAAACINSRTNGTIHFGVKNKPHG---QIVGVKVTS--KDAFIDHFNITIR
252	ref|XP_032160436.1|	98.7%	62.3%	QTMHLTCMPYPFDQFHNSQRYIEHG-ILQ-PETGPLNLIDPIHEFKALTNTEAATEKDIKMKFSNEVFRFASACMNSRTNGTIHFGVKNTPHG---QIVGVKVTN--KDAFIDHFNIMIR
253	ref|XP_003982796.1|	98.3%	62.3%	ETEQLTCMPYPFDQFHNSQRYIEHS-ILQ-PETGPLNLIDPIHEFKALTNTEAATEKDIKMKFSNEVFRFAAACINSRTNGTIHFGVKNKPHG---QIVGVKVTS--KDAFIDHFNITIR
254	ref|XP_014922007.2|	98.3%	62.3%	ETEQLTCMPYPFDQFHNSQRYIEHS-ILQ-PETGPLNLIDPIHEFKALTNTEAATEKDIKMKFSNEVFRFAAACINSRTNGTIHFGVKNKPHG---QIVGVKVTS--KDAFIDHFNITIR
255	ref|XP_025785047.1|	98.3%	62.3%	ETEQLTCMPYPFDQFHNSQRYIEHS-ILQ-PETGPLNLIDPIHEFKALTNTEAATEKDIKMKFSNEVFRFAAACINSRTNGTIHFGVKNKPHG---QIVGVKVTS--KDAFIDHFNITIR
256	ref|XP_030163818.1|	98.3%	62.3%	ETEQLTCMPYPFDQFHNSQRYIEHS-ILQ-PETGPLNLIDPIHEFKALTNTEAATEKDIKMKFSNEVFRFAAACINSRTNGTIHFGVKNKPHG---QIVGVKVTS--KDAFIDHFNITIR
257	ref|XP_047698808.1|	98.3%	62.3%	ETEQLTCMPYPFDQFHNSQRYIEHS-ILQ-PETGPLNLIDPIHEFKALTNTEAATEKDIKMKFSNEVFRFAAACINSRTNGTIHFGVKNKPHG---QIVGVKVTS--KDAFIDHFNITIR
258	ref|XP_043444794.1|	98.3%	62.3%	ETEQLTCMPYPFDQFHNSQRYIEHS-ILQ-PETGPLNLIDPIHEFKALTNTEAATEKDIKMKFSNEVFRFAAACINSRTNGTIHFGVKNKPHG---QIVGVKVTS--KDAFIDHFNITIR
259	ref|XP_025859549.1|	98.7%	60.2%	ETKQLTCMPYPFDQFCNSHCYIENS-VLQ-PETGPLNLIDPIHEFKALTNTEAATEKDIKMKFSNEVFRFASACMNSRTNGTIHFGVKNRPHG---QIVGVKVAH--KDAFIDHFNIMIR
260	ref|XP_011810448.1|	98.7%	62.3%	KPEQLTCMPYPFDQFHDSHRYIEHY-TLQ-PETGALNLIDPIHEFKALTNTGTAAELDIKMKFSNEVFRFASACMNSRTNGTIHFGVKDKPHG---EIVGVKITG--KDAFIDHFNLMIK
261	ref|XP_032258048.1|	98.7%	61.4%	ETEQLTCMPYPFDQFHNSQRYIEHS-ILQ-PETGPLNLIDPIHEFKALTNTEAATEKDIKMKFSNEVFRFASACMNSRTNGTIHFGVKNRPHG---QIVGVKVTS--KDAFIDHFNIMIR
262	ref|XP_032944614.1|	98.7%	62.4%	KTEQLTCMPYPFDQFHESKSYIEHYLLAQ-PETGPLNLIDPIHEFKALTNIETATEKDIKMKFSNEVFRFASACMNSRTNGTIHFGVKDKPHG---EIVGVKVTS--KDAFVDHFNLMIK
263	ref|XP_059027098.1|	98.7%	61.9%	QTIHLTCMPYPFDQFHNSQRYIEHR-ILQ-PETGPLNLIDPIHEFKALTNTEAATEKDIKMKFSNEVFRFASACMNSRTNGTIHFGVKNTPHG---QIVGVKVTD--KDAFIDHFNIMIR
264	ref|XP_043821279.1|	98.7%	61.4%	EPKRCSCMPYPFDNFHDNHRYTQHY-ILQ-PETGPLNLIDPVHEYKALTNTETATEEDIKMKFSNEVFRFASACMNSRTNGTIHFGVKDKPHG---EIVGVKITS--KDAFIDHFNRMIK
265	ref|XP_045720657.1|	98.7%	61.4%	ETEQMTCMPYPFDQFHNSQRYIEHS-ILQ-PETGPLNLIDPIHEFKALTNTEAATEKDIKMKFSNEVFRFASACMNSRTNGTIHFGVKDRPHG---QIVGVKVTS--KDAFIDHFNIMIR
266	ref|XP_062034304.1|	97.8%	62.3%	QTEQLTCMPYPFDQFHDSQRYIEHY-ILQ-PETGPLNLIDPIHEFKALTNTSTVTE--MKMKFSNEVFRFASACMNSRTNGTIHFGVNDKPHG---EIVGVKISR--KDVLIDHFNEMIK
267	ref|XP_049498825.1|	98.3%	62.3%	ETEQLTCMPYPFDQFHNSQRYIEHS-ILQ-PETGPLNLIDPIHEFKALTNTEAATEKDIKMKFSNEVFRFAAACINSRTNGTIHFGVKNKPHG---QIVGVKVTS--KDAFIDHFNITIR
268	ref|XP_034876457.1|	98.7%	61.4%	ETEQMTCMPYPFDQFHNSQRYIEHS-ILQ-PETGPLNLIDPIHEFKALTNTEAATEKDIKMKFSNEVFRFASACMNSRTNGTIHFGVKDRPHG---QIVGVKVTS--KDAFIDHFNIMIR
269	ref|XP_023082516.2|	98.7%	61.9%	KPEQLTCMPYPFDQFHDSHRYIEHY-TLQ-PETGALNLIDPIHEFKALTNTETAAELDIKMKFSNEVFRFASACMNSRTNGTIHFGVKDKPHG---EIVGVKITS--KDAFIDHFNLMIK
270	ref|XP_007076766.2|	98.3%	62.3%	ETEQLTCMPYPFDQFHNSQRYIEHS-ILQ-PETGPLNLIDPIHEFKALTNTEAATEKDIKMKFSNEVFRFAAACINSRTNGTIHFGVKNKPHG---QIVGVKVTS--KDAFIDHFNITIR
271	ref|XP_045350732.1|	98.3%	62.3%	ETEQLTCMPYPFDQFHNSQRYIEHS-ILQ-PETGPLNLIDPIHEFKALTNTEAATEKDIKMKFSNEVFRFAAACINSRTNGTIHFGVKNKPHG---QIVGVKVTS--KDAFIDHFNITIR
272	ref|XP_058581065.1|	98.3%	62.3%	ETEQLTCMPYPFDQFHNSQRYIEHS-ILQ-PETGPLNLIDPIHEFKALTNTEAATEKDIKMKFSNEVFRFAAACINSRTNGTIHFGVKNKPHG---QIVGVKVTS--KDAFIDHFNITIR
273	ref|XP_016016798.2|	98.7%	61.4%	KTEQLTCMPYPFDQFHDSQRYIERY-FIQ-PETGPLNLIDPIHEFKALTNTETATEKDIKMKFSNEVFRFTSACMNSRTNGTIHFGVKDKPHG---EIVGVEVPS--KDAFIDHFNLMIK
274	ref|XP_069852600.1|	98.7%	62.7%	EAEQFTCMPYPFDRFHDSQRYIEHY-ILQ-PETGPLNLIDPIHEFKALTNIEMATEEDVKMKFTNEVFRFASACMNSRTNGTIHFGVKDKPHG---EIVGVKVTS--KDVFIDHFNVMIK
275	ref|XP_015444939.1|	98.7%	61.4%	KTEQLTCMPYPFDQFHDSQRYIEHY-LIQ-PETGPLNLIDPIHEFKALTNTETATEKDIKMKFSNEVFRFTSACMNSRTNGTIHFGVKDKPHG---EIVGVEVPS--KDAFIDHFNQMIK
276	ref|XP_036875742.1|	98.7%	59.3%	KTEQLTCMPYPFDKFHESKRYIEHY-ILQ-PETGPLNLIDPVHEFKALTNTEAASDKDIRMKFSNEVFRFASACMNSRTNGTIHFGVLDKPHG---EIIGVKVTS--KDAFIDHFNIMIK
277	ref|XP_039734028.1|	98.7%	61.4%	KTEQLTCMPYPFDQFHDSQRYIEHY-LIQ-PETGPLNLIDPIHEFKALTNTETATEKDIKMKFSNEVFRFTSACMNSRTNGTIHFGVKDKPHG---EIVGVEVPS--KDAFIDHFNQMIK
278	ref|XP_042540451.1|	98.7%	62.7%	EAEQFTCMPYPFDRFHDSQRYIEHY-ILQ-PETGPLNLIDPIHEFKALTNIEMATEEDVKMKFTNEVFRFASACMNSRTNGTIHFGVKDKPHG---EIVGVKVTS--KDVFIDHFNVMIK
279	ref|XP_041583429.1|	98.7%	60.2%	ETKQLTCMPYPFDQFCNSHCYIENS-VLQ-PETGPLNLIDPIHEFKALTNTEAATEKDIKMKFSNEVFRFASACMNSRTNGTIHFGVKNRPHG---QIVGVKVAH--KDAFIDHFNIMIR
280	ref|XP_006891533.1|	98.7%	61.0%	APEKLTCLPYPFDKFHDSRRYIEHY-ILQ-PETGPLNLIDPIHEFKAFTNTSTATAKDIQMKFSNEVFRFASACMNVRTNGTIHLGVKDKPHG---EIVGVSVTS--KDAFIDHFNLMIN
281	ref|XP_038412756.1|	98.7%	60.6%	GIKQLTCMPYPFDQFHNSHCYIENS-VLQ-PETGPLNLIDPIHEFKALTNTEAATEKDIKMKFSNEVFRFASACMNSRTNGTIHFGVKNRPHG---QIVGVKVAN--KDAFIDHFNIMIR
282	ref|XP_025327152.1|	98.7%	60.6%	GIKQLTCMPYPFDQFHNSHCYIENS-VLQ-PETGPLNLIDPIHEFKALTNTEAATEKDIKMKFSNEVFRFASACMNSRTNGTIHFGVKNRPHG---QIVGVKVAN--KDAFIDHFNIMIR
283	ref|XP_038542377.1|	98.7%	60.6%	GIKQLTCMPYPFDQFHNSHCYIENS-VLQ-PETGPLNLIDPIHEFKALTNTEAATEKDIKMKFSNEVFRFASACMNSRTNGTIHFGVKNRPHG---QIVGVKVAN--KDAFIDHFNIMIR
284	ref|XP_012881542.1|	98.7%	62.3%	EAEQFTCMPYPFDQFHDSQRYIEHY-ILQ-PETGPLNLIDPIHEFKALTNIEMATEEDVKMKFTNEVFRFASACMNSRTNGTIHFGVKDKPHG---EIVGVKVAS--KDVFIDHFNVMIK
285	ref|XP_055163576.1|	98.7%	60.2%	ETKQLTCMPYPFDQFHNSHCYIENS-VLQ-PETGPLNLIDPIHEFKALTNTEAATEKDIKMKFSNEVFRFASACMNSRTNGTIHFGVKNRPHG---QIVGVKVTS--KDAFIDHFNITIR
286	ref|XP_054582508.1|	98.7%	62.9%	KAEQLTCMPYPFDQFHSSHRYIEHYILIQ-PETGPLNLIDPIHEFKSLTNTETATEKDIKMKFSNEVFRFASACMNSRTNGTIHFGVLDKPHGAHGEIVGMEVTS--KDAFIDHFNIMIK
287	ref|XP_006733000.1|	98.7%	61.4%	ETEQLTCMPYPFDQFHNSQRYIEHR-ILQ-PETGPLNLIDPIHEFKALTNTEAATEKDIKMKFSNEVFRFASACMNSRTNGTIHFGVKDRPHG---QIAGVKVTS--KNAFIDHFNIMIR
288	ref|XP_060488440.1|	98.3%	62.3%	ETEQLTCMPYPFDQFHNSQRYIEHS-ILQ-PETGPLNLIDPIHEFKALTNTEAATEKDIKMKFSNEVFRFAAACINSRTNGTIHFGVKNKPHG---QIVGVKVTS--KDAFIDHFNITIR
289	ref|XP_019298861.2|	98.3%	62.3%	ETEQLTCMPYPFDQFHNSQRYIEHS-ILQ-PETGPLNLIDPIHEFKALTNTEAATEKDIKMKFSNEVFRFAAACINSRTNGTIHFGVKNKPHG---QIVGVKVTS--KDAFIDHFNITIR
290	ref|XP_042773716.1|	98.3%	62.3%	ETEQLTCMPYPFDQFHNSQRYIEHS-ILQ-PETGPLNLIDPIHEFKALTNTEAATEKDIKMKFSNEVFRFAAACINSRTNGTIHFGVKNKPHG---QIVGVKVTS--KDAFIDHFNITIR
291	ref|XP_032731004.1|	98.7%	61.9%	QTKHLTCMPYPFDQFHNSQRYIEHR-ILQ-PETGPLNLIDPIHEFKALTNTEAATENDIKMKFSNEVFRFASACMNSRTNGTIHFGVKNKPHG---QIVGVKVTD--KDAFIDHFNIMIR
292	ref|XP_006163552.1|	98.7%	61.9%	KTEQLTCMPYPFDQFHDSKRYIEHY-ILQ-PETGPLNLIDPIHEFKALTNTETATEESIKMKFSNEVFRFASACMNSRTNGTIHFGVLDKPHG---EIVGVKVAN--KNVFIDHFNLMIK
293	ref|XP_059568319.1|	97.0%	62.4%	KAEQLTCMPYPFDQFHSSHRYIEHYLLIQ-PETGPLNLIDPIHEFKALTNTDTATEKDIKMKFSNEVFRFASACMNSRTNGTIHFGILDKPHG---EIVGIEVTN--KGAFIDHFNTMIK
294	ref|XP_045876181.1|	98.7%	61.4%	QTEHLTCMPYPFDQFHNSQRYIEHR-ILQ-PETGPLNLIDPIHEFKALTNTEAATEKDIKMKFSNEVFRFASACMNSRTNGTIHFGVKNTPHG---QIVGVKVTD--KDAFIDHFNIMIR
295	ref|XP_022359131.1|	98.7%	61.9%	QTKHLTCMPYPFDQFHNSQRYIEHR-ILQ-PETGPLNLIDPIHEFKALTNTEAATEKDIKMKFSNEVFRFASACMNSRTNGTIHFGVKDTPHG---QIVGVKVTD--KDAFIDHFNKMIR
296	ref|XP_005882177.1|	98.7%	62.9%	KAEQLTCMPYPFDQFHSSHRYIEHYLLIQ-PETGPLNLIDPIHEFKALTNTDKATENGIKMKFSNEVFRFASACMNSRTNGTIHFGILDKPHG---EIVGMEVTS--KDAFIDHFNKMIK
297	ref|XP_007522555.1|	98.7%	61.9%	KIVQSTCMPYPFDQFHASHHYTEHY-ILQ-PETGPLNLIDPIHEFKALTNTETATEKDIKMKFSNEVFRFASACMNSRTNGTIHFGVLDKPHG---EIVGVKVRS--KDAFIDHFNLMIK
298	ref|XP_037363842.1|	98.7%	61.4%	KPEQCTCMPYPFDQFHDSIRYIEHY-ILQ-PETGPLNLIDPIHEFKALTETKTASDKDIKMKFSNEVFRFASACMNSRTNGTIHFGVKDKPHG---EIVGIEAPS--KDAFIDHFNLMIN
299	ref|XP_036614332.1|	98.7%	60.8%	EPRRLSCMPYPFDNFHDSCRYIQHY-ILQ-PETGPLNLIDPVHEYKALTNTETATEEDIKMKFSQEVFKFASACMNSRTNGTIHFGVKDKPHG---EIVGLKITN--KEAFVDHFNLMIK
300	ref|XP_023400184.2|	98.7%	61.4%	KTEQLTCMPYPFDQFHDSQRYTEHY-ILQ-PETGPLNLIDPIHEFKALTNTDTATEKDIHMKFSNEVFRFASACMNSRTNGTIHFGVKDKPHG---EIVGVKVAS--KDDFINHFNLMIK
301	ref|XP_003407194.2|	98.7%	61.4%	KTEQLTCMPYPFDQFHDSQRYTEHY-ILQ-PETGPLNLIDPIHEFKALTNTDTATEKDIHMKFSNEVFRFASACMNSRTNGTIHFGVKDKPHG---EIVGVKVAS--KDDFINHFNLMIK
302	ref|XP_044539777.1|	98.7%	61.0%	EPKRLFCMPYPFDDFQDSYRYIQHY-ILQ-PETGPLNLIDPVHEFKALINTEAATEEDIKMKFSNEVFRFASACMNSRTNGTIHFGVKDKPHG---EIVGVKITN--KDAFVDHFNQMIK
303	ref|XP_036735125.2|	98.7%	58.9%	KTEQLTCMPYPFDKFHESKRYIEHY-ILQ-PETGPLNLIDPVHEFKALTNTEAASDKDIRMKFSNEVFRFASACMNSRTNGTIHFGVLDKPHG---EIIGVKVTS--KDAFIDHFNIMIK
304	ref|XP_004646313.1|	98.7%	61.7%	KTEKLPCIPYPFDNFHDSQRYIEHY-ILQ-PETGPLNLIDPIHEFKALTNTENATEKDIKMKFSNEVFKFASACMNSRSNGTIHFGVKDKPHAEHGEIVGVKVTN--KAAFIDHFNQMIK
305	ref|XP_023562287.1|	98.7%	61.7%	KTEKLPCIPYPFDNFHDSQRYIEHY-ILQ-PETGPLNLIDPIHEFKALTNTENATEKDIKMKFSNEVFKFASACMNSRSNGTIHFGVKDKPHAEHGEIVGVKVTN--KAAFIDHFNQMIK
306	ref|XP_020843067.1|	98.7%	59.9%	EPKRLPCMPYPFDNFHDTYRYIQHY-TLQ-VETGPLNLIEPAHEYKALTNTESATEEDIKMKFSNEVFRFASACMNSRTNGTIHFGVKDKPHG---EIIGLKITS--KDVFIDHFNLMIK
307	ref|XP_055974091.1|	98.7%	60.6%	KTEQSTCVPYPFDQFHDSRCYIEHH-ILQ-PETGPRNLIDPIHEFKALTNTESVEEKDIQMKFSNEVFRFASACMNSRTNGTIHFGVLDKPQG---EIVGVKVTR--KDSFIDHFNKMIK
308	ref|XP_005388084.1|	98.3%	59.9%	KTENFTCLPYPFDNFHDSKRYIEHY-ILQ-PETGPLNLIDPIHEFKALTDTEYATEEGLKMKFSNEAFRFASACMNTRTNGTIHFGVKDKPH---GEIVGVKVTN--KGAFIDHFNKMIK
309	ref|XP_068942246.1|	98.7%	59.9%	EPKRLQCMSYPFDNFHESCRYIQHH-TLQ-PETGPRNLIDPIHEYKALTNTNTATEEDIKMKFSNETFKFASACMNSRTNGTIHFGVADKPHG---EIIGLKIIS--KNAFIDHFNLMIK
310	ref|XP_029784994.1|	98.3%	61.4%	KTEQLTCMPYPFDQFHNSQRYVEHS-ILQ-PETGPLNLIDPIHEFKALTNTEAATERDIKMKFSNEVFRFASACMNSRTNGTIHFGVKNKPHG---QIVGVKVTS--KDAFIDHFNITIK
311	ref|XP_029784955.1|	98.3%	61.4%	KTEQLTCMPYPFDQFHNSQRYVEHS-ILQ-PETGPLNLIDPIHEFKALTNTEAATERDIKMKFSNEVFRFASACMNSRTNGTIHFGVKNKPHG---QIVGVKVTS--KDAFIDHFNITIK
312	ref|XP_007938002.1|	98.7%	59.7%	KIEQLTCMPCPFDQFHDSQRYIEHY-ILQ-PETGPLNLIDPIHEFKLLTNTETATEKDIHMKFSNEAFRFASACMNSRTNGTIHFGVKDKPHG---EIVGLKVTS--KDGFIDHFNKIIK
313	ref|XP_059112478.1|	98.7%	61.4%	KTEQMSCMPHPFDSFHDDKRYIEHS-VLQVPETGPLNLIDPVHEFKSLTNTETATEEDIKMKFSNEVFRFASACMNSRTNGTIHFGVGDKPHG---QIVGVKVTS--KDVFINHFNVMIT
314	ref|XP_052577148.1|	98.7%	61.0%	KTEQMSCMPHPFDSFHDDKRYIEHS-VLQVPETGPLNLIDPVHEFKALTNTETATEEDIKMKFSNEVFRFASACMNSRTNGTIHFGVGDKPHG---QIVGVKVTS--KDVFINHFNVMIT
315	ref|XP_006779273.1|	98.7%	62.9%	KTEQLTCMPYPFDQFHSSHRYIEHYLLIQ-PETGPLNLIDPIHEFKALINTDTATEKDIKMKFSNEVFRFASACMNSRTNGTIHFGILDKPHG---EIVGMEVTS--KDAFIDHFNKMIK
316	ref|XP_028725615.1|	98.7%	61.0%	KAGQMSCMPHPFDSFHDDKRYIEHS-VLQVPETGPLNLIDPVHEFKALTNTETATGEDIKMKFSNEVFRFASACMNSRTNGTIHFGVGDKPHG---QIVGVKVTS--KDVFVNHFNVMIT
317	ref|XP_055002314.1|	98.7%	60.6%	KKEQSTCVPYPFDQFHDSRRYVEHQ-ILQ-PETGPRNLIDPIHEFKALTNTESVEEKDIKMKFSNEVFRFASACMNSRTNGTIHFGVLDKPQG---EIVGVKVTR--KDSFIDHFNKMIK
318	ref|XP_059112476.1|	98.7%	60.6%	KTEQMSCMPHPFDSFHDDKRYIEHS-VLQVPETGPLNLIDPVHEFKALTNTETATEEDIKMKFSNEVFRFASACMNSRTNGTIHFGVGDKPHG---QIVGVKVTS--KDVFVNHFNVMIT
319	ref|XP_004702866.1|	98.7%	59.7%	KIEQVTCMPYPFDQFHDSQRYTVHC-ILP-PETGPLNLIDPVHEYKALTNTDTATEKDMLMKFSNEVFRFASACMNSRTNGTIHFGVQDKPHG---EIVGVRVTR--KDAFIDHFNGMIK
320	ref|XP_048195987.1|	98.7%	60.6%	KAEQFTCMPYPFDHFHDSQRYIERY-ILQ-PETGPLNLIDPIHEFKALTNTEMATEEDIKMKFSNEVFRFASACMNSRTNGTIHFGVKDKPHG---EIIGVKVTS--KDAFIDHFNKMIK
321	ref|XP_028725619.1|	98.7%	61.0%	KAEQMSCMPHPFDSFHDDKRYIEHS-VLQVPETGPLNLIDPVHEFKALTNTETATGEDIKMKFSNEVFRFASACMNSRTNGTIHFGVEDKPHG---QIVGVKVTS--KDVFINHFNVMIT
322	ref|XP_044101964.1|	98.7%	61.9%	QTIYLTCMPYPFDQFHNSQRYIEHG-ILQ-PETGPLNLIDPIHEFKALTNTEAATEKDIKMKFSNEVFRFASACMNSRTNGTIHFGVKNTPHG---QIVGVKVTD--KDAFIDHFNIMIR
323	ref|XP_035307523.1|	98.7%	59.7%	KPEQMSCMPYPFDSFHDDRRYIERY-ILQVAETGPLNLIDPVHEFKALTNTETATEEDIKMKFSNEIFRFASACMNSHTNGTIHFGVKDKPHG---EIVGVKIPS--KDVFVNHFNEMIK
324	ref|XP_036285317.1|	98.7%	60.0%	KAEQLTCMPYPFDQFHSSHRYIEHYILIQ-PETGPLNLIDPIHEFKSLINTETATEKDIKMKFSNEVFRFASACMNSRTNGTIHFGVLDKPHGAHGEIVGMEITS--KDAFIDHFNIMIK
325	ref|XP_036285315.1|	98.7%	60.0%	KAEQLTCMPYPFDQFHSSHRYIEHYILIQ-PETGPLNLIDPIHEFKSLINTETATEKDIKMKFSNEVFRFASACMNSRTNGTIHFGVLDKPHGAHGEIVGMEITS--KDAFIDHFNIMIK
326	ref|XP_045434389.1|	98.7%	60.0%	KAEQLTCMPYPFDQFHSSHRYIEHYILIQ-PETGPLNLIDPIHEFKSLINTETATEKDIKMKFSNEVFRFASACMNSRTNGTIHFGVLDKPHGAHGEIVGMEITS--KDAFIDHFNIMIK
327	ref|XP_006991269.2|	98.7%	60.6%	KAEQMSCMPYLFDSFHDDKRYIEHS-VLQVAETGPLNLIDPVHEFKALTNTETATEEDIKMKFSNEVFRFASACMNSRTNGTIHFGVEDKPHG---QIVGVKVTS--KDVFINHFNVMIT
328	ref|XP_021508901.1|	98.7%	60.3%	KPEQMACMPYPFDSFHEAKQYIEHS-ILRVPETGPLNLIDPVHEFKAFTNTEAATEEDLKMKFSNEVFRFAAACMNSRTNGTIHFGVKDKPHG---EIVGVKVTN--KDVFINHFNVMIT
329	ref|XP_035294151.1|	98.7%	59.3%	KPEQMSCMPYPFDSFHDDRRYIERY-ILQVAETGPLNLIDPVHEFKALTNTETATEEDIKMKFSNEIFRFASACMNSHTNGTIHFGVKDKPHG---EIVGVKIPS--KDVFVDHFNKMIK
330	ref|XP_042128701.1|	98.7%	60.2%	KAEQMSCMPHLFDSFHDDKCYIEHS-VLQVPETGPLNLIDPVHEFKALTNTETATEEDIKMKFSNEVFRFASACMNSRTNGTIHFGVEDKPHG---QIVGVKVTS--KDVFVNHFNVMIT
331	ref|XP_006991267.2|	98.7%	60.2%	KAEQMSCMPHLFDSFHDDKCYIEHS-VLQVPETGPLNLIDPVHEFKALTNTETATEEDIKMKFSNEVFRFASACMNSRTNGTIHFGVEDKPHG---QIVGVKVTS--KDVFVNHFNVMIT
332	ref|XP_003771994.1|	98.7%	59.7%	EPKRLPCLPYPFNSFHDTCRYIQHY-ILQ-PETGPLNLIDPVHEFKLLTNTEVATEEDIKMKFSNEVFRFASACMNSRTNGTIHFGVKDKPHG---EIVGMKITN--TDAFIDHFNVKIK
333	ref|XP_039111369.1|	98.3%	60.2%	KTEQLTCMPYPFDQFHNSQRYIEHS-ILQ-PETGPLNLIDPTHEFKALTNTEAATEKDIKMKFSNEVFRFASACMNSRTNGTIHFGVKNKPHG---QIVGVKVTS--KDAFIDHFNNTIR
334	ref|XP_021010545.1|	98.3%	59.5%	KPEQMSCMPYPFDSFCDVKQYIEHS-ILRVAETGPLNLIDPIHEFKAFTNTKKATEEDIKMKFSNETFRFAAACMNSRTNGTIHFGVKDKPHG---EIVGVQVTS--KDIFVNHFNTMIT
335	ref|NP_034286.2|	98.3%	59.5%	KPEQMSCTPYPFDSFCDVKQYIEHS-ILRVAETGPLNLIDPIHEFKAFTNTKKATEEDIKMKFSNETFRFAAACMNSRTNGTIHFGVKDKPHG---EIVGVQVTS--KDIFVNHFNTMIT
336	ref|XP_034374653.1|	98.7%	59.5%	KPEQMSCMPYPFDSFHDVKQYIEHS-ILRVAETGPLNLIDPVHEFKAFTNTEKATEEDIKMKFSNETFRFAAACMNSRTNGTIHFGVKDKPHG---EIVGVQVTN--KDVFVNHFNIMIT
337	ref|XP_051857307.1|	98.7%	58.9%	EPKRLPCLPYPFDSFHDTCRYIQHY-ILQ-PETGPLNFIDPVHEFKLLTNTEAATEEDIKMKFSNEVFRFASACMNSRTNGTIHFGVKDKPHG---EIVGMKITN--TDAFIDHFNVKIK
338	ref|XP_048292669.1|	98.3%	59.7%	KPEHMSCMPHPFDSFHDDRRYIEHS-ILQVAETGPLNLIDPVHEFKALTNTETATEEDIKMKFSNEVFRFASACMNSRTNGTIHFGVKDKPHG---EIVGVKVTS--KDIFINHFNGMIK

339 ref|XP_051007938.1| 98.7% 58.6% KPEQMPCMPYPFNSFHDAKQYIEHA-ILRVAETGPLNLIDPVHEFKAFTNTETATQEDIKMKFSNEVFRFAAACMNSRTNGTIHFGVKDKPHG---EIVGVKVTS--KDVFINHFNVMMT
340 ref|XP_036038446.1| 98.7% 59.3% KAEQMSCMPHPFDSFHDDKCYIEHS-ILQVAETGPLNLIDPVHEFKALTNTETATEEDIKMKFSNEVFRFASACMNSRTNGTIHFGVEDKPHG---QIVGVKVTS--KDVFINHFNVMIT
341 ref|XP_036038795.1| 98.7% 58.1% KAEQMSCMPHPFDSFHDDKRYIEHS-VLQVAETGPLNLIDPVHEFKALTNTETATEVDIKMKFSNEVFRFAAACMNSRTNGTIHFGVEDKPHG---QIVGVKLTS--KDVFINHFNVMIT
342 ref|XP_034375667.1| 98.7% 58.6% KPEQMSCMPYPFNSFHDVKHYIEHA-ILRVAETGPLNLIDPVHEFKAFTNTETATEEDIKMKFSNETFRFAAACMNSRTNGTIHFGVKDKPHG---EIVGVQVTN--KDIFVNHFNIMIT
343 ref|XP_049985914.1| 98.3% 60.2% KPEHMSCMPHPFDSFHDDRRYIEHS-ILQVAETGPLNLIDPVHEFKALTNTETATEEDIKMKFSNEVFRFASACMNSRTNGTIHFGVKDKPHG---EIVGVKVTS--KDIFINHFNGMIK
344 ref|XP_055468922.1| 98.7% 59.5% KPEQMACMPYPFDSFHEAKQYIEHS-ILRVPETGPLNLIDPVHEFKAFTNTEAATEEDLKMKFSNEVFRFAAACMNSRTNGTIHFGVKDKPHG---EVVGVKVTN--KDVFINHFNVMIT
345 ref|XP_016281899.2| 98.7% 57.6% EPKRLFCMPYPFDSFHDSCRYIQHY-ILQ-PETGPLNLIDPVHEFKALINTETATEEDIKMKFSNEVFRFASACMNSRTNGTIHFGVKDKPHG---EIIGMRITN--KDAFVDHFNKMLR
346 ref|XP_040597137.1| 98.3% 59.7% KPQHMSCMPYLFDSFHDDRRYIEHS-ILQVAETGPLNLIDPIHEFKALTNTKTATEEDIKMKFSNEAFRFASACMNSRTNGTIHFGIKDKPHG---EIVGVKVPS--KDVFINHFNEMIK
347 ref|XP_027703736.1| 98.7% 57.8% EPKRLPCMPYPFDKFHDSYRYIQHY-ILQ-PETGRLNFIEPVHEYKALTNTGSATEEDIKMKFSNEVFRFASACMNSRTNGTIHFGVKDKPHG---EIIGLKITS--KDVFIDHFNLMIK
348 ref|XP_041531603.1| 98.3% 59.7% KPEHMSCMPHPFDSFHDDRRYIEHS-ILQVAETGPLNLIDPVHEFKALTNTETATEEDIKMKFSNEVFRFASACMNSRTNGTIHFGVNDKPHG---EIVGMKVTS--KDIFINHFNGMIK
349 ref|XP_028635008.1| 98.7% 58.2% KPEQMSCMPYPFNSFHDVKHYIEHS-ILRVAETGPLNLIDPVHEFKALTNVETATQEDIKMKFSNETFRFAAACMNSRTNGTIHFGVKDKPHG---EIVGVQVTS--KDVFINHFNVMIT
350 ref|XP_006834357.1| 98.7% 57.6% KTQPVTCMPYPFDQFHAGQRYTEHC-VLP-PETGPLNLIDPIHEYKEFTNTDNATEKDSLMKFSNEVFKFASACMNSRTNGTIHFGVMDKPHG---EIVGVKITD--KDAFINHFNLMIQ
351 ref|XP_038176710.1| 98.3% 59.7% KPEHMSCMPHLFDSFHDDRSYIEHS-ILQVAETGPLNLIDPVHEFKALTNTETATEEDIKMKFSNEVFRFASACMNSRTNGTIHFGVKDKPHG---EIVGVKVTN--KDIFINHFNGMIK
352 ref|XP_051033136.1| 98.3% 58.5% KLEQMSCMPHLFDSFHDDRCYIEHS-ILQIAETGPLNLIDPVHEFKALTNTETATEEDIKMKFSNEVFRFASACMNSRTNGTIHFGVEDKPHG---EIVGVKVPS--KDVFINHFNVMIK
353 ref|XP_052029158.1| 98.7% 57.4% KPEQMSCMPYPFDSFHDVKQYIEHS-ILRVAETGPLNLIDPVHEFKAFTNIEQATQEDIKMKFSNETFRFAAACMNSRTNGTIHFGVMDKPHG---EIVGVHITS--KDVFINHFNIMIT
354 ref|XP_021047211.1| 98.7% 58.2% KPEQMSCMPYPFNYFHDVKQYIEHS-ILRVAETGPLNLIDPVHEFKAFTNTEKATEEDVKMKFSNETFRFAAACMNSRTNGTIHFGIKDKPHG---EIVGVQVTS--KDAFINHFNAMIT
355 ref|XP_057645554.1| 98.3% 59.3% KPEHMSCMPHLFDSFHDDKSYIEHS-ILQVAETGPLNLIDPVHEFKALTNTETATEEDIKMKFSNEVFRFASACMNSRTNGTIHFGVKDKPHG---EIVGVKVTS--KDIFINHFNGMIK
356 ref|XP_020843064.1| 97.4% 58.7% ---GQSCLTYPFDKFHKSFRYKEHDVIIQ-PETGLLNLIDPVHEFKALTDTEGATEKDIKMKFSNEVFRFASACMNCRTNGTIHFGVKDKPHG---EIVGVKLTEKDKSAFIDHFYKKME
357 ref|XP_027703686.1| 97.4% 57.9% ---GQSCSTHPFDQFHKSFRYKEHDVIIQ-PETGLLNLIDPVHEFKALTDTEGATEKDIKMKFSNEVFRFASACMNCRTNGTIHFGVKDKPHG---EIVGVKLAKEDKSTFIDHFYKKIE
358 ref|XP_001378512.2| 97.0% 56.6% ---GQTCQTYPFDKFHKSFRYRE-QTIIQ-PETGSFNLIDPIHEFKALTNTERATEEDILMKFSNEVFRFASACMNCRTNGTIHFGVKDSPHG---EIVGLKLTEEDKEKFVDHFYKKIE
359 ref|XP_036617339.1| 97.4% 57.9% ---GWSCSTYPFDSFHKSFRYKEHDVIIQ-PETGLLNLIDPVHEFKALTATEGATDKDIKMKFSNEVFRFASACMNCRTNGTIHFGVKDKPHG---EIVGVKLTEVDKSTFIDHFYTKME
360 ref|XP_043821246.1| 97.4% 57.4% ---GRPCSPYPFDNFHKSFWYKEHDIIIQ-PETGLLNLIDPVHEFKALTETEGATENDIKMKFSNEVFRFASACMNCRTNGTIHFGVKDKPHG---EIVGVKLGGEDKSAFVDHLNKKIE
361 ref|XP_008820771.1| 98.7% 57.4% ETEPMSCMPYPFDCFHASKHYIEHS-ILQVAETGPLNLIDPIHEFKALTNTETATEQDIKMKFSNEVFRFASACMNSRTNGTIHFGVKDKPHG---EIVGVEITR--KESFIEHFNVMMK
362 ref|XP_044534687.1| 97.0% 54.9% ---GRLCQTYPFDKFHESFRYRE-GAIMP-PETGPLNLIDPAHEYKALTNTERATEEDIMMKFSNEVFRFASACMNRRTNGTIHFGVKDSPHG---EIVGVKLTEGDKEKFVNHFYKKIE
363 ref|NP_001406829.1| 98.3% 55.9% KSEQMSCMPHPFNFAHDAKRYIEHS-ILRVAETGPLNLIDPVHEFKAFTNTETATQEQMKMKFSNETFRFAAACMNTRTNGTIHFGVKDKPHG---EIVGVQVTS--KDIFINHFNVMIT
364 ref|XP_032762875.1| 98.3% 55.9% KSEQMSCMPHPFNFAHDAKRYIEHS-ILRVAETGPLNLIDPVHEFKAFTNTETATQEQMKMKFSNETFRFAAACMNTRTNGTIHFGVKDKPHG---EIVGVQVTS--KDIFINHFNVMIT
365 ref|XP_003771993.1| 97.4% 57.0% ---GRPCSPYPFDNFHKSFRYKERDVIIQ-PETGSLNLIDPAHEFKALTETEGATRKDIYMKFSNEVFRFASACMNRRTNGTIHFGVKDKPHG---EIVGVKVIEEDKNKFVEHFYKKIE
366 ref|XP_068942244.1| 97.4% 56.2% ---GRPCSTYPFDKFHKSFRYKEHDVIIQ-PETGLLNLIDPVHEFKALTATEGATDKDIKMKFSNEVFRFASACMNCRTNGTIHFGVKDKPHG---EIVGVKLTKEDKSTFVEHFYKKIE
367 ref|XP_051856775.1| 97.4% 57.4% ---GRPCSPYPFDNFHKSFRYKEHDVIIQ-PETGSLNLIDPAHEFKALTETEGATRKDIYMKFSNEVFRFASACMNCRTNGTIHFGVKDKPHG---EIVGVKVTNEDKNKFTEHFYKKIE
368 ref|XP_038964512.2| 97.8% 55.5% KSEQMSCMPHPFNFAHDANRYIEHS-ILRVAETGPLNLIDPVHEFKAFTNTETA-QEQMKMKFSNETFRFAAACMNTRTNGTIHFGVKDKPHG---EIVGVQVTS--KDIFINHFNVMIT
369 ref|XP_027245644.1| 96.1% 55.9% KPEQMSCMPYPFDSFHDDRRYIERY-ILQVAETGPLNLIDPVHEFKDLTNTKT--EDQIKMKFSNEVFRFASACMNTRTNGTIHFGVKDKPHG---EIVGVKIPS--KDVFVDHFNKMIK
370 ref|XP_003496999.1| 96.1% 55.9% KPEQMSCMPYPFDSFHDDRRYIERY-ILQVAETGPLNLIDPVHEFKDLTNTKT--EDQIKMKFSNEVFRFASACMNTRTNGTIHFGVKDKPHG---EIVGVKIPS--KDVFVDHFNKMIK
consensus/100% ......C.sh.Fs..tt...Yh........sEoG..Nhh-sh+Eac.hht..........hKFopEhFcFsuAChN.+sNGTIahGl.spP......lhG.......pt.hhtHh..hh.
consensus/90% p....oChsYPFspFpss.RYh.p...LQ.PETGshNLIDPlHEFKshTNTptATEtDhKMKFSNEsFRFASACMNSRTNGTIHFGVpsKPHG...pIVGlphss..KcshIsHFNhMIp
consensus/80% p.-..TChsYPFDpFpsshRYh.ph.hLQ.PETGPLNLIDPlHEFKAhTNTcsATEcDlKMKFSNEVFRFASACMNSRTNGTIHFGVKDKPHG...pIVGVclTs..K-shIsHFNhMIc
consensus/70% p.E..TChsYPFDpFpsshRYh.ca.hLQ.PETGPLNLIDPIHEFKAhTNTEsATEcDlKMKFSNEVFRFASACMNSRTNGTIHFGVKDKPHG...cIVGVclTo..K-AhIDHFNlMI+

270 . . : . . . . 350 . . . 378

cov pid
1 ref|NP_001180236.1| 100.0% 100.0% KYFE-DHQVQQAKKCIREPRFVEVLLPNSTLSDRFVIEVDIIPQFSECQYDYFQIKMQNYN-NK--IWEQSKKFSLFVRDGTSSKDIT-----KNKVDFRAFKADFKTLA-ESRKAAEE-
2 ref|XP_001167273.1| 100.0% 99.1% KYFE-DHQVQQAKKCIREPRFVEVLLPNSTLSDRFVIEVDIIPQFSECQYDYFQIKMQNYN-NK--TWEQSKKFSLFVRDGTSSRDIT-----KNKVDFRAFKADFKTLA-ESRKAAEE-
3 ref|XP_004045789.1| 100.0% 98.7% KYFE-DHQVQQAKKCIREPRFVEVLLPNSTLSDRFVIEVDIIPQFSECQYDYFQIKMQNYN-NK--TWEQSKNFSLFVRDGTSSKDIT-----KNKVDFRAFKADFKTLA-ESRKAAEE-
4 ref|XP_055128666.1| 100.0% 97.4% KYFE-DHQVQQAKKCIREPRFVEVLLPNSTPSDRFVIEVDIIPQFSECQYDYFQIKMQNYN-NK--TWEQSKKFSLFVRDGPSSKDIM-----KNKVDFRAFKADFKTLA-ESRKAAEE-
5 ref|XP_054352670.1| 100.0% 96.5% KYFE-DHQVQQAKKCIREPRFVEVLLPNSTLSDRFVIEVDIIPQFSECQYDYFQIKMQNYN-NK--TWEQSKKFSLFVREGTSSKDIM-----KNKVDFRTFKADFKTLA-ESRKAAEE-
6 ref|XP_001090497.2| 100.0% 95.7% KYFE-DHQVQQAKKCIREPRFVEVLLPNSTLSDRFVIEVDIIPQFSECQYDYFQIKIQNYN-NK--TWEQSKKFSVFVRDGTSSKDIM-----KNKADFRTFKADFKTLA-ESRKAAEE-
7 ref|XP_011853659.1| 100.0% 95.7% KYFE-DHQVQQAKKCIREPRFVEVLLPNSTLSDRFVIEVDIIPQFSECQYDYFQIKIQNYN-NK--TWEQSKKFSVFVRDGTSSKDIM-----KNKADFRTFKADFKTLA-ESRKAAEE-
8 ref|XP_003896375.1| 100.0% 95.7% KYFE-DHQVQQAKKCIREPRFVEVLLPNSTLSDRFVIEVDIIPQFSECQYDYFQIKIQNYN-NK--TWEQSKKFSVFVRDGTSSKDIM-----KNKADFRTFKADFKTLA-ESRKAAEE-
9 ref|XP_011887444.1| 100.0% 95.7% KYFE-DHQVQQAKKCIREPRFVEVLLPNSTLSDRFVIEVDIIPQFSECQYDYFQIKIQNYN-NK--TWEQSKKFSVFVRDGTSSKDIM-----KNKADFRTFKADFKTLA-ESRKAAEE-
10 ref|XP_025235592.1| 100.0% 95.7% KYFE-DHQVQQAKKCIREPRFVEVLLPNSTLSDRFVIEVDIIPQFSECQYDYFQIKIQNYN-NK--TWEQSKKFSVFVRDGTSSKDIM-----KNKADFRTFKADFKTLA-ESRKAAEE-
11 ref|XP_005550278.3| 100.0% 95.2% KYFE-DHQVQQAKKCIREPRFVEVLLPNSTLSDRFVIEVDIIPQFSECQYDYFQIKIQNYN-NK--TWEQSKKFSVFVRDGTSSKDIM-----KNKADFRTFKTDFKTLA-ESRKAAEE-
12 ref|XP_002818257.3| 100.0% 96.1% KYFE-DHQVQQAKKCIREPRFVEVLLPNSTLSDRFVIEVDIILQFSECQYDYFQIKMQNYN-NK--TWEQSKKFSLFVREGTSSKDIM-----KNKVDFRTFKADFKTLA-ESRKAAEE-
13 ref|XP_007980346.2| 100.0% 94.8% KYFE-DHQVQQAKKCIREPRFVEVLLPNSTLSDRFVIEVDIIPQFSECQYDYFQIKIQNYN-NK--TWEQSKKFSVFLRDGTSSKDIM-----KNKADFRTFKADFKRLA-ESRKAAEE-
14 ref|XP_012502835.1| 100.0% 87.4% QYFE-DHRVQQAKKCIREPRFVEVLLPNSTLSDRFVIEVDVIPQYSECEDDYFQIKMQNYN-NK--TWEQSPKFSVFVRHGASSKDIM-----KNKADFREFKLDLKTLA-ESRKEAEE-
15 ref|XP_069318867.1| 100.0% 86.5% QYFE-DHQVQQAKKCIREPRFVEVLLPNSIPSDRFVIEVDVIPQYSECEHDYFQIKMQNCN-NK--TWEQSPKFSIFVRHGASSKDIM-----KNKADFREFKLDLKTLA-ESRKEAEE-
16 ref|XP_008570528.1| 100.0% 85.2% QYFE-DHQVQQAKKCIREPRFVEVLLPNSTLSDRCVIEVDVIPQYSECKHDYFQVKMQNYN-NK--TWEQSSKVSVFVRDGASTKDLM-----KNKVDFRAFKLDLKTLA-ESRKEAEE-
17 ref|XP_062957119.1| 100.0% 84.3% QYFE-DHQVQQAKKCIREPRFVEVLMPNSTLSDKCVIEVDVIPQYSECKHDYFQVKMQNYN-NK--TWEQSSKFSVFVRDGASTKDLM-----KNKVDFRAFKLDLKTLA-ESRKEAEE-
18 ref|XP_014643658.1| 100.0% 83.9% QYFE-DHQVQKAKKCIREPRFVEVLLPNSTPSDRFVIEVDVIPKYSECEHDYFQIKMQNYN-NK--TWTQSSKFSVFVRDGASSKDIV-----KNNADFKSFKLDLKTLA-ESRREAEE-
19 ref|XP_006155597.1| 100.0% 83.9% QYFE-DHQVQQAKKCIREPRFVEVLLPNSTLSDRFVIEVDVIPQYSECAYDYFQIKVQNYK-NK--MWEQSPKFSVFVRDGASTKDIM-----KNKTDFRAFKLDLKPLA-ESRKEAEE-
20 ref|XP_058381579.1| 100.0% 83.5% QYFE-DHQVQKAKKCIREPRFVEVLLPNSTPSDRFVIEVDVIPKYSECEHDYFQIKMQNYN-NK--TWTQSSKFSVFVRDGASSKDIV-----KNNADFKSFKLDLKTLA-ESRREAEE-
21 ref|XP_058381571.1| 100.0% 83.5% QYFE-DHQVQKAKKCIREPRFVEVLLPNSTPSDRFVIEVDVIPKYSECEHDYFQIKMQNYN-NK--TWTQSSKFSVFVRDGASSKDIV-----KNNADFKSFKLDLKTLA-ESRREAEE-
22 ref|XP_045420643.1| 100.0% 83.9% QYFE-DHQVQQAKKCIREPRFVEVLLPNSIPSDRFVIEVDVIPQYSECEHDYFQIKMQNCN-NK--TWEQSPKYSVFVRHGASSKDIT-----KKEADFREFKLDLKTLA-ESRKEAEE-
23 ref|XP_070280365.1| 100.0% 83.0% QYFE-DHQVQKAKKCIREPRFVEVLLPNSTPSDRFVIEVDVIPKYSECEHDYFQIKMQNCN-NK--VWEQRKHFSVFVRDGASSKDIM-----KNKVDFKIFQLGLEALA-EHRKEAEE-
24 ref|XP_006108753.1| 100.0% 83.0% QYFE-DHQVQKAKKCIREPRFVEVLLPNSTPSDRFVIEVDVIPKYSECEHDYFQIKMQNCN-NK--VWEQRKHFSVFVRDGASSKDIM-----KNKVDFKIFQLGLEALA-EHRKEAEE-
25 ref|XP_014403134.1| 100.0% 82.6% QYFE-DHQVQKAKKCIREPRFVEVLLPNSTPSDRFVIEVDVIPKYSECGHDYFQIKMQNCN-NK--VWEQRKHFSVFVRDGASSKDIM-----KNKVDFKTFQLGLEAMA-EHRKEAEE-
26 ref|XP_036184081.1| 100.0% 82.6% QYFE-DHHVQKAKKCIREPRFVEVLLPNSTPSDRFVIEVDVIPKYSECGHDYFQIKMQNCN-NK--VWEQRKHFSVFVRDGASSKDIM-----KNKVDFKIFQLGLEALA-EHRKEAEE-
27 ref|XP_014403133.1| 100.0% 82.6% QYFE-DHQVQKAKKCIREPRFVEVLLPNSTPSDRFVIEVDVIPKYSECGHDYFQIKMQNCN-NK--VWEQRKHFSVFVRDGASSKDIM-----KNKVDFKTFQLGLEAMA-EHRKEAEE-
28 ref|XP_036184079.1| 100.0% 82.6% QYFE-DHHVQKAKKCIREPRFVEVLLPNSTPSDRFVIEVDVIPKYSECGHDYFQIKMQNCN-NK--VWEQRKHFSVFVRDGASSKDIM-----KNKVDFKIFQLGLEALA-EHRKEAEE-
29 ref|XP_036184078.1| 100.0% 82.6% QYFE-DHHVQKAKKCIREPRFVEVLLPNSTPSDRFVIEVDVIPKYSECGHDYFQIKMQNCN-NK--VWEQRKHFSVFVRDGASSKDIM-----KNKVDFKIFQLGLEALA-EHRKEAEE-
30 ref|XP_005876148.1| 100.0% 82.6% QYFE-DHQVQKAKKCIREPRFVEVLLPNSTPSDRFVIEVDVIPKYSECGHDYFQIKMQNCN-NK--VWEQRKHFSVFVRDGASSKDIM-----KNKVDFKTFQLGLEAMA-EHRKEAEE-
31 ref|XP_036184077.1| 100.0% 82.6% QYFE-DHHVQKAKKCIREPRFVEVLLPNSTPSDRFVIEVDVIPKYSECGHDYFQIKMQNCN-NK--VWEQRKHFSVFVRDGASSKDIM-----KNKVDFKIFQLGLEALA-EHRKEAEE-
32 ref|XP_059568302.1| 100.0% 82.6% QYFE-DHHVQKAKKCIREPRFVEVLLPNSTPSDRFVIEVDVIPKYSECGHDYFQIKMQNCN-NK--VWEQRKHFSVFVRDGASSKDIM-----KNKVDFKIFQLGLEALA-EHRKEAEE-
33 ref|XP_036285323.1| 100.0% 82.6% QYFE-NHQVQKAKKCIREPRFVEVLLPNSTPSDRFVIEVDVIPKYSECEHDYFQIKMQNYN-NQ--VWEPRKDFSVFVRDGASSKDIM-----KKKVDFEMFKLDLKALA-EHRKEAEE-
34 ref|XP_015413823.1| 100.0% 82.6% QYFE-DHQVQKAKKCIREPRFVEVLLPNSTPSDRFVIEVDVIPKYSECGHDYFQIKMQNCN-NK--VWEQRKHFSVFVRDGASSKDIM-----KNKVDFKIFKLGLETLA-EHRKEAEE-
35 ref|XP_036184076.1| 100.0% 82.6% QYFE-DHHVQKAKKCIREPRFVEVLLPNSTPSDRFVIEVDVIPKYSECGHDYFQIKMQNCN-NK--VWEQRKHFSVFVRDGASSKDIM-----KNKVDFKIFQLGLEALA-EHRKEAEE-
36 ref|XP_006779271.1| 100.0% 82.6% QYFE-DHQVQKAKKCIREPRFVEVLLPNSTPSDRFVIEVDVIPKYSECGHDYFQIKMQNCN-NK--VWEQRKHFSVFVRDGASSKDIM-----KNKVDFKIFKLGLETLA-EHRKEAEE-
37 ref|XP_004676740.2| 100.0% 81.3% KYFD-DHQVQKAKKCIREPRFVEVLLPNSIPSDRFVIEVDVIPKYSECEYDYFQIKMQNYN-NK--TWEQSPKPSVYVRDGASSKDFM-----RNNADFRAFKLDIKALA-DSRKEAEE-
38 ref|XP_012576483.1| 100.0% 81.3% KYFD-DHQVQKAKKCIREPRFVEVLLPNSIPSDRFVIEVDVIPKYSECEYDYFQIKMQNYN-NK--TWEQSPKPSVYVRDGASSKDFM-----RNNADFRAFKLDIKALA-DSRKEAEE-
39 ref|XP_059964408.1| 100.0% 80.9% QYFE-DHQVQKAKSCIREPRFVEVLMPNSTPSDRFVIEVDVVPKYSECEHDYFQIKMQNYS-KK--TWTQSPKFSVFVRDGASSKDIM-----KNNAYFTSFKLDLKRLA-ESRKEAEE-
40 ref|XP_014698446.3| 100.0% 80.4% QYFE-DHQVQKAKKCIREPRFVEVLLPNSTPSDRFVIEVDVIPKYSECEDDYFRIKIQNCD-NN--TWKQSSKFSIFVRDGASSKDIM-----KNNADFKEFKLDLKTLA-EARKEAEE-
41 ref|XP_046526610.1| 100.0% 80.4% QYFE-DHQVQKAKKCIREPRFVEVLLPNSTPSDRFVIEVDVIPKYSECEDDYFRIKIQNCD-NN--TWKQSSKFSIFVRDGASSKDIM-----KNNADFKEFKLDLKTLA-EARKEAEE-
42 ref|XP_046526611.1| 100.0% 80.4% QYFE-DHQVQKAKKCIREPRFVEVLLPNSTPSDRFVIEVDVIPKYSECEDDYFRIKIQNCD-NN--TWKQSSKFSIFVRDGASSKDIM-----KNNADFKEFKLDLKTLA-EARKEAEE-
43 ref|XP_028012848.2| 100.0% 80.9% QYFE-DHQVQKAKKCIREPRFVEVLLPNSTPSDRFVIEVDVIPKYSECEHDYFQIKMQNCN-NQ--VWEQRKHFSVFVRDGASSKDIM-----KKKVDFEMFKLGLKALA-EHRKEAEE-
44 ref|XP_019483139.1| 99.6% 80.1% QYFE-DHQVQKAKNCIREPRFVEVLLPNSIPSDRFVIEVDVIPKYSECEHDYFQIKMQNRNNKK---WEQGSNFSVFVRDGASSKDIR-----KNNVDFAAFKSNLKTRA-QSRKDAEE-
45 ref|XP_053409644.1| 99.6% 81.3% QYFE-DHQIQKAKKCIREPRFVEVLLPNSNLSDRFVIEVDVIPQYSECECDYFQIKMQIYN--K--TWEQSSKFSVFVRVGTSTKNII-----KNKTDFIEFKSDLKKLA-EARKAAEE-
46 ref|NP_001243854.1| 100.0% 80.0% QYFE-DHQVQKAKKCIREPRFVEVLLPNSTPSDRFVIEVDVIPKYSECEDDYFRIKIQNCD-NN--TWKQSSKFSIFVRDGASSKDIM-----KNNADFKEFKLDLKTLA-EARKEAEE-
47 ref|XP_057589207.1| 100.0% 79.6% QYFE-DHQVQKAKNCIREPRFVEVLLPNSSLSNRFVIEVDVVPKYSECEYDYFQIKMQNCS-NK--KWTQNPKFSVFIRDGASSKDIV-----KNNLDFKEFKLDLKKLS-ESRKEAEE-
48 ref|XP_040116208.1| 100.0% 80.0% QYFE-DHQVQKAKNCIREPRFVEVLLPNSTLSDRFVIEVDVVPKYSECEVDYFQIKMQNYS-NT--IWKQSPKFSVFVRDGASSKDIM-----KSNADFKAFKLDLKKLA-ESRKEAEE-
49 ref|XP_024622799.1| 100.0% 81.3% KYFE-DHQVQKAKNCIREPRFVEVLMPNSIPSDRFVIEVDVVPKYSECEHDYFQIKMQNCS-NK--TWTQSPKFSVFVRDGTSSKDIM-----KNNTYFTSFKLDLKRLA-ESRKEAEE-
50 ref|XP_024622798.1| 100.0% 81.3% KYFE-DHQVQKAKNCIREPRFVEVLMPNSIPSDRFVIEVDVVPKYSECEHDYFQIKMQNCS-NK--TWTQSPKFSVFVRDGTSSKDIM-----KNNTYFTSFKLDLKRLA-ESRKEAEE-
51 ref|XP_010977976.2| 100.0% 79.6% QYFE-DHQVQKAKNCIREPRFVEVLLPNSTPSDRFVIEVDVVPKYSECEHEYFQIKIQNCS-NE--TWKQSPNFSVFVRDGASSKDIM-----KNKADFKAFKLDLKRLG-EARKEAEE-
52 ref|XP_024622796.1| 100.0% 81.3% KYFE-DHQVQKAKNCIREPRFVEVLMPNSIPSDRFVIEVDVVPKYSECEHDYFQIKMQNCS-NK--TWTQSPKFSVFVRDGTSSKDIM-----KNNTYFTSFKLDLKRLA-ESRKEAEE-
53 ref|XP_042104307.1| 100.0% 80.0% QYFE-DHQVQKAKNCIREPRFVEVLLPNSTLSDRFVIEVDVVPKYSECEVDYFQIKMQNYS-NT--IWKPSSKFSVFVRDGASSKDIM-----KSNADFKAFKLDLKRLA-ESRKEAEE-
54 ref|XP_006207699.1| 100.0% 79.6% QYFE-DHQVQKAKNCIREPRFVEVLLPNSTPSDRFVIEVDVVPKYSECEHEYFQIKIQNCS-NE--TWKQSPNFSVFVRDGASSKDIM-----KNKADFKAFKLDLKRLG-EARKEAEE-
55 ref|XP_043318526.1| 100.0% 80.0% QYFE-DHQVQKAKNCIREPRFVEVLLPNSSLSDRFVIEVDVVPKYSECEVDYFQIKMQNYS-NT--IWKQSPKFSVFVRDGASSKDIM-----KSNADFKAFKLDLKKLA-ESRKEAEE-
56 ref|XP_020751092.2| 100.0% 80.0% QYFE-DHQVQKAKNCIREPRFVEVLLPNSSLSDRFVIEVDVVPKYSECEVDYFQIKMQNYS-NT--IWKQSPKFSVFVRDGASSKDIM-----KSNADFKAFKLDLKKLA-ESRKEAEE-
57 ref|XP_070327721.1| 100.0% 80.0% QYFE-DHQVQKAKNCIREPRFVEVLLPNSSLSDRFVIEVDVVPKYSECEVDYFQIKMQNYS-NT--IWKQSPKFSVFVRDGASSKDIM-----KSNADFKAFKLDLKKLA-ESRKEAEE-
58 ref|XP_022441612.1| 100.0% 80.4% KYFE-DHQVQKAKNCIREPRFVEVLMPNSIPSDRFVIEVDVVPKYSECEHDYFQIKMQNFS-KK--TWTQSPKFSVFVRDGASSKDIM-----KNNAYFTSFKLDLKRLA-ESRKEAEE-
59 ref|XP_029089619.1| 100.0% 80.4% KYFE-DHQVQKAKNCIREPRFVEVLMPNSIPSDRFVIEVDVVPKYSECEHDYFQIKMQNFS-KK--TWTQSPKFSVFVRDGASSKDIM-----KNNAYFTSFKLDLKRLA-ESRKEAEE-
60 ref|XP_022441604.1| 100.0% 80.4% KYFE-DHQVQKAKNCIREPRFVEVLMPNSIPSDRFVIEVDVVPKYSECEHDYFQIKMQNFS-KK--TWTQSPKFSVFVRDGASSKDIM-----KNNAYFTSFKLDLKRLA-ESRKEAEE-
61 ref|XP_043318525.1| 100.0% 80.0% QYFE-DHQVQKAKNCIREPRFVEVLLPNSSLSDRFVIEVDVVPKYSECEVDYFQIKMQNYS-NT--IWKQSPKFSVFVRDGASSKDIM-----KSNADFKAFKLDLKKLA-ESRKEAEE-
62 ref|XP_070327713.1| 100.0% 80.0% QYFE-DHQVQKAKNCIREPRFVEVLLPNSSLSDRFVIEVDVVPKYSECEVDYFQIKMQNYS-NT--IWKQSPKFSVFVRDGASSKDIM-----KSNADFKAFKLDLKKLA-ESRKEAEE-
63 ref|XP_061020880.1| 100.0% 79.6% QYFE-DHQVQKAKNCIREPRFVEVLLPNSSLSDRFVIEVDVVPKYSECEVDYFQIKMQNYS-NT--IWKQSPKFSVFVRDGASSKDIM-----KSNADFKAFKLDLKKLA-ESRKEAEE-
64 ref|XP_022441597.1| 100.0% 80.4% KYFE-DHQVQKAKNCIREPRFVEVLMPNSIPSDRFVIEVDVVPKYSECEHDYFQIKMQNFS-KK--TWTQSPKFSVFVRDGASSKDIM-----KNNAYFTSFKLDLKRLA-ESRKEAEE-
65 ref|XP_069443508.1| 100.0% 79.6% QYFE-DHQVQKAKNCIREPRFVEVLLPNSSLSDRFVIEVDVVPKYSECEVDYFQIKMQNYS-NT--IWKPSSKFSVFVRDGASSKDIM-----KSNADFKAFKLDLKRLA-ESRKEAEE-
66 ref|XP_010971009.1| 100.0% 79.1% QYFE-DHQVQKAKNCIREPRFVEVLLPNSTPSDRFVIEVDVVPKYSECEHEYFQIKIQNCS-NE--TWKQSPNFSVFVRDGASSKDIM-----KNKADFKAFKLDLKRLG-EARKEAEE-
67 ref|XP_067602509.1| 100.0% 80.4% KYFE-DHQVQKAKNCIREPRFVEVLMPNSTPSDRFVIEVDVVPKYSECEHDYFQIKMQNCS-KK--TWTQSPKFSVFVRDGASTKDIM-----KNNAYFTSFKLDLKRLA-ECRKEAEE-
68 ref|XP_005667662.1| 100.0% 78.7% QYFE-DHQVQKAKNCIREPRFVEVLLPNSIPSDRFVIEVDVVPKYSECEQEYFQIKMQNYS-NK--TWKQSPKFSVFMRDGASSKDVM-----KNNVEFKTFKLDLKKLA-EARKEAEE-
69 ref|XP_005678975.2| 100.0% 79.6% QYFE-DHQVQKAKNCIREPRFVEVLLPNSSLSDRFVIEVDVVPKYSECEVDYFQIKMQNYS-NT--IWKPSPKFSVFVRDGASSKDIM-----KSNADFKAFKLDLKKLA-ESRKEAEE-
70 ref|XP_023363488.1| 99.6% 80.4% QYFE-DHQIQKAKKCIREPRFVEVLLPNSNLSDRFVIEVDVIPQYSECECDYFQIKMQIYN--K--TWEQSPKFSVFVRDGTSTKNII-----KNKTDFIEFKTDLKKLA-EARKAAEE-
71 ref|XP_020958041.1| 100.0% 78.7% QYFE-DHQVQKAKNCIREPRFVEVLLPNSIPSDRFVIEVDVVPKYSECEQEYFQIKMQNYS-NK--TWKQSPKFSVFMRDGASSKDVM-----KNNVEFKTFKLDLKKLA-EARKEAEE-
72 ref|XP_007461947.1| 100.0% 80.4% KYFE-DHQVQKAKNCIREPRFVEVLMPNSTPSDRFVIEVDVVPKYSECEHDYFQIKMQNCS-KK--TWTQSPKFSVFVRDGASSKDMM-----KNNAYFTSFKLDLKRLA-ESRKEAEE-
73 ref|XP_065794903.1| 100.0% 79.6% QYFE-DHQVQKAKNCIREPRFVEVLLPNTSLSDRFVIEVDVVPKYSECEVDYFQIKMQNYS-NK--IWKQSPKFSVFVRDGASSKDIM-----KSNADFKAFKLDLKKLA-ESRKEAEE-
74 ref|XP_065740132.1| 100.0% 80.9% KYFE-DHQVQKAKNCIREPRFVEVLMPNSIPSDRFVIEVDVVPKYSECEHDYFQIKMQNCS-NK--TWTQSPKFSVFVRDGASSKDIM-----KNNTYFTSFKLDLKRLA-ESRKEAEE-
75 ref|XP_032499074.1| 100.0% 80.9% KYFE-DHQVQKAKNCIREPRFVEVLMPNSIPSDRFVIEVDVVPKYSECEHDYFQIKMQNCS-NK--TWTQSPKFSVFVRDGASSKDIM-----KNNTYFTSFKLDLKRLA-ESRKEAEE-
76 ref|XP_023985582.1| 100.0% 80.4% QYFE-DHQVQIAKNCIREPRFVEVLMPNSTPSDRFVIEVDVVPKYSECEHDYFQIKMQNYN-KK--TWTQSPKFSVFVRDGASSKDIM-----KNNAYFTSFKLDLKRLA-ESRKEAEE-
77 ref|XP_032499071.1| 100.0% 80.9% KYFE-DHQVQKAKNCIREPRFVEVLMPNSIPSDRFVIEVDVVPKYSECEHDYFQIKMQNCS-NK--TWTQSPKFSVFVRDGASSKDIM-----KNNTYFTSFKLDLKRLA-ESRKEAEE-

```
 78 ref|XP_032499073.1| 100.0%  80.9%   KYFE-DHQVQKAKNCIREPRFVEVLMPNSIPSDRFVIEVDVVPKYSECEHDYFQIKMQNCS-NK--TWTQSPKFSVFVRDGASSKDIM-----KNNTYFTSFKLDLKRLA-ESRKEAEE-
 79 ref|XP_052495196.1| 100.0%  79.6%   QYFE-DHQVQKAKNCIREPRFVEVLLPNITLSDRFVIEVDVVPKYSECEVDYFQIKMQNYS-NT--IWKPSPKFSVFVRDGASSKDIM-----KSNADFKAFKLDLKRLA-ESRKEAEE-
 80 ref|XP_060013026.1| 100.0%  80.0%   KYFE-DHQVQKAKNCIREPRFVEVLMPNSTPSDRFVIEVDVVPKYSECEHDYFQIKMQNCS-KK--TWTQSPKFSVFVRDGASTKDIM-----KNNAYFTSFKLDLKRLA-ECRKEAEE-
 81 ref|XP_059876504.1| 100.0%  80.0%   KYFE-DHQVQKAKNCIREPRFVEVLMPNSTPSDRFVIEVDVVPKYSECEHDYFQIKMQNCS-KK--TWTQSPKFSVFVRDGASTKDIM-----KNNAYFTSFKLDLKRLA-ECRKEAEE-
 82 ref|XP_012387005.2| 100.0%  80.0%   KYFE-DHQVQKAKNCIREPRFVEVLMPNSTPSDRFVIEVDVVPKYSECEHDYFQIKMQNCS-KK--TWTQSPKFSVFVRDGASTKDIM-----KNNAYFTSFKLDLKRLA-ESRKEAEE-
 83 ref|XP_026958261.1| 100.0%  80.0%   KYFE-DHQVQKAKNCIREPRFVEVLMPNSTPSDRFVIEVDVVPKYSECEHDYFQIKMQNCS-KK--TWTQSPKFSVFVRDGASTKDIM-----KNNAYFTSFKLDLKRLA-ECRKEAEE-
 84 ref|XP_049571082.1| 100.0%  80.0%   KYFE-DHQVQKAKNCIREPRFVEVLMPNSTPSDRFVIEVDVVPKYSECEHDYFQIKMQNCS-KK--TWTQSPKFSVFVRDGASTKDIM-----KNNAYFTSFKLDLKRLA-ESRKEAEE-
 85 ref|XP_030713128.1| 100.0%  80.0%   KYFE-DHQVQKAKNCIREPRFVEVLMPNSTPSDRFVIEVDVVPKYSECEHDYFQIKMQNCS-KK--TWTQSPKFSVFVRDGASTKDIM-----KNNAYFTSFKLDLKRLA-ECRKEAEE-
 86 ref|XP_070643590.1| 100.0%  79.1%   QYFE-DHQVQKAKNCIREPRFVEVLLPNSTLSDRFVIEVDVVPKYSECEVDYFQIKMQNCS-NT--IWKQSPNFSVFVRDGASSKDIM-----KSNMDFKAFKLDLKRLA-ESRKEAEE-
 87 ref|XP_068406760.1| 100.0%  80.4%   QYFE-DHQVQKAKNCIREPRFVEVLMPNSTPSDRFVIEVDVVPKHSECEHDYFQIKMQNCS-KK--TWTQSPKFSVFVRDGASSKDIM-----KNNAYFTSFKLDLKRLA-ESRKEAEE-
 88 ref|XP_060160576.1| 100.0%  80.0%   KYFE-DHQVQKAKNCIREPRFVEVLMPNSTPSDRFVIEVDVVPKYSECEHDYFQIKMQNCS-KK--TWTQSPKFSVFVRDGASTKDIM-----KNNAYFTSFKLDLKRLA-ECRKEAEE-
 89 ref|XP_005902867.1| 100.0%  79.1%   QYFE-DHQVQKAKNCIREPRFVEVLLPNSTLSDRFVIEVDVVPKYSECEVDYFQIKMQNCS-NT--IWKQSPNFSVFVRDGASSKDIM-----KSNMDFKAFKLDLKRLA-ESRKEAEE-
 90 ref|NP_001192710.3| 100.0%  79.1%   QYFE-DHQVQKAKNCIREPRFVEVLLPNSTLSDRFVIEVDVVPKYSECEVDYFQIKMQNCS-NT--IWKQSPNFSVFVRDGASSKDIM-----KSNMDFKAFKLDLKRLA-ESRKEAEE-
 91 ref|XP_007195831.2| 100.0%  80.4%   QYFE-DHQVQKAKNCIREPRFVEVLMPNSTPSDRFVIEVDVVPKHSECEHDYFQIKMQNCS-KK--TWTQSPKFSVFVRDGASSKDIM-----KNNAYFTSFKLDLKRLA-ESRKEAEE-
 92 ref|XP_036720276.1| 100.0%  80.4%   QYFE-DHQVQKAKNCIREPRFVEVLMPNSTPSDRFVIEVDVVPKHSECEHDYFQIKMQNCS-KK--TWTQSPKFSVFVRDGASSKDIM-----KNNAYFTSFKLDLKRLA-ESRKEAEE-
 93 ref|XP_007195832.2| 100.0%  80.4%   QYFE-DHQVQKAKNCIREPRFVEVLMPNSTPSDRFVIEVDVVPKHSECEHDYFQIKMQNCS-KK--TWTQSPKFSVFVRDGASSKDIM-----KNNAYFTSFKLDLKRLA-ESRKEAEE-
 94 ref|XP_036720275.1| 100.0%  80.4%   QYFE-DHQVQKAKNCIREPRFVEVLMPNSTPSDRFVIEVDVVPKHSECEHDYFQIKMQNCS-KK--TWTQSPKFSVFVRDGASSKDIM-----KNNAYFTSFKLDLKRLA-ESRKEAEE-
 95 ref|XP_016016799.2| 100.0%  79.7%   QYFE-DHQVSKAKNCIREPRFVEVLQQNSIPSERFVIEVDVIPKHSECEHDYFQIKMQNYN-NK--IWEQRPKFSVFVRDGASSKDII-----KNNADFRAFKLDLKRLA-ESRKEAEAE
 96 ref|XP_061054544.1| 100.0%  80.4%   QYFE-DHQVQKAKNCIREPRFVEVLMPNSTPSDRFVIEVDVVPKHSECEHDYFQIKMQNCS-KK--TWTQSPKFSVFVRDGASSKDIM-----KNNAYFTSFKLDLKKLA-ESRKEAEE-
 97 ref|XP_059789816.1| 100.0%  79.6%   QYFE-DHQVQKAKNCIREPRFVEVLMPNSTPSDRFVIEVDVVPKHSECEHDYFQIKMQNCS-KK--TWTQSPKFSVFVRDGASSKDIV-----KNNAYFTSFKLDLKRLA-ESRKEAEE-
 98 ref|XP_004329146.1| 100.0%  79.6%   KYFE-DHQVQKAKNCIREPRFVEVLMPNSTPSDRFVIEVDVVPKYSECEHDYFQIKMQNCS-KK--TWTQSPKFSVFVRDGARTKDIM-----KNNAYFTSFKLDLKRLA-ECRKEAEE-
 99 ref|XP_045684325.1| 100.0%  79.6%   QYFE-DHQVQIAKKCIREPRFVEVLLPNSTPSDKFVIEVDVIPKYSECGHDYFQIKMQNCN-NK--KWEQRTKFSVFVRDGASTKDIM-----KNNAEFKDFTLGLKTLA-KSRQEAEE-
100 ref|XP_068829026.1| 100.0%  78.7%   QYFE-DHQVQKAKNCIREPRFVEVLLPNITLSDRFVIEVDVVPKYSECEVDYFQIKMQNYS-NT--IWKPSPKFSVFVRDGASSKDVM-----KSNADFKAFKLDLKRLA-ESRKEAEE-
101 ref|XP_019782959.1| 100.0%  79.6%   KYFE-DHQVQKAKNCIREPRFVEVLMPNSTPSDRFVIEVDVVPKYSECEHDYFQIKMQNCS-KK--TWTQSPKFSVFVRDGARTKDIM-----KNNAYFTSFKLDLKRLA-ECRKEAEE-
102 ref|XP_045041219.1| 100.0%  80.4%   QYFE-DHQVQIAKKCIREPRFVEVLLPNSTPSDKFVIEVDVIPKYSECGDDYFQIKMQNCN-NK--IWEPRTKFSVFVRDGASTKDIM-----KNNAEFKEFTLGLKTLA-KSRNEAEE-
103 ref|XP_037363830.1| 100.0%  79.1%   KYFE-DHQVQKAKKCIREPRFVEVLLPNSIPSDKFVIEVDVIPKYSECEYDYFQIKMQNCN-NK--TWEQSPKSSFYIRDGTSSKDLM-----KNNADLRAFKLHIKELA-ESRKEAEE-
104 ref|XP_007522554.1|  99.1%  78.9%   QYFE-HHQVQKAKKCIREPRFVEVLLPNSTPSERYVIEVDVIPKYSECERDYFQIKMQNYD-NK--IWEQSSKVSVFVRDGASSKDII-----KKNSDFKEFKLDLKVLA-ESRKEAEE-
105 ref|XP_060052209.1|  99.1%  78.9%   QYFE-HHQVQKAKKCIREPRFVEVLLPNSTPSERYVIEVDVIPKYSECERDYFQIKMQNYD-NK--IWEQSSKVSVFVRDGASSKDII-----KKNSDFKEFKLDLKVLA-ESRKEAEE-
106 ref|XP_047608878.1| 100.0%  77.8%   QYFE-DHQVQKAKNCIREPRFVEVLQPNSIPSDRFVIEVDVVPKYSECEQEYFQIKMQNYS-NK--TWKQSPKFSVFMRDGASSKDVM-----KNNVEFTTFKLDLKKLA-EARKEAEE-
107 ref|XP_048195986.1| 100.0%  78.7%   QYFE-SHQVQQAKKCIREPRFVEVLLPNGTLSDRFVIEVDVIPQYSESGQDYFQIKMQNYS-NN--TWEQSSKYSVFVRDGPNCKNIF-----KNKAAFQAFKLDLKTMS-ESRKEAEE-
108 ref|XP_039734026.1| 100.0%  79.3%   QYFE-DHQVPKAKNCIREPRFVEVLQQNSIPSDRFVIEVDVIPKHSECEHDYFQIKMQNYN-NK--IWEQRPKFSVFVRDGASSKDII-----KNNADFRAFKLDLKRLA-DARKEAEAE
109 ref|XP_028381693.1| 100.0%  79.1%   QYFE-DHQVQIAKKCIREPRFVEVLLPNSTPSDKFVIEVDVIPKYSECGHDYFQIKMQNCN-NK--KWERRTKFSVFVRDGASTKDIM-----KNNAEFKDFKLGLKTLA-KSRQEAEE-
110 ref|XP_016074271.1| 100.0%  78.3%   QYFE-DHQVQKAKKCIREPRFVEVLLPNSVPSDRFVIEVDVIPQYSECGHDYFQIKMQNCN-NK--IWEQREKFSVFVRDGASSKDIM-----KNNADFKIFKLDLEALA-GRRKEAEE-
111 ref|XP_036992384.2| 100.0%  79.1%   QYFE-DHQVQIAKKCIREPRFVEVLLPNSTPSDKFVIEVDVIPKYSECGHDYFQIKLQNCN-NK--IWEKRTKFSVFVRDGASTKDII-----KNNAEFKDFKLGLKTLA-KSRKEAEE-
112 ref|XP_006911959.1| 100.0%  78.4%   QYFE-DHQVPKAKNCIREPRFVEVLQQNSIPSDRFVIEVDVIPKHSECEHDYFQIKMQNYN-NK--IWEQRPKFSVFVRDGANSKDII-----KNNAEFRAFKLDLKRLA-DARKEAEAE
113 ref|XP_011361926.1| 100.0%  78.4%   QYFE-DHQVPKAKNCIREPRFVEVLQQNSIPSDRFVIEVDVIPKHSECEHDYFQIKMQNYK-DK--IWEQRPKFSVFVRDGASSKDII-----KNNADFRAFKLDLKRLA-DARKEAEAE
114 ref|XP_020016740.1| 100.0%  78.7%   QYFE-AHQVQQAKKCIREPRFVEVLLPNSTLSDRFVVEVDVIPQYSECEHDYFQIKMQNYS-NS--KWEQSSKYSVFVRDGPKTKDII-----KTKADFKAFKLDLKTVA-ESRKEAEE-
115 ref|XP_020016739.1| 100.0%  78.7%   QYFE-AHQVQQAKKCIREPRFVEVLLPNSTLSDRFVVEVDVIPQYSECEHDYFQIKMQNYS-NS--KWEQSSKYSVFVRDGPKTKDII-----KTKADFKAFKLDLKTVA-ESRKEAEE-
116 ref|XP_004862414.1|  98.7%  77.1%   QYFE-AHQVQQAKKCIREPRFVEVLLPNSTVSDRFVVEVDVVPQFSECKHDYFQIKMQNCN-NK--AWQQSSKYSVFVRDGPNSKNIL-----KNKADFKAFQLDLKTVA-VSRKEAEE-
117 ref|XP_012934202.1|  98.7%  77.1%   QYFE-AHQVQQAKKCIREPRFVEVLLPNSTVSDRFVVEVDVVPQFSECKHDYFQIKMQNCN-NK--AWQQSSKYSVFVRDGPNSKNIL-----KNKADFKAFQLDLKTVA-VSRKEAEE-
118 ref|XP_054420471.1|  99.6%  79.6%   QYFE-DHQAQIAKKCIREPRFVEVLLPNSIPSDKFVIEVDVIPKYSECGHDYFQIKRQNCN-NK--IWEQSTKFSVFVRDGTSTKNII-----NSNAVFKEFKLGLKTLA-ESRKEAEE-
119 ref|XP_066210460.1| 100.0%  77.8%   QYFE-DHQVQQAKKCIREPRFVEVLLPNSTLSDRFVIEVDVIPKYSECGHDYFQIKMQICN-NN--IWEQKPNFSVFVRDGASSKDIM-----KKNTDFKAFKLDLKTMA-ESRKEAET-
120 ref|XP_013013899.2|  98.7%  75.8%   QYFE-AHQVQQAKKCIREPRFVEVLLPNSTVSDRFVVEVDVVPQYSECKHDYFRITMQNYN-HE--TWQPSSKYSVFIRDGPNTKNII-----KNKVDFEAFQLDLKSVA-TSRKEAEE-
121 ref|XP_066095023.1| 100.0%  77.4%   QYFE-DHQVQQAKKCIREPRFVEVLLPNSTLSDRFVIEVDVIPKYSECGHDYFQIKMQICN-NN--TWEQKPNFSVFVRDGASSKDIM-----KKNTDFKAFKLDLKTMA-ESRKEAET-
122 ref|XP_004646297.1|  98.7%  74.9%   EYFE-AHQVQQAKKCIREPRFVEVLLPNSAVSDRFVVEVDVVPQYSECKYDYFQVKMQNYS-NK--TWQQSSKFSLFVRDGPNTKNII-----KNQVEFKTFQLDLKAVA-TARKEAEE-
123 ref|XP_059113322.1| 100.0%  75.8%   QYFE-VHQVQQAKKCIREPRFVEVLLPNSTPSNKFVIEVDVIPQHSECREDYFQIKMQHLK-NQ--TWQQSSKYSVFVRDGPQSKDII-----GNKADFKAFKLDLKAVA-DSRKEAET-
124 ref|XP_032944686.1|  98.7%  77.8%   KYFE-NHQVQKAKNCIREPRFVEVLLPNSIPSDRFVIEVDVIPKYSECEDDYFQIKMQNYN-QK---WEQGSKFSVFVRDGASSTDIR-----KKSADFKEFKLNLKTLA-QSRKEAEE-
125 ref|XP_010838083.1|  99.1%  77.0%   QYFE-DHQVQKAKNCIREPRFVEVLLPNSTLSDRFVIEVDVVPKYSECEVDYFQIKMQNCS-NT--IWKQSPNFSVFVRDGASSKDIM-----KSNMDFKAFKLDLKRLA-ESRKEAEE-
126 ref|XP_052575709.1| 100.0%  75.3%   QYFE-VHQVQQAKKCIREPRFVEVLLPNSTPSNKFVIEVDVIPQHSECREDYFQIKMQHLK-NQ--TWQQSSKYSVFVRDGPQSKDII-----GNKADFKAFKLDLKAVA-DSRKEAET-
127 ref|XP_019585265.1|  98.7%  77.4%   KYFE-DHQVQKAKNCIREPRFVEVLLPNSIPSDRFVIEVDVIPKYSECEDDYFQIKMQNYN-KK---WEQGSKFSVFVRNGASSTDIM-----KKSADFKEFKLNLKTLA-QSRKEAEE-
128 ref|XP_036037353.1| 100.0%  74.9%   QYFE-VHQVQQAKKCIREPRFVEVLLPNSTPSNKFVIEVDVIPQHSECREDYFQIKMQHLK-NQ--TWQQSSKYSVFVRDGPQSKDIV-----GNKADFKAFKLDLKAVA-DSRKEAET-
129 ref|XP_055445695.1|  99.1%  75.7%   QYFE-DHQVQKAKNCIREPRFVEVLLPNSTLSNRFVIEVDVVPKYSECEVDYFQIKMQNCS-NT--IWKQSPNFSVFVRDGANSKNII-----NNNAGFKAFKLDLKRLA-ESRKEAEE-
130 ref|XP_061269427.1|  99.1%  76.1%   QYFE-DHQVQKAKNCIREPRFVEVLLPNSTLSDRFVIEVDVVPKYSECEVDYFQIKMQNCS-NT--IWKQSPNFSVFVRDGASSKNII-----NNNAGFKAFKLDLKKLA-ESRKEAEE-
131 ref|XP_012881537.1| 100.0%  77.0%   QYFE-SHQVQQAKKCIREPRFVEVLLPNSTLSDRFVIEVDVIPQYSECGQDYFQIKMQNHS-NN--TWKPSSKYSVFVRDGPNCKNIL-----KNKTDFTAFKLDLKAMS-ESRKEAEE-
132 ref|XP_051033135.1| 100.0%  73.6%   QYFE-AHQVQQAKKCIREPRFVEVLLPNSIPSNRFVIEVDVLPQYSECREDYFQIKMQHLK-NQ--TWQQSSKYSVFVRDGPQSKDII-----GNKADFRAFKLDLKAVA-DSRKEVET-
133 ref|XP_069852599.1| 100.0%  76.5%   QYFE-SHQVQQAKKCIREPRFVEVLLPNSTLSDRFVIEVDVIPQYSECGQDYFQIKMQNHS-NN--TWKPSSKYSIFVRDGPNCKNIL-----KNKTDFKAFKLDLKAMS-ESRKEAEE-
134 ref|XP_006078386.4|  99.1%  75.2%   QYFE-DHQVQKAKNCIREPRFVEVLLPNSTLSNRFVIEVDVVPKYSECEVDYFQIKMQNCS-NT--IWKQSPNFSVFVRDGANSKNII-----NNNAGFKAFKLDLKRLA-ESRKEAEE-
135 ref|XP_061269426.1|  99.1%  76.1%   QYFE-DHQVQKAKNCIREPRFVEVLLPNSTLSDRFVIEVDVVPKYSECEVDYFQIKMQNCS-NT--IWKQSPNFSVFVRDGASSKNII-----NNNAGFKAFKLDLKKLA-ESRKEAEE-
136 ref|XP_006078384.4|  99.1%  75.2%   QYFE-DHQVQKAKNCIREPRFVEVLLPNSTLSNRFVIEVDVVPKYSECEVDYFQIKMQNCS-NT--IWKQSPNFSVFVRDGANSKNII-----NNNAGFKAFKLDLKRLA-ESRKEAEE-
137 ref|XP_006991270.1| 100.0%  74.5%   QYFE-VHQVQQAKKCIREPRFVEVLLPNSTPSNKFVIEVDVIPQHAECREDYFQIKMQHLK-NQ--TWQQSSKCSVFVRDGPQSKDII-----GNKADFKAFKLDLKAVA-DSRKEAET-
138 ref|XP_028725617.1| 100.0%  74.5%   QYFE-VHQVQQAKKCIREPRFVEVLLPNSTPSNKFVIEVDVIPQHAECREDYFQIKMQHLK-NQ--TWQQSSKCSVFVRDGPQSKDII-----GNKADFKAFKLDLKAVA-DSRKEAET-
139 ref|XP_008820770.1| 100.0%  76.1%   QYFE-VHQVQQAKKCIREPRFVEVLLPNSTPSDRFVIEVDVIPQYSECGHDYFQIKMQKLD-NK--TWQQSSKYSVFVRDGPQTKDII-----GNKAEFKAFKLNLKTMA-DSRKEAEE-
140 ref|XP_044802364.2|  99.1%  75.2%   QYFE-DHQVQKAKNCIREPRFVEVLLPNSTLSNRFVIEVDVVPKYSECEVDYFQIKMQNCS-NT--IWKQSPNFSVFVRDGANSKNII-----NNNAGFKAFKLDLKRLA-ESRKEAEE-
141 ref|XP_008062458.2| 100.0%  73.9%   QYFE-VHQVQQAKKCIRAPRFVKVLLPDSTLSDRFVIEVDVIPQYAECEDHYFQIKMQIYK-NR--IWKQSPEFSVFVRDGSSSKNII-----KNKVDFKAFRLDLKTLA-ETRREEEG-
142 ref|XP_041531686.1| 100.0%  73.6%   KYFE-VHQVQQAKKCIREPRFVEVLLPNSTPSNRFVIEVDVIPQYSECREDFFQIKMQHLK-NQ--TWQQSSRYSVFVRDGPRSKDII-----GNKVDFKAFKLDLKAVT-ESRKEAEA-
143 ref|XP_040584508.1| 100.0%  73.6%   QYFE-VHQVQQAKKCIREPRFVEVLLPNSTPSNRFVIEVDVLPQHSECREDYFQIKMQHLK-NQ--TWQQSSKYSVFVRDGPRSKDII-----GNKADFKAFKLDLKTVA-DSRKEAET-
144 ref|XP_021045894.1| 100.0%  73.2%   QYFE-VHQVQQAKKCIREPRFVEVLLPNSTPSNKFVIEVDIIPQYSECKEDYFQIKMQCFK-DR--TWQQSPKYSVFVRDGPKSKDIR-----GNKADFKTFTLDLKALA-DCRKETEE-
145 ref|XP_048292645.1| 100.0%  74.0%   RYFE-VHQVQQAKKCIREPRFVEVLLPNSTPSNRFVIEVDVIPQYSECREDFFQIKMQHLK-TQ--TWQPSSKYSVFVRDGPKSKDII-----GNKVDFKAFKLDLKAVT-ESRKEAEA-
146 ref|XP_021045323.1| 100.0%  73.6%   QYFE-VHQVQQAKKCIREPRFVEVLQPNSTPSNKFVIEVDINPQYSECKEDYFQIKMQCLK-DR--TWQQSSKYSVFVRDGPRSKDII-----GNKADFKNFKLDLKALA-DSRKEAEK-
147 ref|XP_042540450.1| 100.0%  76.1%   QYFE-SHQVQQAKKCIREPRFVEVLLPNSTLSGRFVIEVDVIPQYSECGQDYFQIKMQNHS-NN--TWKPSSKYSVFVRDGPKCKNIL-----KNKTDFNVFKLDLKTMS-ESRKEAEE-
148 ref|XP_047418909.1| 100.0%  75.7%   QYFE-AHQVQQAEKCIREPRFVEVLLPNSTVSDRFVIEVDIIPQYSECGYDYFQIKMQNYE-NK--TWEQSSKYSVFVRDGPKSKDII-----KSKSDFKAFKLDLKKVA-DSRKEAEE-
149 ref|XP_034375203.1| 100.0%  73.6%   QYFE-VHQVQQAKKCIREPRFVEVLLPNSTPSNRFVIEVDIIPQYSECKEDYFQIKMQCLK-NQ--AWQQAPNYSVFVRNGPRSKDII-----GNKADFKTFKLDLKALS-ESRKEAEK-
150 ref|XP_038176268.1| 100.0%  73.6%   QYFE-VHQVQQAKKSIREPRFVEVLLPNSTPSNRFVIEVDVIPQYSECREDFFQIKMQHLK-TQ--TWQQSSKYSVFVRDGPKSKDII-----GNKVDFKTFKLDLKAVT-ESRKEAEA-
151 ref|XP_038176270.1| 100.0%  73.6%   QYFE-VHQVQQAKKSIREPRFVEVLLPNSTPSNRFVIEVDVIPQYSECREDFFQIKMQHLK-TQ--TWQQSSKYSVFVRDGPKSKDII-----GNKVDFKTFKLDLKAVT-ESRKEAEA-
152 ref|XP_028634992.1| 100.0%  73.6%   QYFE-VHQVQQAKKCIREPRFVEVLLPNSTPSNKFVIEVDIIPQYSECKEDYFQIKMQCLK-NQ--AWQQVPNYSVFVRDGPKSKDII-----RNKADFKTFKLDLKQLS-EFRKEAEK-
153 ref|XP_032762876.1| 100.0%  72.7%   QYFE-VHQVQQAKKCIREPRFVEVLLPNSTPSNKFVIEVDIIPQYSECKEDFFQIKMHWRK-DE--TWQQIPKYSVFVRDGPKSKDII-----GNKADFKAFKLDLKALA-DSRKEAEK-
154 ref|XP_057634583.1| 100.0%  73.2%   KYFE-VHQVQQAKKCIREPRFVEVLLPNSTPSNRFVIEVDVIPQYSECREDFFQIKMQHLK-NQ--TWQQSSKYSVFVRDGPKSKDII-----GNKVDFKTFKLDLKAVT-ESRKEAEA-
155 ref|NP_001386282.1| 100.0%  72.3%   QYFE-VHQVQQAKKCIREPRFVEVLLPNSTPSNKFVIEVDIIPQYSECKEDFFQIKMHWHK-DE--TWQQIPKYSVFVRDGPKSKDII-----GNKADFKAFKLDLKALA-DSRKEAEK-
156 ref|XP_015362209.1| 100.0%  76.1%   QYFE-AHQVQQAEKCIREPRFVEVLLPNSTVSDRFVIEVDVIPQYAECGEDYFQIKMQNCN-NK--AWEQSSKYSVFVRDGPKSKDIT-----KSKSDFKAFKLDLKKVA-ESRKEAEE-
157 ref|XP_019059962.1|  98.7%  73.1%   QYFE-AHQVQQAKKCIREPRFVEVLLPNSTVSDRFVVEVDVVPQYSVCKHDYFQIKMQDCN-NK--TWQQRSKHSVFVRDGPSTKNVL-----INKADFKAFLLDLKSLA-VSREEAEE-
158 ref|XP_046311157.1| 100.0%  76.1%   QYFE-AHQVQQAEKCIREPRFVEVLLPNSTVSDRFVIEVDVIPQYAECGEDYFQIKMQNCN-NK--AWEQSSKYSVFVRDGPKSKDIT-----KSKSDFKAFKLDLKKVA-ESRKEAEE-
159 ref|XP_027775683.2| 100.0%  76.1%   QYFE-AHQVQQAEKCIREPRFVEVLLPNSTVSDRFVIEVDVIPQYAECGEDYFQIKMQNCN-NK--AWEQSSKYSVFVRDGPKSKDIT-----KSKSDFKAFKLDLKKVA-ESRKEAEE-
160 ref|XP_026247365.1| 100.0%  75.7%   QYFE-AHQVQQAEKCIREPRFVEVLLPNSTVSDRFVIEVDVIPQYAECGEDYFQIKMQNCN-NK--AWEQSSKYSVFVREGPKSKDIT-----KSKLDFKAFKLDLKKVA-ESRKEAEE-
161 ref|XP_005340593.1| 100.0%  76.1%   QYFE-AHQVQQAEKCIREPRFVEVLLPNSTVSDRFVIEVDVIPQYSECGEDYFQIKMQNCN-NK--AWEQSSKYSVFVRDGPKSKDIT-----KSKSDFKAFKLDLKKVA-ESRKEAEE-
162 ref|XP_007652776.1| 100.0%  71.9%   QYFE-AHQVQQAKKCIREPRFVEVLLPNSTPSNKFVIEVDVLPQHSECKEDYFQIKMQHLK-SQ--TWQQSSKHSVFVRDGPQSKDII-----GNKTDFKAFKLGLKEVA-DSRKEAET-
163 ref|XP_055468986.1| 100.0%  71.9%   QYFE-VHQVQQAKKCIREPRFVEVLLPDSTPSNRFVIEVDVIPKYLECKEDYFQIKIQERK-NQ--TWQQSSKYSVFVRDGPRTKDII-----GNKADFKTFKLDLKAVA-DSRKEAED-
164 ref|XP_021508904.1| 100.0%  71.0%   QYFE-VHQVQQAKKCIREPRFVEVLLPDSTPSNKFVIEVDVIPKYLECKEDYFQIKIQEHK-NQ--AWQQSSKYSVFVRDGPRTKDII-----GNKADFKTFKLDLKAVA-DSRKEAED-
165 ref|XP_012791283.2| 100.0%  70.9%   QYFE-EGQVQKAKNCIREPRFVEVLLTNGIPSDRYVIEVDIIPKYSECEHDYFQIKMQNFN-NK--TWNKSSKFSVFVRDGASCKDLM-----KTPADFKAYKSDLKTLA-ESRKEAEE-
166 ref|XP_005388082.1|  98.7%  72.2%   QYFE-VHQVQQAKQCIREPRFVEVLLPNSTVSDRFVVEVDVVAKYSECKLDYFQVKMQNCN-NK--TWQQSSNYSIFVRDGPNSKNII-----KDKVALDVFKLHLKALA-ASRKEAEE-
167 ref|XP_052029157.1| 100.0%  73.6%   QYFE-AHQVQQAKKCIREPRFVEVLLPNSTPSNKFVIEVDIIPQYSECKEDYFQIKMQCFK-NQ--EWQQNPKSLVFVRDGPKSKDII-----GNKADFKTFKLDLKALS-DSRKEAEK-
168 ref|XP_055974093.1| 100.0%  70.0%   QYFE-EGQVQKAKNCVRTPRFVEVLPSDSTPSNKYVIEVDIIPKYSECKHDYFQIKMQNFN-NN--TWNRSPRFSVFVRDGASCKDLI-----KNNADFRAYKLDLKSLA-ESRKEAEE-
169 ref|XP_040821063.1|  99.1%  70.9%   KYFE-EHQVQQAKTCIRGPRFVEVLLPDDTVSDRFVIEVDVIPHYSVCGQDYFQIKKQICN-N--KTWEQSSKFSVFVRDGASNKNMM-----KNKTDFKMFKLDLKTLA-ESRKEAED-
170 ref|XP_045016549.1| 100.0%  68.7%   QYFE-PHQVQQAKKCIREPRFVEVLLPNSSVSDKFVIEVDVVPQYSDCGHNYFQIKLQNLK-NK--AWQQSSNYSIFVRDGPNTNNII-----NENAKLKAFKMDLKTLA-ESREEAEE-
171 ref|XP_051007939.1| 100.0%  69.3%   QYFE-VHQVQQAKKCIREPRFVEVLLPSSAPSNRFVIEVDVIPLHSECREDYFQIRMQHRK-NQ--MWQRSSNSSVFVRDGPKSKDII-----GSKADFKTFKLDLKAMA-DSRKEAEN-
172 ref|XP_004582357.2|  99.1%  69.1%   KYFE-EHQVQQAKTCIQGPRFVEVLLPDDTVSDRFVIEVDVIPHYSVCGQDYFRIKKQICN-N--KTWKQSSEFSVFVRDGASNKNMM-----KNKTDFKMFKLNLKTLA-ESRKEAED-
173 ref|XP_062034088.1|  99.6%  68.8%   KYFE-EHQVQQAKSCIRVPRFVEVLLPDSTVSDRFVIEVDVIPHYSVCGHDYFQIKKQIYN-NDNKKWEQSSKFSVLVRDGARTVNIV-----KNKTDFKMFKLNLKTLA-ESRKEAED-
174 ref|XP_051708952.2|  99.6%  68.0%   KYFE-EHQVQQAKSCIRVPRFVEVLLPDSTVSDRFVIEVDVIPHYSVCGHDYFQIKKQIYD-NNNKKWEQSSKFSVLVRDGARTVNIV-----KTKTDFKMFKLNLKTLA-ESRKEAED-
175 ref|XP_012373928.2|  98.7%  73.5%   QYFE-EHQVQQAKKCIREPRFVVVLLPNSIPSDRFVIEVDVIPKHSVCENDYFQIKMQNCN-NN--TWGKSPKFSVFVRDGANSVDIM-----KNKAKFKAFKLDLKTMA-ESRREAEE-
176 ref|XP_015331500.1|  78.7%  79.0%   QYFE-AHQVQQAEKCIREPRFVEVLLPNSTVSDRFVIEVDVIPQYAECGEDYFQIKMQNCN-NK--AWEQS-------------------------------------------------
177 ref|XP_005340592.1|  98.7%  64.4%   KYFE-ESEINEAKRCIREPRFVEVLLQNNTLSDRFVIEVDIIPKYSICKEKYFYIKMQCCT-DK--MWKQHQDHSLFIREGASSRDIL-ANSKQRDIDFKKFLQNLKSLV-ASRKEAEEE
178 ref|XP_015362203.1|  98.7%  64.4%   KYFE-ESEINEAKRCIREPRFVEVLLQNNTSSDRFVIEVDIIPKHSICKEKYFYIKMQCCT-DK--MWKQHQDHSLFIREGASSRDIL-ANRKQRDIDFKKFLQNLKSLV-ASRKEAEEE
179 ref|XP_046311151.1|  98.7%  64.4%   KYFE-ESEINEAKRCIREPRFVEVLLQNNTSSDRFVIEVDIIPKHSICKEKYFYIKMQCCT-DK--MWKQHQDHSLFIREGASSRDIL-ANRKQRDIDFKKFLQNLKSLV-ASRKEAEEE
180 ref|XP_050641313.1|  98.7%  64.0%   KYFE-ESEINQAKKCIREPRFVEVLLQNNMPSDRFVIEVDIIPKYSICKDKYFYIQMQIFK-DK--IWKQNQNSSLFVREGASSRDIL-ANSKQRDIDFKAFLQNLKSLV-ASRKEAEEE
181 ref|XP_005550273.1|  98.7%  64.4%   KYFE-ESEINQAKKCIREPRFVEVLLQNNMPSDRFVIEVDIIPKYSICKDKYFYIQMQIFK-DK--IWKQNQNPSLFVREGASSRDIL-ANSKQRDVDFKAFLQNLKSLV-ASRKEAEEE
182 ref|XP_014989728.2|  98.7%  64.4%   KYFE-ESEINQAKKCIREPRFVEVLLQNNMPSDRFVIEVDIIPKYSICKDKYFYIQMQIFK-DK--IWKQNQNPSLFVREGASSRDIL-ANSKQRDVDFKAFLQNLKSLV-ASRKEAEEE
183 ref|XP_062956902.1|  98.7%  64.8%   KYFE-ENEINKARKCIREPRFVEVLLQNNMPSDRFVIEVDIIPKYSVCKEKYFYIKMQNCK-NK--TWKQNQDHSLFVREGASSKDIL-ANVKQRDIEFKAFLQNVKAWA-ASRKKAEEE
184 ref|XP_027775676.2|  98.7%  64.4%   KYFE-ESEINEAKRCIREPRFVEVLLQNNTSSDRFVIEVDIIPKHSICKEKYFYIKMQCCT-DK--MWKQHQDHSLFIREGASSRDIL-ANSKQRDIDFKKFLQNLKSLV-ASRKEAEEE
185 ref|XP_008570541.1|  98.7%  64.4%   KYFE-ENEINKAKKCIREPRFVEVLLQNNMPSDRFVIEVDIIPKYSVCKEKYFYIKMQNCK-NK--TWKQNEDHSLFVREGASSKDIL-ANVKQRDIEFKAFLQNVKSWA-ASRKKAEEE
186 ref|XP_008259950.2|  98.7%  63.6%   KYFE-DSDINEAKKCIREPRFVEVLLQNSMPSDRFVIEVDVIPKHSVCKEKYFYIKMQSCK-DN--VWKQNQEYSLFVREGASSRDIL-ANAKQRDPDFKAFTQNLKSLV-ASRKEAEEE
187 ref|XP_065398409.1|  98.7%  64.0%   KYFE-ESEINQAKKCIREPRFVEVLLQNNMPSDRFVIEVDIIPKYSICKDKYFYIQMQIFK-DK--IWKQNQNSSLFVREGASSRDIL-ANSKQRDVDFKAFLQNLKSLV-ASRKEAEEE
188 ref|XP_011729134.2|  98.7%  64.0%   KYFE-ESEINQAKKCIREPRFVEVLLQNNMPSDRFVIEVDIIPKYSICKDKYFYIQMQIFK-DK--IWKQNQNSSLFVREGASSRDIL-ANSKQRDVDFKAFLQNLKSLV-ASRKEAEEE
189 ref|XP_027973811.1|  98.7%  63.1%   QYFE-ENEINEAKKCIREPRFVEVLLQNNTPSDRFVIEVDVIPKYSVCEKKYFLIKMQNCK-NE--TWKPNQDFSLFVRDGASSKDVL-ANVKQRDIAYREFLQNLESVA-ASRKVAEEE
190 ref|XP_014643659.1|  98.7%  63.1%   QYFE-ESEIKVAKKCIREPRFVEVLLQNNTPSDRFVIEVDVIPKHSVCKEKYFYIKMQNCK-ND--TWKQNQDLSLFVREGASSKDIL-ANVKQRDIDFKAFTKNLKSLV-TARKEAEEE
191 ref|XP_032108853.1|  98.7%  64.0%   KYFE-ESEINEAKKCIREPRFVEVLLQNNTPSDRFVIEVDIIPKHSVCKDKYFYIQMQSCT-DK--IWKQNQNSSLFVREGASSRDIL-ANPNKRDVDFKAFSQKLKSLV-ASRKEAEEE
192 ref|XP_066094273.1|  98.3%  63.3%   QYFE-DSDISEAKKCIRQPRFVEVLLQNNTLSDRFVIEVDVIPKYSVCKEKYFYIKMQNCK-DE--TWKQNQDISLFVRDGASSKDIL-ANVKQRDVDFKTFFRKLNSLA-ASRKEAEEE
```

193	ref|XP_028381763.1|	98.7%	63.1%	KYFE-DSEINEARKCIREPRFVEVLLQDSTPSDRFVIEVDVIPKHSVCKEKYFYIMMQTYE-NE--TWKQSEELSLFVREGASSKDIR-ANVKRRDVEFKAFFKKLKSLA-VSRKEAEEK
194	ref|XP_046526743.1|	98.7%	63.6%	QYFE-ESEIKVAKKCIREPRFVEVLLQNNTPSDRFVIEVDVIPKHSICKEKYFYIKMQNCK-NE--KWKQNEDHSLFVRDGASSKDIL-ANGKQRDIDFKAFMQNLKSLV-TSRKEAEEE
195	ref|XP_058381591.1|	98.7%	63.1%	QYFE-ESEIKVAKKCIREPRFVEVLLQNNTPSDRFVIEVDVIPKHSVCKEKYFHIKMQNCK-ND--TWKQNQDLSLFVREGASSKDIL-ANIKQRDIDFKAFIKNLKSLV-TSRKEAEEE
196	ref|XP_027429555.1|	98.7%	62.3%	QYFE-ENEINEAKKCIREPRFVEVLLQNNTPSDRFVIEVDVIPKYSVCEKKYFLIRMQNCK-NE--TWKPNQDLSLFVRDGASSKDVL-ANVKQRDIAYREFLQNLESVA-ASRKVAEEE
197	ref|XP_017380582.1|	98.7%	63.6%	KYFE-ESEINEAKKCIREPRFVEVLLQNNTPSDRFVIEVDIIPKHSVCKDKYFYIQMQSCT-DK--IWKQNQNSSLFVREGASSKDIL-ANPNKRDVDFKAFSQKLKSLV-ASRKEAEEE
198	ref|XP_066210387.1|	98.3%	63.3%	QYFE-DSDISEAKKCIRQPRFVEVLLQNNTLSDRFVIEVDVIPKYSVCKEKYFYIKMQNCK-DE--TWKQNQDISLFVRDGASSRDIL-ANVKQRDVDFKTFFRKLNSLA-ASRKEAEEE
199	ref|XP_016074281.1|	98.7%	64.4%	QYFE-ESEINEAKKCIREPRFVEVLLQNSTPSDRFVIEVDIIPKHSVCKEKYFYIKLQNYE-TG--TWKQSQDTSLFVRDGASSKDIL-ANVKQRDVAFKAFLKKLKSLV-ASRKEAEEE
200	ref|NP_001244057.1|	98.7%	63.6%	QYFE-ESEIKVAKKCIREPRFVEVLLQNNTPSDRFVIEVDVIPKHSICKEKYFYIKMQNCK-NE--KWKQNEDHSLFVRDGASSKDIL-ANGKQRDTDFKAFTQNLKSLV-TSRKEAEEE
201	ref|XP_040821033.1|	98.7%	62.7%	KYFE-ENEVTIAKKCIREPRFVEVLLQNNMPSDRVVIEVDIIPKHSVCKKRYFYVKMQSCK-NK--VWKQNQECSLFIREGASSRDIL-ANGRQRDVDFKAFTQNLEALV-ASRKEAEEK
202	ref|XP_063098771.1|	98.3%	62.0%	KYFE-ESEISEAQKCIRQPRFIDVLLQNNTLSDRFVIEVDIIPKYSVCKEKYFYIKLQSLT-DK--TWKQNQNLSLFVRDGASSKDIL-ANVKRREIEFKAFYENLKSCA-AARKDAEEE
203	ref|XP_007980344.1|	98.7%	64.0%	KYFE-ESEINQAKKCIREPRFVEVLLQNNMPSDRFVIEVDIIPKHSICKNKYFYIQMQICK-DK--IWKQNQNPSLFVREGASSRDIL-ANSKQRDVDFKAFLQNLKSLV-ASRKEAEEE
204	ref|XP_070471752.1|	98.7%	63.6%	QYFE-ESEIKVAKKCIREPRFVEVLLQNNTPSDRFVIEVDVIPKHSICKEKYFYIKMQNCK-NE--KWKQNEDHSLFVRDGASSKDIL-ANGKQRDTDFKAFTQNLKSLV-TSRKEAEEE
205	ref|XP_017812450.3|	98.7%	63.6%	KYFE-ESEINQAKKCIREPRFVEVLLQNNMPSDRFVIEVDIIPKHSICKDKYFYIQMQICK-DK--IWKQNQNPSLFVREGASSRDIL-ANSKQRDIDFKAFLQNLKSLV-ASRKEAEEE
206	ref|XP_011853657.1|	98.7%	63.6%	KYFE-ESEINQAKKCIREPRFVEVLLQNNMPSDRFVIEVDIIPKHSICKDKYFYIQMQICK-DK--IWKQNQNTSLFVREGASSRDIL-ANSKQRDIDFKEFLQNLKSLV-ASRKEAEEE
207	ref|XP_026247361.1|	98.7%	63.1%	KYFE-ESEINEVKRCIREPRFVEVLLQNNTSSDRFVIEVDIIPKHSICKEKYFYIKMQCCT-DK--TWKQHQDHSLFIREGASSRDIL-ANSKQRDIDFKKFLQNLKSVV-ASRKEAEEE
208	ref|XP_012306232.2|	98.7%	63.1%	KYFE-ESEINEAKKCIREPRFVEVLLQNNTPSDRFVIEVDISPKHSVCKDKYFYIQMQSCT-DK--IWKQNQNPSLFVREGASSRDIL-ANPNKRDVDFKAFLQNLESLV-ASRKEAEEE
209	ref|XP_024432967.2|	98.7%	62.3%	KYFE-DSEINEARKCIREPRFVEVLLQDNTPSDRFVIEVDVIPKHSVCKEKYFYIIMQTHE-NE--TWKQSDELSLFVREGASSKDIR-ANVKQRDVEFKAFFKKLKSLA-VSRKEAEEK
210	ref|XP_008062456.1|	98.7%	64.4%	KYFE-ESEINQAKKCIREPRFVEVLLQNSTPSDRFVIEVDTVPKHSVCKDKYFYIQMQSCT-NK--VWKQNQDNSLFVREGASSKDIL-ANVKRRDVDFKEFLRSLKSLT-ASRKEAEEE
211	ref|XP_020016743.1|	98.7%	61.9%	KYFE-DSEINVAKKCIREPRFVEVLLQNNTLSDRFVIEVDVIPKHSVCKKKYFYVKMQSFT-DK--TWKQSQDHSLFVREGASCKNIL-ANAKQRDINFKEFEDNLKSWI-ACRKQAEEE
212	ref|XP_045684040.1|	98.7%	62.3%	KYFE-DSEINEARKCIREPRFVEVLLQDSTPSDRFVIEVDVIPKHSVCKEKYFYIMMQTHE-NE--TWKHSEELSLFVREGASSKDIR-ANVKRRDVEFKAFFKKLKSLA-LSRKEAEEK
213	ref|XP_011929253.1|	98.7%	63.1%	KYFE-ESEINQAKKCIREPRFVEVLLQNNMPSDRFVIEVDIIPKHSICKDKYFYIQMQICK-DK--IWKQNQNPSLFVREGASSRDIL-ANSKQRDIDFKEFLQNLKSLV-ASRKEAEEE
214	ref|XP_004582358.2|	98.7%	62.3%	KYFE-DNEVTIAKKCVREPRFVEVLLQNNMPSDRVVIEVDIIPKHSICKKRYFYVKMQSCK-DK--VWKQNQECSLFVREGASSKDIL-ANGRQRDVDFKAFTQNLEALV-ASRKEAEEK
215	ref|XP_053511032.1|	98.7%	62.7%	KYFE-DSEINEAKKCIREPRFVEVLLQDNTPSDRFVIEVDVIPKHSVCKEKYFYIMMQTLE-NE--TWKQSEELSLFVREGASSKDIR-ANVKQRDVEFKAFFKKLKSLA-VSRKEAEEK
216	ref|XP_025237015.1|	98.7%	63.1%	KYFE-ESEINQAKKCIREPRFVEVLLQNNMPSDRFVIEVDIIPKHSICKDKYFYIQMQICK-DK--IWKQNQNPSLFVREGASSRDIL-ANSRQRDIDFKEFLQNLKSLV-ASRKEAEEE
217	ref|XP_009451890.1|	98.7%	63.1%	KYFE-ESEINEAKKCIREPRFVEVLLQNNTPSDRFVIEVDTIPKHSICNDKYFYIQMQICK-DK--IWKQNQNLSLFVREGASSRDIL-ANSKQRDVDFKAFLQNLKSLV-ASRKEAEEE
218	ref|NP_001290425.1|	98.7%	63.1%	KYFE-ESEINEAKKCIREPRFVEVLLQNNTPSDRFVIEVDTIPKHSICNDKYFYIQMQICK-DK--IWKQNQNLSLFVREGASSRDIL-ANSKQRDVDFKAFLQNLKSLV-ASRKEAEEE
219	ref|XP_008960246.3|	98.7%	63.1%	KYFE-ESEINEAKKCIREPRFVEVLLQNNTPSDRFVIEVDTIPKHSICNDKYFYIQMQICK-DK--IWKQNQNLSLFVREGASSRDIL-ANSKQRDVDFKAFLQNLKSLV-ASRKEAEEE
220	ref|XP_012415619.1|	98.7%	61.4%	QYFE-GNEINEAKKCIREPRFVEVLLQNNTPSDRFVIEVDVIPKYSVCEKKYFLIRMQNCK-NE--TWKPNQDLSLFVRDGASSKDVL-ANVKQRDIAYKEFLQNFESVA-ASRKEAEKE
221	ref|XP_008684459.2|	98.7%	62.3%	QYFE-GNEINEAKKCIREPRFVEVLLQNNTPSDRFVIEVDVIPKYSVCGEKYFLIRMQSCK-NE--TWKPNQDLSLFVRDGPSSKDIL-ANVKQRAIAYKEFSQNFKSVA-ASRKVAEEE
222	ref|XP_036902380.1|	98.7%	61.4%	KYFE-DSEINEAKKCIREPRFVEVLLQDNTPSDRFVIEVDVIPKHSVCKEKYFYIMMQTHE-NE--TWKQSEELSLFVREGASTKDIR-ANVKQRDVEFKAFFKKLKSLA-VSRKEAEEK
223	ref|XP_026361422.1|	98.7%	62.3%	QYFE-GNEINEAKKCIREPRFVEVLLQNNTPSDRFVIEVDVIPKYSVCGEKYFLIRMQSCK-NE--TWKPNQDLSLFVRDGPSSKDIL-ANVKQRAIAYKEFSQNFKSVA-ASRKVAEEE
224	ref|XP_002918280.1|	98.7%	62.3%	QYFE-GNEINEAKKCIREPRFVEVLLQNNTPSDRFVIEVDVIPKYSVCGEKYFLIRMQSCK-NE--TWKPNQDLSLFVRDGPSSKDIL-ANVKQRDIAYKEFSQNFKSVA-TSRKVAEEE
225	ref|XP_054351924.1|	98.7%	62.7%	KYFE-ESEINEAKKCIREPRFVEVLLQNNTPSDRFVIEVDIIPKHSICKDKYFYIQMQICK-DK--TWKQNQNLSLFVREGASSRDIL-ANSKQRDVDFKAFLQNLKSLV-ASRKEAEEE
226	ref|XP_009241290.3|	98.7%	62.7%	KYFE-ESEINEAKKCIREPRFVEVLLQNNTPSDRFVIEVDIIPKHSICKDKYFYIQMQICK-DK--TWKQNQNLSLFVREGASSRDIL-ANSKQRDVDFKAFLQNLKSLV-ASRKEAEEE
227	ref|XP_037692600.1|	98.7%	62.7%	QYFE-ESEIYEAKKCIREPRFVEVLLQNNTLSDRFVIEVDVIPKHSICKEKYFYIKMQNFK-DE--TWKQSQDSSLFVRDGASSKDIL-ANVRQRDADFKAFMQNFKSLV-ARRKEAEEE
228	ref|XP_037692601.1|	98.7%	62.7%	QYFE-ESEIYEAKKCIREPRFVEVLLQNNTLSDRFVIEVDVIPKHSICKEKYFYIKMQNFK-DE--TWKQSQDSSLFVRDGASSKDIL-ANVRQRDADFKAFMQNFKSLV-ARRKEAEEE
229	ref|XP_010343634.2|	98.7%	62.7%	KYFE-ESEINEAKKCIREPRFVEVLLQNNTPCDRFVIEVDIIPKHSVCKDKYFYIQMQSCT-DK--IWKQNQNPSLFVREGASSRDIL-ANPNKRDVDFKAFLQKLESLV-ASRKEAEEE
230	ref|XP_021545299.1|	98.7%	62.7%	QYFE-ENEINEAKKCIREPRFVEVLLQNNTPSDRFVIEVDVIPKHSVCEEKYFLIKMQSCK-NE--TWKPNQDFSLFVRDGPSSKDLL-ANVKQRDVAHRNFLQNLKSVV-ASRKVAEEE
231	ref|XP_047418554.1|	98.7%	61.9%	KYFE-ESEINEARKCIREPRFVEVLLQNNTSSDRFVIEVDVIPKHSICKEKYFYIKMQSRS-DK--TWKQNQDHSLFIREGASTRDIL-ANHKQRDIDFKIFLQNLKSLV-ASRKEAEEE
232	ref|XP_047418561.1|	98.7%	61.9%	KYFE-ESEINEARKCIREPRFVEVLLQNNTSSDRFVIEVDVIPKHSICKEKYFYIKMQSRS-DK--TWKQNQDHSLFIREGASTRDIL-ANHKQRDIDFKIFLQNLKSLV-ASRKEAEEE
233	ref|XP_025728670.1|	98.7%	61.2%	QYFE-ENEINEAKKCIREPRFVEVLLQNNTPSDRFVIEVDVIPKYSVCEKKYFLIRMQSCK-NE--TWKPNQDLSLFVRDGASSKDVLLANVKQRHIAYKEFLQNLESVA-ASRKVAEEE
234	ref|XP_018887315.2|	98.7%	62.3%	KYFE-ESEINEAKKCIREPRFVEVLLQNNTPSDRFVIEVDTIPKHSICNDKYFYIQMQICK-DK--IWKQNQNLSLFIREGASSRDIL-ANSKQRDVDFKAFLQNLKSLV-ASRKEAEEE
235	ref|XP_010601767.1|	98.3%	62.9%	KYFK-ESEISKAKKCIREPRFVEVLLQNSTPSDRFVIEVDIIPKYSVCKETYFYIKMYSLI-DK--TWKQNQGHSLFVRDGASSKDIL-ANVNQRDIDFKTFLKNLKSCV-AFRKEAEEE
236	ref|XP_036902379.1|	98.7%	61.0%	KYFE-DSEINVARKCIREPRFVEVLLQDNTPSDRFVIEVDVIPKHSVCKEKYFYIMMQTHE-NE--TWKQSEELSLFVREGASTKDIR-ANVKQRDVEFKAFFKKLKSLA-VSRKEAEEK
237	ref|XP_070280369.1|	98.7%	63.3%	QYFE-ESEINEAKKCIREPRFVEVLLQNNTPSDRFVIEVDVIPKHSVCKEKYFYIKMQNCK-AE--IWKQNQDASLFVRDGASSKDIL-ANVKQRDKDFKAFIKKVKSLA-VSRKEAEEE
238	ref|XP_032616947.1|	98.7%	62.7%	KYFE-ESEINEAKKCIREPRFVEVLLQNNTPSDRFVIEVDINPKHSICKDKYFYIQMQICK-DK--IWKQNQNTSLFVREGASSRDIL-ANSKQRDVDFKAFLQNLKSLV-ASRKEAEEE
239	ref|XP_030677948.1|	98.7%	62.7%	KYFE-ESEINEAKKCIREPRFVEVLLQNNTPSDRFVIEVDINPKHSICKDKYFYIQMQICK-DK--IWKQNQNTSLFVREGASSRDIL-ANSKQRDVDFKAFLQNLKSLV-ASRKEAEEE
240	ref|XP_036184071.1|	98.7%	63.3%	QYFE-ESEINEAKKCIREPRFVEVLLQNNTPSDRFVIEVDVIPKHSVCKEKYFYIKMQNCK-AE--IWKQNQDASLFVRDGASSKDIL-ANVKQRDKEFKAFTKKVKSLA-VSRKEAEEE
241	ref|XP_004776483.1|	98.7%	62.3%	QYFE-GNEVNEAKKCIREPRFVEVLLQNNTPSDRFVIEVDVIPKHSVCEEKYFLIRMQNCK-NE--TWKPNQDLSLFVRDGPSSKDIL-ANVKQRDRVYKEFLQNLKSVV-ASRKEAEEE
242	ref|XP_004389713.1|	98.7%	63.1%	QYFE-ESHIKEAKKCIREPRFVEVLLQNNTPSDKFVIEVDIIPKHSVCKDKYFYINMQNCK-DG--SWRQDKDASLFVREGASSKNIF-ANVRQQDADFKAFLLNVKPLA-ASRRKAEEE
243	ref|XP_049749840.1|	98.7%	62.7%	QYFE-DSHIKEAEKCIRQPRFVEVLQQNNTPSDKFVIEVDVIPRHSVCKDKYFYINMQICK-DG--SWRQDKVCSLFVRDGASSKDIL-ANVRQRDADFKAFSRNFESVV-ASRKEAEEE
244	ref|XP_059252834.1|	98.7%	62.3%	QYFE-GNEVNEAKKCIREPRFVEVLLQNNTPSDRFVIEVDVIPKHSVCEEKYFLIRMQNCK-NE--TWKPNQDLSLFVRDGPSSKDIL-ANVKQRDRVYKEFLQNLKSVV-ASRKEAEEE
245	ref|XP_033050274.1|	98.7%	62.7%	KYFE-ESEINEAKKCIREPRFVEVLLQNNMPSDRFVIEVDIISKHSICKDKYFYIHMQICK-DK--VWKQNQNPSLFVREGASSRDIL-ANSKQWDVDFKAFLQNLKSLV-ASRKEAEEE
246	ref|XP_055128669.1|	98.7%	62.3%	KYFE-ESEINEAKKCIREPRFVEVLLQNNTPSDRFVIEVDINPKHSICKDKYFYIQMQICK-DK--IWKENQNTSLFVREGASSRDIL-ANSKQRDVDFKAFLQNLKSLV-ASRKEAEEE
247	ref|XP_010351460.2|	98.7%	62.7%	KYFE-ESEINEAKKCIREPRFVEVLLQNNMPSDRFVIEVDIISKHSICKDKYFYIHMQICK-DK--VWKQNQNPSLFVREGASSRDIL-ANSKQRDVDFKAFLQNLKSLV-ASRKEAEEE
248	ref|XP_019585266.1|	98.7%	62.4%	QYFE-ESDISEAKRCIREPRFVDVLLQNSTPSDRFVIEVDVIPKHSICKEKYFYIKMQNCQ-DD--TWKQSQDHSLFVREGASSKDIL-ANVKRRDVDFKAFMENLKSLV-ASRKEAEEE
249	ref|XP_046953376.1|	98.3%	62.3%	QYFE--DEVNEAKKCIREPRFVEVLLQNNTPSDRFVIEVDVIPKHSVCEEKYFLIRMQNCK-NE--TWKPNQDLSLFVRDGASSKDIL-ANVKQRDIAYKAFLQNLKSLA-ASRKEAEEE
250	ref|XP_011361946.1|	98.7%	61.9%	QYFE-ESDINKAKKCIREPRFVEVLLQNNTSSGRFVIEVDVIPKHSVCKETYFYIKMQNCK-NE--TWKQNQDHSLFVREGASSKDIL-ANVKQRDVDFKKYIQNLKSLT-ASRKEAEER
251	ref|XP_040328613.1|	98.3%	62.3%	QYFE--DEVNEAKKCIREPRFVEVLLQNNTPSDRFVIEVDVIPKHSVCEEKYFLIRMQNCK-NE--TWKPNQDLSLFVRDGASSKDIL-ANVKQRDIAYKAFLQNLKSLA-ASRKEAEEE
252	ref|XP_032160436.1|	98.7%	62.3%	QYFE-GNEVNEAKKCIREPRFVEVLLQNNTPSDRFVIEVDVIPKHSVCEEKYFLIRMQNCK-NE--TWKPNQDLSLFVRDGPSSKDIL-ANVKQRDRVYKEFVQNLKSVV-ASRKEAEEE
253	ref|XP_003982796.1|	98.3%	62.3%	QYFE--DEVNEAKKCIREPRFVEVLLQNNTPSDRFVIEVDVIPKHSVCEEKYFLIRMQNCK-NE--TWKPNQDLSLFVRDGASSKDIL-ANVKQRDIAYKAFLQNLKSLA-ASRKEAEEE
254	ref|XP_014922007.2|	98.3%	62.3%	QYFE--DEVNEAKKCIREPRFVEVLLQNNTPSDRFVIEVDVIPKHSVCEEKYFLIRMQNCK-NE--TWKPNQDLSLFVRDGASSKDIL-ANVKQRDIAYKAFLQNLKSLA-ASRKEAEEE
255	ref|XP_025785047.1|	98.3%	62.3%	QYFE--DEVNEAKKCIREPRFVEVLLQNNTPSDRFVIEVDVIPKHSVCEEKYFLIRMQNCK-NE--TWKPNQDLSLFVRDGASSKDIL-ANVKQRDIAYKAFLQNLKSLA-ASRKEAEEE
256	ref|XP_030163818.1|	98.3%	62.3%	QYFE--DEVNEAKKCIREPRFVEVLLQNNTPSDRFVIEVDVIPKHSVCEEKYFLIRMQNCK-NE--TWKPNQDLSLFVRDGASSKDIL-ANVKQRDIAYKAFLQNLKSLA-ASRKEAEEE
257	ref|XP_047698808.1|	98.3%	62.3%	QYFE--DEVNEAKKCIREPRFVEVLLQNNTPSDRFVIEVDVIPKHSVCEEKYFLIRMQNCK-NE--TWKPNQDLSLFVRDGASSKDIL-ANVKQRDIAYKAFLQNLKSLA-ASRKEAEEE
258	ref|XP_043444794.1|	98.3%	62.3%	QYFE--DEVNEAKKCIREPRFVEVLLQNNTPSDRFVIEVDVIPKHSVCEEKYFLIRMQNCK-NE--TWKPNQDLSLFVRDGASSKDIL-ANVKQRDIAYKAFLQNLKSLA-ASRKEAEEE
259	ref|XP_025859549.1|	98.7%	60.2%	QYFE-ESEINEVKKCIREPRFVEVLLQNNTLSDRFVIEVDVIPKHSVCEKKYFLIRMQNFK-SE--TWKPNQDLSLFVRDGPSTKDIL-ANVKQRELTFKAFLQNLKSVV-ASRKEAEEE
260	ref|XP_011810448.1|	98.7%	62.3%	KYFE-ESEINEAKKCIREPRFVEVLLQNNMPSDRFVIEVDIISKHSICKDKYFYIHMQICK-DK--VWKQNQNPSLFVREGASSRDIL-ANSKQRDVDFKAFLQNLKSLV-ASRKEAEEE
261	ref|XP_032258048.1|	98.7%	61.4%	QYFE-ESEINEAKKCIREPRFVEVLLQNNTPSDRFVIEVDVIPKHSVCEEKYFLIKMQNCK-NG--TWKPNQDSSLFVRDGPSSKDVL-ANVKQRDIAYKNFSQNLKSVV-ASRKVAEEE
262	ref|XP_032944614.1|	98.7%	62.4%	QYFE-DSDINEAKKCIREPRFVDVLLQNNTPSDRFVIEVDVIPKHSICKEKYFYIKMQNCK-DE--TWKQNQDHSLFVREGASSKDIL-ANVKQRDMDFKAFMENLKPLA-ASRKEAEEE
263	ref|XP_059027098.1|	98.7%	61.9%	QYFE-GNEVNEAKKCIREPRFVEVLLQNNTPSDRFVIEVDVIPKHSVCEEKYFLIRMQNCK-NE--TWKPNQDLSLFVRDGPSSKDIL-ANVKQRDRVYKEFLQNLKSVV-ASRKEAEEE
264	ref|XP_043821279.1|	98.7%	61.4%	HYFE-DNQVNKARKCIREPRFVEVLLQDNTLSDRFVIEVDVIPQYSICKKTTFSIKMQNNK-DK--RWKEDIECSFFVREGASSKNIM-ANVKQNDADYKMFLLNLDSLA-NSRKSAEEE
265	ref|XP_045720657.1|	98.7%	61.4%	QYFE-ENDINEAKKCIREPRFVEVLLQNNTPSDRFVIEVDVIPKHSVCEEKHFLIKMQSCK-NE--TWKPNQDFSLFVRDGPSSKDVL-ANVKQREVAHKNFLQNLKSVV-ASRKVAEEE
266	ref|XP_062034304.1|	97.8%	62.3%	KYFE-DSDIKEAKKCIREPRFVEVLLQNGMPSDRFVIEVDVIPKHSVCKEKYFYIKMQSCK-DE--VWKQNKEYSLFVREGASSKDIL-ANANQRDPDFRAFTQNLKSLV-ASRKEAEEE
267	ref|XP_049498825.1|	98.3%	62.3%	QYFE--DDVNEAKKCIREPRFVEVLLQNNTPSDRFVIEVDVIPKHSVCEEKYFLIRMQNCK-NE--TWKPNQDLSLFVRDGASSKDIL-ANVKQRDIAYKAFLQNLKSLA-ASRKEAEEE
268	ref|XP_034876457.1|	98.7%	61.4%	QYFE-ENDINEAKKCIREPRFVEVLLQNNTPSDRFVIEVDVIPKHSVCEEKHFLIKMQSCK-NE--TWKPNQDFSLFVRDGPSSKDVL-ANVKQREVAHKNFLQNLKSVV-ASRKVAEEE
269	ref|XP_023082516.2|	98.7%	61.9%	KYFE-ESEINEAKKCIREPRFVEVLLQNNMPSDRFVIEVDIISKHSICKDKYFYIHMQICK-DK--VWKQNQNPSLFVREGASSRDIL-ANSKQRDIDFKAFLQNLKSLV-ASRKEAEEE
270	ref|XP_007076766.2|	98.3%	62.3%	QYFE--DDVNEAKKCIREPRFVEVLLQNNTPSDRFVIEVDVIPKHSVCEEKYFLIRMQNCK-NE--TWKPNQDLSLFVRDGASSKDIL-ANVKQRDIAYKAFLQNLKSLA-ASRKEAEEE
271	ref|XP_045350732.1|	98.3%	62.3%	QYFE--DEVNEAKKCIREPRFVEVLLQNNTPSDRFVIEVDVIPKHSVCEEKYFLIRMQNCK-NE--TWKPNQDLSLFVRDGASSKDIL-ANVKQRDIAYKAFLQNLKPLA-ASRKEAEEE
272	ref|XP_058581065.1|	98.3%	62.3%	QYFE--DDVNEAKKCIREPRFVEVLLQNNTPSDRFVIEVDVIPKHSVCEEKYFLIRMQNCK-NE--TWKPNQDLSLFVRDGASSKDIL-ANVKQRDIAYKAFLQNLKSLA-ASRKEAEEE
273	ref|XP_016016798.2|	98.7%	61.4%	QYFE-ESDINKAKKCIREPRFVEVLLQDNTSSGRFVIEVDVIPKQSVCKENYFYIKMQNCK-NE--TWKQNQDHSLFVREGASSKDIL-ANVKQRDVEFKKYTKNLKSLI-ASRKEAEER
274	ref|XP_069852600.1|	98.7%	62.7%	KYFE-ESEIQEAKKCIREPRFVEVLLQNNTPSDRFVIEVDIIPKHSVCKEKYFYIKMQNNA-DK--TWKQDQDHSLYVREGASSKNIL-ASVKKRDTEFKAFLENLKSWI-ASRKEAEEE
275	ref|XP_015444939.1|	98.7%	61.4%	QYFE-ESDINKAKKCIREPRFVEVLLQNNTSSGRFVIEVDVIPKHSVCKETYFYIKMQNCK-NE--TWKQNQDHSLFVREGASSKDIL-ANVKQQDADFKKYIQNLKSLT-ASRKEAEER
276	ref|XP_036875742.1|	98.7%	59.3%	QYFE-ESEINEVKKCVREPRFVDVLLQNNTPSDRFVIEVDVIPKYSVCKQKYFYIKMQNCK-NE--TWKPNQDHSLFVRDGASSKDIL-ANVRQRDIDFKAFIQNLKSLV-ASRKEAEKE
277	ref|XP_039734028.1|	98.7%	61.4%	QYFE-ESDINKAKKCIREPRFVEVLLQNNTSSGRFVIEVDVIPKHSVCKETYFYIKMQNCK-NE--TWKQNQDHSLFVREGASSKDIL-ANVKQQDADFKKYIQNLKSLT-ASRKEAEER
278	ref|XP_042540451.1|	98.7%	62.7%	KYFE-ESEIQEAKKCIREPRFVEVLLQNNTPSDRFVIEVDIIPKHSVCKEKYFYIKMQNNA-DK--TWKQDQDHSLYVREGASSKNIL-ASVKKRDTEFKAFLENLKSWI-ASRKEAEEE
279	ref|XP_041583429.1|	98.7%	60.2%	QYFE-EREINEVKKCIREPRFVEVLLQNNTLSDRFVIEVDVIPKHSVCEKKYFLIRMQNCK-SE--TWKPNQDLSLFVRDGPSTKDIL-ANVKQRELTFKAFLQNLKSVV-ASRKEAEEE
280	ref|XP_006891533.1|	98.7%	61.0%	QYFE-DSHVKEAKQCIREPRFVEVLQQNNTLSDKFVIEVDVIPKSSVCKMKSFYISMQNDK-DG--TWRQNKDHSLFVRDGASSKDIL-ANVKQREAEFKAYLLNLESIV-AARKEAEEE
281	ref|XP_038412756.1|	98.7%	60.6%	QYFE-ESEINEVKKCIREPRFVEVLLQNNTLSDRFVIEVDVIPKHSVCEKKYFLIRMQNCK-SE--TWKPNQDLSLFVRDGPSSKDIL-ANVKQRELNLKAFLQNLTSVV-ASRKEAEEE
282	ref|XP_025327152.1|	98.7%	60.6%	QYFE-ESEINEVKKCIREPRFVEVLLQNNTLSDRFVIEVDVIPKHSVCEKKYFLIRMQNCK-SE--TWKPNQDLSLFVRDGPSSKDIL-ANVKQRELNLKAFLQNLTSVV-ASRKEAEEE
283	ref|XP_038542377.1|	98.7%	60.6%	QYFE-ESEINEVKKCIREPRFVEVLLQNNTLSDRFVIEVDVIPKHSVCEKKYFLIRMQNCK-SE--TWKPNQDLSLFVRDGPSSKDIL-ANVKQRELNLKAFLQNLTSVV-ASRKEAEEE
284	ref|XP_012881542.1|	98.7%	62.3%	KYFE-ESEIQEAKKCIREPRFVEVLLQNNTPSDRFVIEVDIIPKHSVCKEKYFYIKMQNNA-DK--TWKQDQDHSLYVREGASSKNIL-ASVKKRDTEFKAFLENLKSWI-ASRKEAEEE
285	ref|XP_055163576.1|	98.7%	60.2%	QYFE-ESEINEVKKCIREPRFVEVLLQNNTLSDRFVIEVDVIPKHSVCEKKYFLIKMQNCK-SE--TWKPNQDLSLFVRDGPSSKDIL-ANAKQRELNLKAFLQNLKSVV-ASRKEAEEE
286	ref|XP_054582508.1|	98.7%	62.9%	QYFE-ESEINEAKKCIREPRFVEVLLQNNTPSDRFVIEVDVIPKHSVCKEKYFYIKMQNCK-AD--IWKQNQDASLFVRDGASSKDIL-ANVKQRDKDFKAFTNEVKSLA-VSRKEAEEE
287	ref|XP_006733000.1|	98.7%	61.4%	HYFE-ESEINEVKKCIREPRFVEVLLQNNTPSDRFVIEVDVIPKHSVCEEKYFLIKMQSCK-NE--TWKPNQDFSLFVRDGPSSMDVL-ANVKQRDVAYKNFLQNLKTVI-ASRKVAEEE
288	ref|XP_060488440.1|	98.3%	62.3%	QYFE--DDVNEAKKCIREPRFVEVLLQNNTPSDRFVIEVDVIPKHSVCEEKYFLIRMQNCK-NE--TWKPNQDPSLFVRDGASSKDIL-ANVKQRDIAYKAFLQNLKSLA-ASRKEAEEE
289	ref|XP_019298861.2|	98.3%	62.3%	QYFE--DDVNEAKKCIREPRFVEVLLQNNTPSDRFVIEVDVIPKHSVCEEKYFLIRMQNCK-NE--TWKPNQDPSLFVRDGASSKDIL-ANVKQRDIAYKAFLQNLKSLA-ASRKEAEEE
290	ref|XP_042773716.1|	98.3%	62.3%	QYFE--DDVNEAKKCIREPRFVEVLLQNNTPSDRFVIEVDVIPKHSVCEEKYFLIRMQNCK-NE--TWKPNQDPSLFVRDGASSKDIL-ANVKQRDIAYKAFLQNLKSLA-ASRKEAEEE
291	ref|XP_032731004.1|	98.7%	61.9%	QYFE-GNEINEAKKCIREPRFVEVLLQNNTPSDRFVIEVDVIPKHSVCEEKYFLVKMQNCK-NE--TWKPNQDPSLFVRDGPSSKDIL-ANVKQRERVYKEFLQNLKSVV-ASRKEAEEE
292	ref|XP_006163552.1|	98.7%	61.9%	KYFE-ESEINEAKKCIRQPRFVEVLLQNNMPSDRCVIEVDIIPKHSVCKRKYFYIKMQSCQ-NE--TWKQNPNHSLFIREGASSKDIL-ANVKQRDAEFKAFTENLKALV-ASRKEAEEE
293	ref|XP_059568319.1|	97.0%	62.4%	QYFE-ESGINEAKKCIREPRFVEVLLQNNTPSDRFVIEVDVIPKHSVCKEKYFYIKMQSCK-AE--IWKKNKDASLFVREGASTTDIL-ANVK----GFRAFTNKVKSLA-VLRKEAEEE
294	ref|XP_045876181.1|	98.7%	61.4%	QYFE-GNEINEAKKCIREPRFVEVLLQNNTPSDRFVIEVDVIPKHSVCEEKYFLIRMQNCK-NE--TWKPNQDHSLFVRDGPSSKDIL-ANVKQRERVYKEFLQNLKSVV-ASRKEAEEE
295	ref|XP_022359131.1|	98.7%	61.9%	QYFE-ENEINEAKKCIREPRFVEVLLQNNTPSDRFVIEVDVIPKHSVCEEKYFLIRMQNCK-NE--TWKPNQDPSLFVRDGPSSKDIL-ANVKQRERVYKEFLQNLKSVV-ASRKEAEEE
296	ref|XP_005882177.1|	98.7%	62.9%	KYFK-ESEINEAKKCIREPRFVEVLLQNNTPSDRFVIEVDVIPKHSVCKEKYFCIKMQNCK-DE--IWKQNQDASLFVRDGASSKDIL-ANVKQRDKDFKAFTNKVKSLA-VSRKEAEEE
297	ref|XP_007522555.1|	98.7%	61.9%	QYFE-ESEIKEAKKCIREPRFVEVLLQNYTPSDRFVIEVDVIPKHSICKEKYFYTNMQSYK-ND--TWKQNQESSLFVRDGASSVDIL-ANGKQRDKNFKTFLQNLKSLA-EDRKDAEKE
298	ref|XP_037363842.1|	98.7%	61.4%	KYFE-ESEIKEAKKCIRQPRFVEVLLQNNTPSDRFVIEVDVIPKHSVCKEKYFYIKMQTLI-NE--TWKQSQDTSLYVREGASSKDIL-ADDKKRDGNFKKFHKNLKLLV-ALRKEAEEE
299	ref|XP_036614332.1|	98.7%	60.8%	HYFDYDEQVNIARKCIREPRFVEVLRQNNTLSDRFVIEVDVIPQYSICKNTLFRIRMQNCK-EK--RWKENNEYSVFVREGASSKDIL-ANVKQNDANYKKFLLDLDSLA-NSRKSAEKE
300	ref|XP_023400184.2|	98.7%	61.4%	QYFE-DSDIKEAEKCIRQPRFVEVLQQNNTPSDKFVIEVDVIPRHSVCKDKYFYINMQICK-DG--SWRQDKDYSLFVRYGASSKDIL-ANVRQRDADFKAFTLNFESVV-ASRKEAEEE
301	ref|XP_003407194.2|	98.7%	61.4%	QYFE-DSDIKEAEKCIRQPRFVEVLQQNNTPSDKFVIEVDVIPRHSVCKDKYFYINMQICK-DG--SWRQDKDYSLFVRYGASSKDIL-ANVRQRDADFKAFTLNFESVV-ASRKEAEEE
302	ref|XP_044539777.1|	98.7%	61.0%	HYFD-DNHVNKARKCIREPRFVEVLLQDSTPSDRFVIEVDVIPLYSICKKYYFAIKRQVLK-DK--KWKLNNEYELFVREGASSKDIF-ANIKQNDANFKMFLLGLETLA-SSRKSAEEE
303	ref|XP_036735125.2|	98.7%	58.9%	QYFE-ESEINEAKKCVREPRFVDVLLQNNTPSDRFVIEVDVIPKHSICKEKYFYIKLQNCK-SE--SWKPNQDHSLFVRDGASSKDIL-ANVRQRDIDFKAFIQNLKSLV-ASRKEAEKE
304	ref|XP_004646313.1|	98.7%	61.7%	KYFE-ESDISEAKKCIRQPRFVEVLLQNNTLSDRFVIEVDIIPKHSVCKQKYFYIKMQIQT-DN--IWKQSQDHSLFVRDGASSKDIL-ANPKQRDTDFKAFLENLQSCVVASRKDAEEE
305	ref|XP_023562287.1|	98.7%	61.7%	KYFE-ESDISEAKKCIRQPRFVEVLLQNNTLSDRFVIEVDIIPKHSVCKQKYFYIKMQIQT-DN--IWKQSQDHSLFVRDGASSKDIL-ANPKQRDTDFKAFLENLQSCVVASRKDAEEE
306	ref|XP_020843067.1|	98.7%	59.9%	RYFDYGEQATIARKCIREPRFVEVLLQDNTLSDRFVIEVDVIPQYSICKHTIFRIRMQNYK-DE--RWKESNEHSIFVREGASSKDIL-ANVKQNDANYRKFLLDLDSLA-NSRKSAEEE
307	ref|XP_055974091.1|	98.7%	60.6%	RYFE-DSEIKEARKCIREPRFVEVLLQNNTLSDRFVIEVDVIPKHSVCQDKYFYTKMQELE-DG--IWKQTENTSLFVREGASTKDIL-ANERTRDVAFKAFLQDLKSLM-TMRKEAEEK

```
308 ref|XP_005388084.1|  98.3%  59.9%     KYFE-ESEIHEAKQCIREPRFVEVLLQNNTLSDRFVIEVDVIPKHSVCKEKYFYVKLQSET-DK--IWKQNENCSLFVRDGASTKDIL-ANVKQRNKDFKKFFENLPSCV-ASRKDAEEE
309 ref|XP_068942246.1|  98.7%  59.9%     HYFDFDEQVNMARKCIREPRFVEVLLQNNTLSDRFVIEVDVIPQYSICKNTLFRTRMHNCT-EK--GWKKNKEYSVFVREGASSKDIL-ANIKQNDANYKIFLLNLDSLA-NSRKSAEEE
310 ref|XP_029784994.1|  98.3%  61.4%     QYFE--DEVNAAKKCIREPRFVEVLLQNNMPSDRFVIEVDVIPKHSVCEEKYFLIRMQNCK-NE--TWKPNQDLSLFMRDGASSKDIL-ANAKQRDIVQKEFLQNLKSLA-ASRKEAEEE
311 ref|XP_029784955.1|  98.3%  61.4%     QYFE--DEVNAAKKCIREPRFVEVLLQNNMPSDRFVIEVDVIPKHSVCEEKYFLIRMQNCK-NE--TWKPNQDLSLFMRDGASSKDIL-ANAKQRDIVQKEFLQNLKSLA-ASRKEAEEE
312 ref|XP_007938002.1|  98.7%  59.7%     QYFE-ESDIKEARKCIREPRFVQVLQQNSTLSDRFVIEVDVIPRHSVCKKKYFYIKMQICK-DS--VWRQDKEYSLFVRKGATTKDIL-SNVRQKDADFKAFLLDLDSLV-ASRREAEEE
313 ref|XP_059112478.1|  98.7%  61.4%     KYFE-DSDIKEARACIREPRFVEVLLQNNTSSDRFVIEVDVIPKHSICREKYFYIMMQSYT-DK--TWKQSKENSLFVREGASSKNIL-ANPKQRDREFKKFLENIKTWA-ASRKEAEE-
314 ref|XP_052577148.1|  98.7%  61.0%     KYFE-DSDIKEARACIREPRFVEVLLQNNTTSDRFVIEVDVIPKHSICKEKYFYIMMQSYT-DK--TWKQSKENSLFVREGASSKNIL-ANPKQRDREFKKFLENIRTWT-ASRKEAEE-
315 ref|XP_006779273.1|  98.7%  62.9%     QYFE-ESEINEAKKCIREPRFVEVLLQNNTPSDRFVIEVDVIPKHSVCKEKYFYIKMQNCK-AE--IWKQNQDASLFVRDGASSKDIL-ANVKQRDKDFKAFTKKVKSLA-VSRKEAEEE
316 ref|XP_028725615.1|  98.7%  61.0%     KYFE-DSVIKEARACIREPRFVEVLLQNNTSSDRFVIEVDVIPKHSICKENYFYITMQSYT-DK--TWKQSKENSLFVREGASSKNIL-ANPKQWDREFRKFLENLKTWT-ASRKEAEE-
317 ref|XP_055002314.1|  98.7%  60.6%     HYFE-DSEIKEARKCIREPRFVEVLLQNNTSSDRFVIEVDVIPKHSICQEKYFYTKMQELK-DG--IWKQTENTSLFVREGASTKDIL-ANERTRDAGFKAFLQDLKSLI-TMRKEAEEK
318 ref|XP_059112476.1|  98.7%  60.6%     KYFE-DSDIKEARACIREPRFVEVLLQNNTSSDRFVIEVDVIPKHSICREKYFYIRMQSYT-DK--TWKQNKENSLFVREGASSKNIL-ANPKQQDRELKKFLENIKTRA-ASRKEAEE-
319 ref|XP_004702866.1|  98.7%  59.7%     QYFE-ESHIKEAKKCIREPRFVEVLQQNNTLSDKFVIEVDVIPRHSVCKEKYFYITMQNCK-DG--PWRPNKEHSLFVREGASSKDVL-ANIKQRDADFNAFMRNLKSLA-ACRREAEEE
320 ref|XP_048195987.1|  98.7%  60.6%     KYFE-ESDIKVAKKCIREPRFVEVLLQNNTPSDRFVIEVDIIPKHSVCKEKYFYIKVQSKT-DE--TWKQDQDHSLYVREGATSTNIL-AKAKTRDINFKTFLENLNSLA-VSRKEAEEE
321 ref|XP_028725619.1|  98.7%  61.0%     KYFE-DSDIKEARACIRGPRFVEVLLQNNTSSDRFVIEVDVIPKHSICKEKYFYIMMQSYT-DK--TWKQSKENSLFVREGASSKNIL-ANPKQRDREFRKFLENLKTWT-ASRKEAEE-
322 ref|XP_044101964.1|  98.7%  61.9%     QYFE-GNEVNEAKKCIREPRFVEVLLQNNTPSDRFVIEVDVIPKHSVCEEKYFLIRMQNCK-NE--TWKPNQDLSLFVRDGPSSKDIL-ANVKQRERVYKEFLQNLKSVI-ASRKEAEEE
323 ref|XP_035307523.1|  98.7%  59.7%     KYFE-DSDIKEARECIREPRFVEVLLQNNTPSDRFVIEVDVIPKHSICKEKYFYIIMQSYT-DK--TWKQSKENCLFVREGTSSKNIL-ANAKQRDREFRKFLENLKAWT-ASRKEAEE-
324 ref|XP_036285317.1|  98.7%  60.0%     HYFE-ESDINEAKKCIREPRFVEVLLQNNTPSDRFVIEVDVIPKHSVCKEKYFYITMQNCK-EE--IWKQNQEASLFVRDGARSVDIL-ANVKQRDKVFKAFTNDVKSLA-VSRKEAEEE
325 ref|XP_036285315.1|  98.7%  60.0%     HYFE-ESDINEAKKCIREPRFVEVLLQNNTPSDRFVIEVDVIPKHSVCKEKYFYITMQNCK-EE--IWKQNQEASLFVRDGARSVDIL-ANVKQRDKVFKAFTNDVKSLA-VSRKEAEEE
326 ref|XP_045434389.1|  98.7%  60.0%     HYFE-ESDINEAKKCIREPRFVEVLLQNNTPSDRFVIEVDVIPKHSVCKEKYFYITMQNCK-EE--IWKQNQEASLFVRDGARSVDIL-ANVKQRDKVFKAFTNDVKSLA-VSRKEAEEE
327 ref|XP_006991269.2|  98.7%  60.6%     KYFE-DSDIKEARACIREPRFVEVLLQNNTSSDRFVIEVDVIPKHSICKEKYFYIMMQSNT-DK--TWKQSKENSLFVREGASSKNIL-ANPKQRDREFKKFQENLKTWT-ASRKEAEE-
328 ref|XP_021508901.1|  98.7%  60.3%     KYFE-DSDIKEARACIREPRFVEVLLHNNTPSNRFVIEVDVIPKHSICKEKYFYIMMQSCT-DK--TWKQSKDSSLFVREGASSKNIL-ANVKQRDREFKKFLENLKTWT-KSRKEAEEE
329 ref|XP_035294151.1|  98.7%  59.3%     KYFE-DSDIKEARECIREPRFVEVLLQNNTPSDRFVIEVDVIPKHSICKEKYFYIIMQSYT-DK--TWKQSKENCLFVREGTSSKNIL-ANAKQRDREFRKFLENLKAWT-ASRKEAEE-
330 ref|XP_042128701.1|  98.7%  60.2%     KYFE-DSDIKEARACIREPRFVEVLLQNNTSSDRFVIEVDVIPKHSICKEKYFYIMMQSCT-DK--TWKQSKEISLFVREGASSKNIL-ANPKQRDREFRKFLENLKTWT-ASRKEAEE-
331 ref|XP_006991267.2|  98.7%  60.2%     KYFE-DSDIKEARACIREPRFVEVLLQNNTSSDRFVIEVDVIPKHSICKEKYFYIMMQSCT-DK--TWKQSKEISLFVREGASSKNIL-ANPKQRDREFRKFLENLKTWT-ASRKEAEE-
332 ref|XP_003771994.1|  98.7%  59.7%     QYFR-DDQVNLARECIREPRFVEVLLQDNTLSDRFVIEVDVIPKYSICKNTLFYIKMQVLK-EE--KWMEKKEYSLFVREGASSKDIL-ANIMQRHVDYNRFLVHLNSLA-DSRKSAEEE
333 ref|XP_039111369.1|  98.3%  60.2%     QYFE--AEVNVAKSCIREPRFVDVLLQNNTPSDRFVIEVDVIPKHSVCEEKYFLIRMQSCK-NE--TWKPNQDLSLFVRDGASSKDIL-ANVKQRDIAYKEFLQNLKSLT-ASRKEAEEE
334 ref|XP_021010545.1|  98.3%  59.5%     KYFE-DSEISEARACIREPRFVEVLLQNNTQSNRFVIEVDVIPRHSICQEKYFYIMMQSNT-GK--TWKQSKDTSLFVREGASSKNIL-GNPNQRDREFKKFLEDLKMWT-ASRKAAEEE
335 ref|NP_034286.2|     98.3%  59.5%     KYFE-DSEISEARACIREPRFVEVLLQNNTQSNRFVIEVDVIPRHSICQEKYFYIMMQSST-GK--TWKQSKDTSLFVREGASSKNIL-GNPNQRDREFKKFLEDLKMWT-ASRKAAEEE
336 ref|XP_034374653.1|  98.7%  59.5%     KYFE-DSEISKARACIREPRFVEVLLQNNTQSNRFVIEVDVIPKHSICQEKYFNIMMQSST-GK--SWKQSKDTSLFVREGASSKNIL-GNPNQRERKFKKFLEDLKSWT-ASRKAAEEE
337 ref|XP_051857307.1|  98.7%  58.9%     QYFR-DDQVNLARECIREPRFVEVLLQDNTLSDRFVIEVDVIPKYSICKNALFNIKMQILK-EE--KWMEKKEYSLFVREGASSKDIL-ANIMQRDADYNSFLVNLNSLV-DSRKSAEEE
338 ref|XP_048292669.1|  98.3%  59.7%     RYFE-DSDIKEARACIREPRFVEVLLQNNTSSDRFVIEVDVIPKHSICKEKYFYIMMQSYT-DK--TWKQSTENSLFVREGASSKNIL-ANAKQRDREFKNFLENLKSWT-ASRKEAEE-
339 ref|XP_051007938.1|  98.7%  58.6%     KYFE-DSDIKEARACIREPRFVEVLLQNSMPSNRFVIEVDVIPKHSICKEKYFYIMMQSYA-DK--TWKQSKDLSLFVREGASSKNIL-ANAKQRDREFKQFLEKLKMWA-GSRKEAEEE
340 ref|XP_036038446.1|  98.7%  59.3%     KYFD-DSDIKEARVCIREPRFVEVLMQNNTSADRFVIEVDVIPKHSICKEKYFYIMMQSYT-DK--TWKQSKENSLFVREGASSKNIL-ANPKQRDREFKKFLENLKTWT-ASRKEAEE-
341 ref|XP_036038795.1|  98.7%  58.1%     KYFD-DSDIKEARACIREPRFVEVLMQNNTSADRFVIEVDVIPKHSICEEKYFYIRMQSYT-DK--TWKQSEENSLFVREGASSKNIL-ANPKQRDREFKKFLENLKTWT-ASRKEAEE-
342 ref|XP_034375667.1|  98.7%  58.6%     KYFE-EGQIREARACIREPRFVEVLLQNTTQSNRFVIEVDVIPNHSICHEKYFNIMMQSST-GK--SWKQSKDTSLFIREGASSRNIL-GNPNQRDREFKKFLEDLKTWT-ASRKAAEEQ
343 ref|XP_049985914.1|  98.3%  60.2%     QYFE-DSDIKEARACIREPRFVEVLLQNNTSSDRFVIEVDVIPKHSICKDKYFYITMQSYT-DK--TWKQSTENSLFVREGASSKNIL-ANAKQRDREFKNFLENLKLWT-ASRKEAEE-
344 ref|XP_055468922.1|  98.7%  59.5%     KYFE-DSDIKEARACIREPRFVEVLLQNNTPSNRFVIEVDVIPKHSICKEKSFYIMMQSCT-DK--TWKQSKDSSLFVREGASSKNIL-ANVKQRDREFKKFLENLKTWT-KSRKEAEEE
345 ref|XP_016281899.2|  98.7%  57.6%     HYFD-DNHVNKARKCIREPRFVEVLLQDNTSSDRCVIEVDVIPLYSICKEFYFSIKQQVLK-DG--KWKLNDEYGLFVREGASTKDIL-ANVRQNDAKYKMFLLELQTLA-SSRKSAEEE
346 ref|XP_040597137.1|  98.3%  59.7%     KYFE-DSDIKEARACIREPRFVEVLLQNNTQSDRFVIEVDVIPKHSICKEKYFYVMMQSYT-DK--TWKKSQENSLFVREGPSSKNIL-ANVNQRDREFRKFLENLKTWI-ASRKEAEE-
347 ref|XP_027703736.1|  98.7%  57.8%     RYFDYDEQATIACKCIREPRFVEVLLQDNTLSDRFVIEVDVIPQYSICKNIIFRIRMQNCK-EE--RWKERNEHSIFVREGASSKDIL-ANVKQNDADYQQLLLNLNSLV-KSRKSAEEE
348 ref|XP_041531603.1|  98.3%  59.7%     KYFE-DSDIKEARACIREPRFVEVLLQNKTSSDRFVIEVDVIPKHSICKEKYFYITLQSYT-DK--TWKQSTENSLFVREGASSKNIL-ANAKQRDREFKNFLENLKLWI-ASRKEAEE-
349 ref|XP_028635008.1|  98.7%  58.2%     KYFE-DSKIREARACIREPRFVEVLLQNNTQSNRFVIEVDVIPKHSICQEKYFYIMMQSET-GK--TWKQSKDTSLFVREGASSRNIL-GNPKERDREFTKFLEDLKTWI-ASRKAAEEE
350 ref|XP_006834357.1|  98.7%  57.6%     KYFE-KSHIKEAKKCIREPRFVEVLQQNNTLSDKFVIEVDVIPSHYVCKEKHFCVTMQNCK-DG--SWRPNKEHSLFVRDGAGCKDIL-ANVKQQDADFNEFIRNINSMV-ALRKEAEEE
351 ref|XP_038176710.1|  98.3%  59.7%     KYFE-DSDIKEVRACIREPRFVEVLLQNNTSSDRFVIEVDVIPKHSICKDKYFYIMMQSYA-DK--TWKQSTENSLFVREGASSKNIL-ANAKQRDREFKNFLENLKSWI-ASRKEAEE-
352 ref|XP_051033136.1|  98.3%  58.5%     KYFE-DSDIKEARACIREPRFVEVLLQNNTPSDRFVIEVDVIPKHSICKEKYFYIMMQSYT-DN--TWKPSKEVALFVREGPSSKNIL-ANAKQRDREFLKFQENLKTWT-ESRKEAEE-
353 ref|XP_052029158.1|  98.7%  57.4%     KYFE-DSVIREARACIREPRFVEVLLQNNTQSNRFVIEVDVIPKHSLCQDKYFYTMMQSYT-GT--TWKQNKDTSLFVRQGASSRDIL-SNPKQRDREFKKFLEDLKTWT-ASRKAAEEE
354 ref|XP_021047211.1|  98.7%  58.2%     KYFE-DSEISEARACIREPRFVEVLLKNSAQSNRFVIEVDVIPKHSICQEKYFYTMMQSCT-GG--TWKQSNDTSLFVREGASSRNIL-GNPKQRDREFKKFLEDLKTRT-ASRKAAEEE
355 ref|XP_057645554.1|  98.3%  59.3%     KYFD-DSDIKEARACIREPRFVEVLLQNKTPSDRFVIEVDVIPKHSICKEKYFYIMMQSYT-DK--TWKQSTENSLFVREGASSKNIL-ANAKQRDREFKNFLENLKLWI-ASRKEAEE-
356 ref|XP_020843064.1|  97.4%  58.7%     DYFG-KDGIEEAKKCIRPPRFVEVLLKNNITSDRFVIEVDIIPKHSICEKKYFQIKMQTCKENR---WEKNTEFSLFVRDGTSSKDIY-ANSKQKDGHLKIFLSGLESQD-ESRKAAESY
357 ref|XP_027703686.1|  97.4%  57.9%     DYFG-KNGVGEAKQCIRPPRFVEVLLKNNVTSDRFVIEVDIIPKHSICEKKYFPIKMQTCKENR---WEKNTEFSLYVRDGTSSKDIY-ANTKQKDEHLKAFLLDLESQD-ESRKAAESY
358 ref|XP_001378512.2|  97.0%  56.6%     VYFE-KDDVSQAKQCIRPPRFVEVLLKNNIVSDRFVIEIDIVPKHSICENKYFQVKMQIFKENK---WGKNAELSLYVRDGTSSKNIY-ANPKQKDGNIKAFLAELEKCV-KLRKEAEDH
359 ref|XP_036617339.1|  97.4%  57.9%     DYFD-KEGVGEAKQCIRPPRFVEVLLKNDTTSERFVIEVDIIPKHSICNKKYFPIKMQTYKKNR---WEKNTELSLFVRDGTSSKDIY-ANPKQKDGNLKAFLSGLESQD-ESRKAAEDY
360 ref|XP_043821246.1|  97.4%  57.4%     DYFG-KEDVREAKQCIRPPRFVEVLLKNNTTSDRFVIEVDIIPKHSICEKKYFPIKMQTCKGNK---WEKATEFSLFVRDGASSKDIY-ANPKQKDGHFKVFLSNLESLD-KSRKAEEDF
361 ref|XP_008820771.1|  98.7%  57.4%     KYFE-KDDINEAKECIREPRFVEVLLQNNTLSNRFVIEVDVIPKHSICKEKYFQVNMQGYT-AK--TQKQDQNPSLFVREGATTPDIL-ANVKQRDRNKRKFQEKLKSSI-TLRKEAEEE
362 ref|XP_044534687.1|  97.0%  54.9%     VCFD-KDNIHQAKQCIRQPRFVEVLLKNNVVSDRFVIEVDIVPKHSICENKYFQVKLQSFKENR---WEKNTELSLFVRDKTSSNDIY-ANPKQKDGNFKAFLADLEIWV-KLRKEADNY
363 ref|NP_001406829.1|  98.3%  55.9%     KYFE-DSDIHEARACIREPRFVEVLLLNNTQSNRFVIEVDVIPKHSICQEKYFYVMLQTCT-GT--TWKQSKDTSLFVREGASSRDIL-GNPKQRDREFKKFLENLKMSI-ASRKAAEE-
364 ref|XP_032762875.1|  98.3%  55.9%     KYFE-DSDIHEARACIREPRFVEVLLLNNTQSNRFVIEVDVIPKHSICQEKYFYVMLQTCT-GT--TWKQSKDTSLFVREGASSRDIL-GNPKQRDREFKKFLDNLKMSI-ASRKAAEE-
365 ref|XP_003771993.1|  97.4%  57.0%     DYFS-KEEVEEAKQCIRPPRFVEVLLKNNIPSDRFVIEVDIIPKHSICVKKYFPIKMQTFENNK---WEKNAEFSLFVRDGASSKDIY-ANAKQKDRQLKEFLTNLESWD-ESRKAAEDY
366 ref|XP_068942244.1|  97.4%  56.2%     DYFG-KERVGEAKQCIRPPRFVEVLLKNDITSDRFVIEVDIVPKHSICEKKYFPIKMQTCKENR---WEKNTEFSLFVRDGASSKDIY-ANPKQKDGNLNAFLSGLESQD-ELRKAAEDN
367 ref|XP_051856775.1|  97.4%  57.4%     DYFG-KEGVEEAKECIRPPRFVEVLLKNNIASDRFVIEVDIIPKHSICVKKYFPIKMQTLENNK---WEKNAEFSLFLRDGASSKDIY-ANAKQKDRQLKEFLTNLDSRD-ESRKAAEDY
368 ref|XP_038964512.2|  97.8%  55.5%     KYFE-DSDIHEARACIREPRFVEVLLLNNTQSNRFVIEVDVIPKHSICQEKYFYVMLQTCT-GT--TWKQSKDTSLFVREGASSRDIL-GNPKQRDREFKKFLENLKMSI-ASRKAAEE-
369 ref|XP_027245644.1|  96.1%  55.9%     KYFE-DSDIKEARECIREPRFVEVLLQNNTPSDRFVIEVDVIPKHSICKEKYFYIMMQSYT-DE--T---CKENCLFVREGASSKNIL-AKANQRDREFRKFLENLKTRI-ASRKEAEE-
370 ref|XP_003496999.1|  96.1%  55.9%     KYFE-DSDIKEARECIREPRFVEVLLQNNTPSDRFVIEVDVIPKHSICKEKYFYIMMQSYT-DE--T---CKENCLFVREGASSKNIL-AKANQRDREFRKFLENLKTRI-ASRKEAEE-
    consensus/100%                    .hFt..t.h..sp.slp.PRFl.VL..s...st+hVlElDh......s....F.h.........................................................
    consensus/90%                     pYFE..pplppA+pCIREPRFVEVLh.Nsh.SsRFVIEVDllPpaS.Ct.cYF.IpMQ.hp.pp..hWp.s.p.SlFVR-Gspo+sIh.....ppph.aptFh.slcths.tsRKtAE-.
    consensus/80%                     pYFE.ppplppAKpCIREPRFVEVLL.Nso.SDRFVIEVDllP+aS.CcccYF.IpMQshp.sp..hWcps.chSlFVR-GsSSKDIh.....pppht F+tFh.sLKpls.tSRKEAEE.
    consensus/70%                     pYFE.-pplpcAKpCIREPRFVEVLL.NsTsSDRFVIEVDVIPKaS.CcccYF.I+MQshp.sc..sWcQspchSlFVR-GASSKDIh.....ppchcFKtFh.sLKsls.tSRKEAEE.

                                cov    pid    379  385
  1 ref|NP_001180236.1| 100.0% 100.0%     -KFRAKTN
  2 ref|XP_001167273.1| 100.0%  99.1%     -KFRAKTN
  3 ref|XP_004045789.1| 100.0%  98.7%     -KFRAKTN
  4 ref|XP_055128666.1| 100.0%  97.4%     -KFRAKTN
  5 ref|XP_054352670.1| 100.0%  96.5%     -KFRAKTN
  6 ref|XP_001090497.2| 100.0%  95.7%     -KFRAKTN
  7 ref|XP_011853659.1| 100.0%  95.7%     -KFRAKTN
  8 ref|XP_003896375.1| 100.0%  95.7%     -KFRAKTN
  9 ref|XP_011887444.1| 100.0%  95.7%     -KFRAKTN
 10 ref|XP_025235592.1| 100.0%  95.7%     -KFRAKTN
 11 ref|XP_005550278.3| 100.0%  95.2%     -KFRAKTN
 12 ref|XP_002818257.3| 100.0%  96.1%     -KFRAKTN
 13 ref|XP_007980346.2| 100.0%  94.8%     -KFRAKTN
 14 ref|XP_012502835.1| 100.0%  87.4%     -KFRVKTN
 15 ref|XP_069318867.1| 100.0%  86.5%     -KFRVKTN
 16 ref|XP_008570528.1| 100.0%  85.2%     -KCRVKTN
 17 ref|XP_062957119.1| 100.0%  84.3%     -KCRVKTS
 18 ref|XP_014643658.1| 100.0%  83.9%     -KYRVKTN
 19 ref|XP_006155597.1| 100.0%  83.9%     -KLRVKSN
 20 ref|XP_058381579.1| 100.0%  83.5%     -RYRVKTN
 21 ref|XP_058381571.1| 100.0%  83.5%     -RYRVKTN
 22 ref|XP_045420643.1| 100.0%  83.9%     -KFRMKTN
 23 ref|XP_070280365.1| 100.0%  83.0%     -KCRIKTN
 24 ref|XP_006108753.1| 100.0%  83.0%     -KCRIKTN
 25 ref|XP_014403134.1| 100.0%  82.6%     -KCRIKTN
 26 ref|XP_036184081.1| 100.0%  82.6%     -KCRRKTN
 27 ref|XP_014403133.1| 100.0%  82.6%     -KCRIKTN
 28 ref|XP_036184079.1| 100.0%  82.6%     -KCRRKTN
 29 ref|XP_036184078.1| 100.0%  82.6%     -KCRRKTN
 30 ref|XP_005876148.1| 100.0%  82.6%     -KCRIKTN
 31 ref|XP_036184077.1| 100.0%  82.6%     -KCRRKTN
 32 ref|XP_059568302.1| 100.0%  82.6%     -KCRLKTN
 33 ref|XP_036285323.1| 100.0%  82.6%     -KCRIKTN
 34 ref|XP_015413823.1| 100.0%  82.6%     -KCKIKTN
 35 ref|XP_036184076.1| 100.0%  82.6%     -KCRRKTN
 36 ref|XP_006779271.1| 100.0%  82.6%     -KCKIKTN
 37 ref|XP_004676740.2| 100.0%  81.3%     -KCRVKAN
 38 ref|XP_012576483.1| 100.0%  81.3%     -KCRVKAN
 39 ref|XP_059964408.1| 100.0%  80.9%     -KCRVKTN
 40 ref|XP_014698446.3| 100.0%  80.4%     -KYRLKTS
 41 ref|XP_046526610.1| 100.0%  80.4%     -KYRLKTS
 42 ref|XP_046526611.1| 100.0%  80.4%     -KYRLKTS
 43 ref|XP_028012848.2| 100.0%  80.9%     -KCRIKTN
 44 ref|XP_019483139.1|  99.6%  80.1%     -KCRVKTN
 45 ref|XP_053409644.1|  99.6%  81.3%     -KFKGKTN
 46 ref|NP_001243854.1| 100.0%  80.0%     -KYRLKTS
```

47	ref|XP_057589207.1|	100.0%	79.6%	-KCRVKTN
48	ref|XP_040116208.1|	100.0%	80.0%	-KCRVKTN
49	ref|XP_024622799.1|	100.0%	81.3%	-KCRVKTN
50	ref|XP_024622798.1|	100.0%	81.3%	-KCRVKTN
51	ref|XP_010977976.2|	100.0%	79.6%	-KCRVKTH
52	ref|XP_024622796.1|	100.0%	81.3%	-KCRVKTN
53	ref|XP_042104307.1|	100.0%	80.0%	-KCRVKTN
54	ref|XP_006207699.1|	100.0%	79.6%	-KCRVKTH
55	ref|XP_043318526.1|	100.0%	80.0%	-KCRVKIT
56	ref|XP_020751092.2|	100.0%	80.0%	-KCRVKIT
57	ref|XP_070327721.1|	100.0%	80.0%	-KCRVKIT
58	ref|XP_022441612.1|	100.0%	80.4%	-KCRVKTN
59	ref|XP_029089619.1|	100.0%	80.4%	-KCRVKTN
60	ref|XP_022441604.1|	100.0%	80.4%	-KCRVKTN
61	ref|XP_043318525.1|	100.0%	80.0%	-KCRVKIT
62	ref|XP_070327713.1|	100.0%	80.0%	-KCRVKIT
63	ref|XP_061020880.1|	100.0%	79.6%	-KCRVKIT
64	ref|XP_022441597.1|	100.0%	80.4%	-KCRVKTN
65	ref|XP_069443508.1|	100.0%	79.6%	-KCRVKTN
66	ref|XP_010971009.1|	100.0%	79.1%	-KCRVKTH
67	ref|XP_067602509.1|	100.0%	80.4%	-KCRVKTN
68	ref|XP_005667662.1|	100.0%	78.7%	-KCRLKTN
69	ref|XP_005678975.2|	100.0%	79.6%	-KCRVKTN
70	ref|XP_023363488.1|	99.6%	80.4%	-KFKGKIN
71	ref|XP_020958041.1|	100.0%	78.7%	-KCRLKTN
72	ref|XP_007461947.1|	100.0%	80.4%	-KCRVKTN
73	ref|XP_065794903.1|	100.0%	79.6%	-KCRVKIT
74	ref|XP_065740132.1|	100.0%	80.9%	-KCRIKTN
75	ref|XP_032499074.1|	100.0%	80.9%	-KCRIKTN
76	ref|XP_023985582.1|	100.0%	80.4%	-KCRVKTN
77	ref|XP_032499071.1|	100.0%	80.9%	-KCRIKTN
78	ref|XP_032499073.1|	100.0%	80.9%	-KCRIKTN
79	ref|XP_052495196.1|	100.0%	79.6%	-KCRVKTN
80	ref|XP_060013026.1|	100.0%	80.0%	-KCRVKTN
81	ref|XP_059876504.1|	100.0%	80.0%	-KCRVKTN
82	ref|XP_012387005.2|	100.0%	80.0%	-KCRVKTN
83	ref|XP_026958261.1|	100.0%	80.0%	-KCRVKTN
84	ref|XP_049571082.1|	100.0%	80.0%	-KCRVKTN
85	ref|XP_030713128.1|	100.0%	80.0%	-KCRVKTN
86	ref|XP_070643590.1|	100.0%	79.1%	-KCRVKTN
87	ref|XP_068406760.1|	100.0%	80.4%	-KCRVKTN
88	ref|XP_060160576.1|	100.0%	80.0%	-KCRVKTN
89	ref|XP_005902867.1|	100.0%	79.1%	-KCRVKTN
90	ref|NP_001192710.3|	100.0%	79.1%	-KCRVKTN
91	ref|XP_007195831.2|	100.0%	80.4%	-KCRVKTN
92	ref|XP_036720276.1|	100.0%	80.4%	-KCRVKTN
93	ref|XP_007195832.2|	100.0%	80.4%	-KCRVKTN
94	ref|XP_036720275.1|	100.0%	80.4%	-KCRVKTN
95	ref|XP_016016799.2|	100.0%	79.7%	GKWRVKTS
96	ref|XP_061054544.1|	100.0%	80.4%	-KCRVKTN
97	ref|XP_059789816.1|	100.0%	79.6%	-KCRVKTN
98	ref|XP_004329146.1|	100.0%	79.6%	-KCRVKTN
99	ref|XP_045684325.1|	100.0%	79.6%	-KCRVKTN
100	ref|XP_068829026.1|	100.0%	78.7%	-KCRVKIN
101	ref|XP_019782959.1|	100.0%	79.6%	-KCRVKTN
102	ref|XP_045041219.1|	100.0%	80.4%	-KCRVKTN
103	ref|XP_037363830.1|	100.0%	79.1%	-KYRVKSN
104	ref|XP_007522554.1|	99.1%	78.9%	-KSRIKPN
105	ref|XP_060052209.1|	99.1%	78.9%	-KSRIKPN
106	ref|XP_047608878.1|	100.0%	77.8%	-KCRMKTS
107	ref|XP_048195986.1|	100.0%	78.7%	-KAKQKTV
108	ref|XP_039734026.1|	100.0%	79.3%	GKCRVKTS
109	ref|XP_028381693.1|	100.0%	79.1%	-KCRVKTN
110	ref|XP_016074271.1|	100.0%	78.3%	-KCRVKTN
111	ref|XP_036992384.2|	100.0%	79.1%	-KCRVKTN
112	ref|XP_006911959.1|	100.0%	78.4%	GKCRVKTS
113	ref|XP_011361926.1|	100.0%	78.4%	GKCRVKTS
114	ref|XP_020016740.1|	100.0%	78.7%	-KAKEKPA
115	ref|XP_020016739.1|	100.0%	78.7%	-KAKEKPA
116	ref|XP_004862414.1|	98.7%	77.1%	-KCKQKPN
117	ref|XP_012934202.1|	98.7%	77.1%	-KCKQKPN
118	ref|XP_054420471.1|	99.6%	79.6%	-KCRVKTN
119	ref|XP_066210460.1|	100.0%	77.8%	-KCREKTN
120	ref|XP_013013899.2|	98.7%	75.8%	-KCKLKTN
121	ref|XP_066095023.1|	100.0%	77.4%	-KCREKTN
122	ref|XP_004646297.1|	98.7%	74.9%	-KCKQKAK
123	ref|XP_059113322.1|	100.0%	75.8%	-KCKVKPN
124	ref|XP_032944686.1|	98.7%	77.8%	-KCRVKTK
125	ref|XP_010838083.1|	99.1%	77.0%	-KCRVKTN
126	ref|XP_052575709.1|	100.0%	75.3%	-KCKVKPN
127	ref|XP_019585265.1|	98.7%	77.4%	-KCRVKTK
128	ref|XP_036037353.1|	100.0%	74.9%	-KCKVKPN
129	ref|XP_055445695.1|	99.1%	75.7%	-KCRVKTN
130	ref|XP_061269427.1|	99.1%	76.1%	-KCRVKTN
131	ref|XP_012881537.1|	100.0%	77.0%	-KAKEKTI
132	ref|XP_051033135.1|	100.0%	73.6%	-KCKEKPD
133	ref|XP_069852599.1|	100.0%	76.5%	-KAKEKTI
134	ref|XP_006078386.4|	99.1%	75.2%	-KCRVKTN
135	ref|XP_061269426.1|	99.1%	76.1%	-KCRVKTN
136	ref|XP_006078384.4|	99.1%	75.2%	-KCRVKTN
137	ref|XP_006991270.1|	100.0%	74.5%	-NCKVKAN
138	ref|XP_028725617.1|	100.0%	74.5%	-NCKVKAN
139	ref|XP_008820770.1|	100.0%	76.1%	-KCKEKPN
140	ref|XP_044802364.2|	99.1%	75.2%	-KCRVKTN
141	ref|XP_008062458.2|	100.0%	73.9%	-KHRAKTN
142	ref|XP_041531686.1|	100.0%	73.6%	-TCKVKAD
143	ref|XP_040584508.1|	100.0%	73.6%	-KYKEKPD
144	ref|XP_021045894.1|	100.0%	73.2%	-KLKVKPN
145	ref|XP_048292645.1|	100.0%	74.0%	-KCKVKPD
146	ref|XP_021045323.1|	100.0%	73.6%	-KSKVKPN
147	ref|XP_042540450.1|	100.0%	76.1%	-KAKEKTI
148	ref|XP_047418909.1|	100.0%	75.7%	-KCKEKTN
149	ref|XP_034375203.1|	100.0%	73.6%	-KSKVKLN
150	ref|XP_038176268.1|	100.0%	73.6%	-KCKVKVD
151	ref|XP_038176270.1|	100.0%	73.6%	-KCKVKVD
152	ref|XP_028634992.1|	100.0%	73.6%	-KSKVKLN
153	ref|XP_032762876.1|	100.0%	72.7%	-KSKVKSN
154	ref|XP_057634583.1|	100.0%	73.2%	-KCKVKAD
155	ref|NP_001386282.1|	100.0%	72.3%	-KSKVKSD
156	ref|XP_015362209.1|	100.0%	76.1%	-KCKEKPN
157	ref|XP_019059962.1|	98.7%	73.1%	-KCKQKTN
158	ref|XP_046311157.1|	100.0%	76.1%	-KCKEKPN
159	ref|XP_027775683.2|	100.0%	76.1%	-KCKEKPN
160	ref|XP_026247365.1|	100.0%	75.7%	-KCKEKPN
161	ref|XP_005340593.1|	100.0%	76.1%	-KCKEKPN

162	ref|XP_007652776.1|	100.0%	71.9%	-KFKEKPE
163	ref|XP_055468986.1|	100.0%	71.9%	-KCKAKPK
164	ref|XP_021508904.1|	100.0%	71.0%	-KCKAKPK
165	ref|XP_012791283.2|	100.0%	70.9%	-NFRLKTS
166	ref|XP_005388082.1|	98.7%	72.2%	-KCKQKTN
167	ref|XP_052029157.1|	100.0%	73.6%	-KFKEKPN
168	ref|XP_055974093.1|	100.0%	70.0%	-NSRLKTS
169	ref|XP_040821063.1|	99.1%	70.9%	-NSMTKT-
170	ref|XP_045016549.1|	100.0%	68.7%	-KHKKNST
171	ref|XP_051007939.1|	100.0%	69.3%	-KCKVKPS
172	ref|XP_004582357.2|	99.1%	69.1%	-NPMAKT-
173	ref|XP_062034088.1|	99.6%	68.8%	-NCTAKT-
174	ref|XP_051708952.2|	99.6%	68.0%	-NCTAKT-
175	ref|XP_012373928.2|	98.7%	73.5%	-KHRVKTN
176	ref|XP_015331500.1|	78.7%	79.0%	--------
177	ref|XP_005340592.1|	98.7%	64.4%	-HGM-KVT
178	ref|XP_015362203.1|	98.7%	64.4%	-HGL-KVT
179	ref|XP_046311151.1|	98.7%	64.4%	-HGL-KVT
180	ref|XP_050641313.1|	98.7%	64.0%	-YGM-KTM
181	ref|XP_005550273.1|	98.7%	64.4%	-YGM-KTM
182	ref|XP_014989728.2|	98.7%	64.4%	-YGM-KTM
183	ref|XP_062956902.1|	98.7%	64.8%	-HEM-KPN
184	ref|XP_027775676.2|	98.7%	64.4%	-HGL-KVT
185	ref|XP_008570541.1|	98.7%	64.4%	-HEM-KST
186	ref|XP_008259950.2|	98.7%	63.6%	-YGW-KAT
187	ref|XP_065398409.1|	98.7%	64.0%	-YGM-KTM
188	ref|XP_011729134.2|	98.7%	64.0%	-YGM-KTM
189	ref|XP_027973811.1|	98.7%	63.1%	-YEV-KAN
190	ref|XP_014643659.1|	98.7%	63.1%	-YDM-KAN
191	ref|XP_032108853.1|	98.7%	64.0%	-YRM-KAM
192	ref|XP_066094273.1|	98.3%	63.3%	-CEV-KAH
193	ref|XP_028381763.1|	98.7%	63.1%	-YEA-KAN
194	ref|XP_046526743.1|	98.7%	63.6%	-YEV-KAN
195	ref|XP_058381591.1|	98.7%	63.1%	-YDM-KAN
196	ref|XP_027429555.1|	98.7%	62.3%	-YEV-KAN
197	ref|XP_017380582.1|	98.7%	63.6%	-YRM-KAM
198	ref|XP_066210387.1|	98.3%	63.3%	-CEV-KAH
199	ref|XP_016074281.1|	98.7%	64.4%	-YKV-KTN
200	ref|NP_001244057.1|	98.7%	63.6%	-YEV-KAN
201	ref|XP_040821033.1|	98.7%	62.7%	-YTA-EST
202	ref|XP_063098771.1|	98.3%	62.0%	-HQM-KTT
203	ref|XP_007980344.1|	98.7%	64.0%	-YGM-KAM
204	ref|XP_070471752.1|	98.7%	63.6%	-YEV-KAN
205	ref|XP_017812450.3|	98.7%	63.6%	-YGM-KAM
206	ref|XP_011853657.1|	98.7%	63.6%	-YGM-KTM
207	ref|XP_026247361.1|	98.7%	63.1%	-HGM-KVT
208	ref|XP_012306232.2|	98.7%	63.1%	-YRM-KAM
209	ref|XP_024432967.2|	98.7%	62.3%	-YEA-KAD
210	ref|XP_008062456.1|	98.7%	64.4%	-YGM-KAT
211	ref|XP_020016743.1|	98.7%	61.9%	-YRM-KAP
212	ref|XP_045684040.1|	98.7%	62.3%	-YEA-KAN
213	ref|XP_011929253.1|	98.7%	63.1%	-YGM-KAM
214	ref|XP_004582358.2|	98.7%	62.3%	-YTT-EST
215	ref|XP_053511032.1|	98.7%	62.7%	-YEA-KAN
216	ref|XP_025237015.1|	98.7%	63.1%	-YGM-KAM
217	ref|XP_009451890.1|	98.7%	63.1%	-YGM-KAM
218	ref|NP_001290425.1|	98.7%	63.1%	-YGM-KAM
219	ref|XP_008960246.3|	98.7%	63.1%	-YGM-KAM
220	ref|XP_012415619.1|	98.7%	61.4%	-YEV-KAN
221	ref|XP_008684459.2|	98.7%	62.3%	-YEV-KAN
222	ref|XP_036902380.1|	98.7%	61.4%	-YEA-KAN
223	ref|XP_026361422.1|	98.7%	62.3%	-YEV-KAN
224	ref|XP_002918280.1|	98.7%	62.3%	-YEV-KAN
225	ref|XP_054351924.1|	98.7%	62.7%	-YGM-KAI
226	ref|XP_009241290.3|	98.7%	62.7%	-YGM-KAI
227	ref|XP_037692600.1|	98.7%	62.7%	-YRV-TAS
228	ref|XP_037692601.1|	98.7%	62.7%	-YRV-TAS
229	ref|XP_010343634.2|	98.7%	62.7%	-HRM-KAM
230	ref|XP_021545299.1|	98.7%	62.7%	-YEM-KAN
231	ref|XP_047418554.1|	98.7%	61.9%	-HGL-KVT
232	ref|XP_047418561.1|	98.7%	61.9%	-HGL-KVT
233	ref|XP_025728670.1|	98.7%	61.2%	-YEV-KAN
234	ref|XP_018887315.2|	98.7%	62.3%	-YGM-KAM
235	ref|XP_010601767.1|	98.3%	62.9%	-HQM-KAK
236	ref|XP_036902379.1|	98.7%	61.0%	-YEA-KAN
237	ref|XP_070280369.1|	98.7%	63.3%	-YER-KAN
238	ref|XP_032616947.1|	98.7%	62.7%	-YGM-KAM
239	ref|XP_030677948.1|	98.7%	62.7%	-YGM-KAM
240	ref|XP_036184071.1|	98.7%	63.3%	-YER-KAN
241	ref|XP_004776483.1|	98.7%	62.3%	-YEV-KAN
242	ref|XP_004389713.1|	98.7%	63.1%	-YGV-KAN
243	ref|XP_049749840.1|	98.7%	62.7%	-YGV-KAN
244	ref|XP_059252834.1|	98.7%	62.3%	-YEV-KAN
245	ref|XP_033050274.1|	98.7%	62.7%	-YGM-KAM
246	ref|XP_055128669.1|	98.7%	62.3%	-YGM-KAM
247	ref|XP_010351460.2|	98.7%	62.7%	-YGM-KAM
248	ref|XP_019585266.1|	98.7%	62.4%	-YVV-KAS
249	ref|XP_046953376.1|	98.3%	62.3%	-YEV-KAN
250	ref|XP_011361946.1|	98.7%	61.9%	-YEV-KAN
251	ref|XP_040328613.1|	98.3%	62.3%	-YEV-KAN
252	ref|XP_032160436.1|	98.7%	62.3%	-YEV-KAN
253	ref|XP_003982796.1|	98.3%	62.3%	-YEV-KAN
254	ref|XP_014922007.2|	98.3%	62.3%	-YEV-KAN
255	ref|XP_025785047.1|	98.3%	62.3%	-YEV-KAN
256	ref|XP_030163818.1|	98.3%	62.3%	-YEV-KAN
257	ref|XP_047698808.1|	98.3%	62.3%	-YEV-KAN
258	ref|XP_043444794.1|	98.3%	62.3%	-YEV-KAN
259	ref|XP_025859549.1|	98.7%	60.2%	-YEM-KAD
260	ref|XP_011810448.1|	98.7%	62.3%	-YGM-KAM
261	ref|XP_032258048.1|	98.7%	61.4%	-YVV-KAN
262	ref|XP_032944614.1|	98.7%	62.4%	-YVV-KAS
263	ref|XP_059027098.1|	98.7%	61.9%	-YEV-KAN
264	ref|XP_043821279.1|	98.7%	61.4%	-YGT-KRK
265	ref|XP_045720657.1|	98.7%	61.4%	-YEM-KAN
266	ref|XP_062034304.1|	97.8%	62.3%	-YGW-KAT
267	ref|XP_049498825.1|	98.3%	62.3%	-YEV-KAN
268	ref|XP_034876457.1|	98.7%	61.4%	-YEM-KAN
269	ref|XP_023082516.2|	98.7%	61.9%	-YGM-KAM
270	ref|XP_007076766.2|	98.3%	62.3%	-YEV-KAN
271	ref|XP_045350732.1|	98.3%	62.3%	-YEV-KAN
272	ref|XP_058581065.1|	98.3%	62.3%	-YEV-KAN
273	ref|XP_016016798.2|	98.7%	61.4%	-YEV-KAN
274	ref|XP_069852600.1|	98.7%	62.7%	-YVM-KAS
275	ref|XP_015444939.1|	98.7%	61.4%	-YEV-KAN
276	ref|XP_036875742.1|	98.7%	59.3%	-YEV-RAN

277	ref|XP_039734028.1|	98.7%	61.4%	-YEV-KAN
278	ref|XP_042540451.1|	98.7%	62.7%	-YVM-KAS
279	ref|XP_041583429.1|	98.7%	60.2%	-YEM-KAD
280	ref|XP_006891533.1|	98.7%	61.0%	-YGV-KAN
281	ref|XP_038412756.1|	98.7%	60.6%	-YEM-KAD
282	ref|XP_025327152.1|	98.7%	60.6%	-YEM-KAD
283	ref|XP_038542377.1|	98.7%	60.6%	-YEM-KAD
284	ref|XP_012881542.1|	98.7%	62.3%	-YVM-KAS
285	ref|XP_055163576.1|	98.7%	60.2%	-YEM-KAD
286	ref|XP_054582508.1|	98.7%	62.9%	-YER-KAN
287	ref|XP_006733000.1|	98.7%	61.4%	-YEM-KAN
288	ref|XP_060488440.1|	98.3%	62.3%	-YEV-KAN
289	ref|XP_019298861.2|	98.3%	62.3%	-YEV-KAN
290	ref|XP_042773716.1|	98.3%	62.3%	-YEV-KAN
291	ref|XP_032731004.1|	98.7%	61.9%	-YEV-KAN
292	ref|XP_006163552.1|	98.7%	61.9%	-YAM-KAS
293	ref|XP_059568319.1|	97.0%	62.4%	-YER-KAN
294	ref|XP_045876181.1|	98.7%	61.4%	-YEV-KAN
295	ref|XP_022359131.1|	98.7%	61.9%	-YEV-KAN
296	ref|XP_005882177.1|	98.7%	62.9%	-YER-KAN
297	ref|XP_007522555.1|	98.7%	61.9%	-HEM-KAN
298	ref|XP_037363842.1|	98.7%	61.4%	-YKM-KAN
299	ref|XP_036614332.1|	98.7%	60.8%	-YGT-KKK
300	ref|XP_023400184.2|	98.7%	61.4%	-YGV-KAN
301	ref|XP_003407194.2|	98.7%	61.4%	-YGV-KAN
302	ref|XP_044539777.1|	98.7%	61.0%	-YVT-KRK
303	ref|XP_036735125.2|	98.7%	58.9%	-YEV-RAN
304	ref|XP_004646313.1|	98.7%	61.7%	-HRM-KAT
305	ref|XP_023562287.1|	98.7%	61.7%	-HRM-KAT
306	ref|XP_020843067.1|	98.7%	59.9%	-NGT-KRK
307	ref|XP_055974091.1|	98.7%	60.6%	-YEV-KVN
308	ref|XP_005388084.1|	98.3%	59.9%	-HRM-KVT
309	ref|XP_068942246.1|	98.7%	59.9%	-YGA-KRK
310	ref|XP_029784994.1|	98.3%	61.4%	-YEM-KAS
311	ref|XP_029784955.1|	98.3%	61.4%	-YEM-KAS
312	ref|XP_007938002.1|	98.7%	59.7%	-YGV-QGN
313	ref|XP_059112478.1|	98.7%	61.4%	-EDR-MVT
314	ref|XP_052577148.1|	98.7%	61.0%	-EDR-MVT
315	ref|XP_006779273.1|	98.7%	62.9%	-YER-RAN
316	ref|XP_028725615.1|	98.7%	61.0%	-EDR-MVT
317	ref|XP_055002314.1|	98.7%	60.6%	-DEV-KVN
318	ref|XP_059112476.1|	98.7%	60.6%	-EDR-MVT
319	ref|XP_004702866.1|	98.7%	59.7%	-YGV-KAN
320	ref|XP_048195987.1|	98.7%	60.6%	-YVI-KTI
321	ref|XP_028725619.1|	98.7%	61.0%	-EDR-MVT
322	ref|XP_044101964.1|	98.7%	61.9%	-YEA-KAN
323	ref|XP_035307523.1|	98.7%	59.7%	-QQR-MVT
324	ref|XP_036285317.1|	98.7%	60.0%	-YER-MSS
325	ref|XP_036285315.1|	98.7%	60.0%	-YER-MSS
326	ref|XP_045434389.1|	98.7%	60.0%	-YER-MSS
327	ref|XP_006991269.2|	98.7%	60.6%	-EDR-MVT
328	ref|XP_021508901.1|	98.7%	60.3%	-QRR-VVV
329	ref|XP_035294151.1|	98.7%	59.3%	-QQR-MVT
330	ref|XP_042128701.1|	98.7%	60.2%	-EDR-MVT
331	ref|XP_006991267.2|	98.7%	60.2%	-EDR-MVT
332	ref|XP_003771994.1|	98.7%	59.7%	-YRT-KKE
333	ref|XP_039111369.1|	98.3%	60.2%	-YEV-KAK
334	ref|XP_021010545.1|	98.3%	59.5%	--LR-MVT
335	ref|NP_034286.2|	98.3%	59.5%	--LR-MVT
336	ref|XP_034374653.1|	98.7%	59.5%	-ELR-MVT
337	ref|XP_051857307.1|	98.7%	58.9%	-YRT-KRE
338	ref|XP_048292669.1|	98.3%	59.7%	-H-M-VVT
339	ref|XP_051007938.1|	98.7%	58.6%	-HRV-VVT
340	ref|XP_036038446.1|	98.7%	59.3%	-EDR-MVT
341	ref|XP_036038795.1|	98.7%	58.1%	-EDR-MVT
342	ref|XP_034375667.1|	98.7%	58.6%	-QLR-MVA
343	ref|XP_049985914.1|	98.3%	60.2%	-H-R-VVT
344	ref|XP_055468922.1|	98.7%	59.5%	-QHR-VVV
345	ref|XP_016281899.2|	98.7%	57.6%	-HVT-KRK
346	ref|XP_040597137.1|	98.3%	59.7%	-Q-R-MVT
347	ref|XP_027703736.1|	98.7%	57.8%	-YET-KRK
348	ref|XP_041531603.1|	98.3%	59.7%	-H-R-VVT
349	ref|XP_028635008.1|	98.7%	58.2%	-ELR-MVA
350	ref|XP_006834357.1|	98.7%	57.6%	-FGV-KAN
351	ref|XP_038176710.1|	98.3%	59.7%	-H-R-VVT
352	ref|XP_051033136.1|	98.3%	58.5%	-H-W-MVT
353	ref|XP_052029158.1|	98.7%	57.4%	-ELR-MIA
354	ref|XP_021047211.1|	98.7%	58.2%	-ELR-MVA
355	ref|XP_057645554.1|	98.3%	59.3%	-H-R-VVT
356	ref|XP_020843064.1|	97.4%	58.7%	---RPKEI
357	ref|XP_027703686.1|	97.4%	57.9%	---RPKEI
358	ref|XP_001378512.2|	97.0%	56.6%	---QPKEI
359	ref|XP_036617339.1|	97.4%	57.9%	---RPKEI
360	ref|XP_043821246.1|	97.4%	57.4%	---QPKEA
361	ref|XP_008820771.1|	98.7%	57.4%	-HRR-KVT
362	ref|XP_044534687.1|	97.0%	54.9%	---QPKEI
363	ref|NP_001406829.1|	98.3%	55.9%	--CM-VVS
364	ref|XP_032762875.1|	98.3%	55.9%	--CM-VVS
365	ref|XP_003771993.1|	97.4%	57.0%	---RPKER
366	ref|XP_068942244.1|	97.4%	56.2%	---QPKET
367	ref|XP_051856775.1|	97.4%	57.4%	---RPKER
368	ref|XP_038964512.2|	97.8%	55.5%	--CM-VVS
369	ref|XP_027245644.1|	96.1%	55.9%	-Q-R-MVT
370	ref|XP_003496999.1|	96.1%	55.9%	-Q-R-MVT
	consensus/100%			....
	consensus/90%			...h.hs.
	consensus/80%			.hth.Kss
	consensus/70%			.hth.Kss

MView 1.67, Copyright © 1997-2020 Nigel P. Brown

Figure S1C

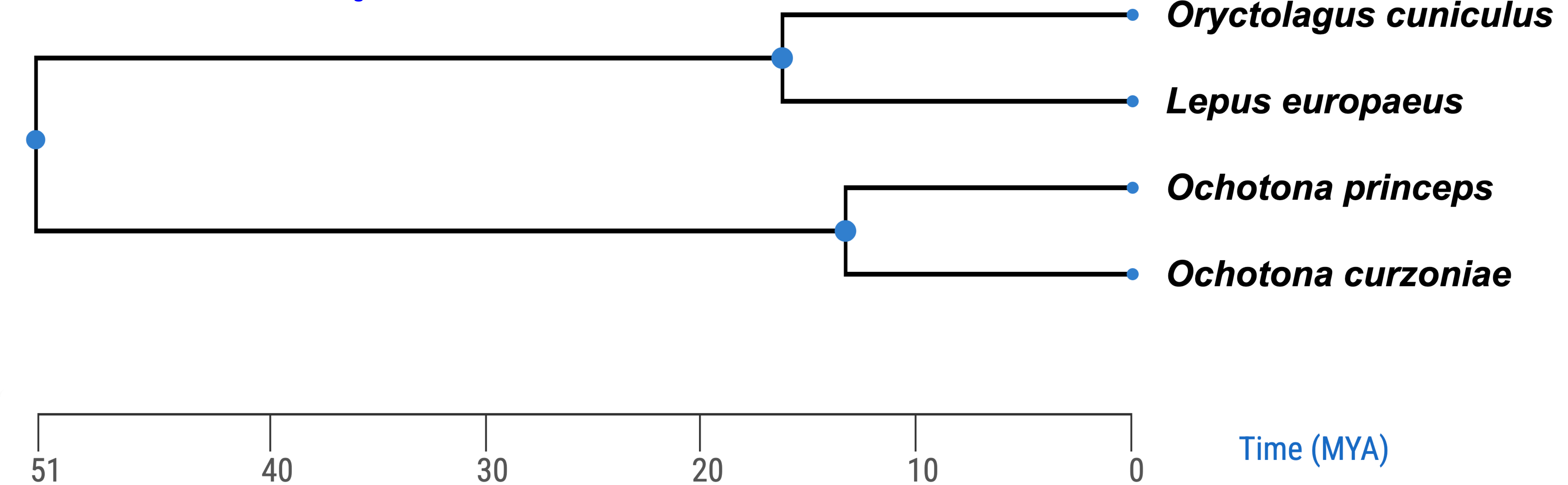

Oryctolagus cuniculus
Lepus europaeus
Ochotona princeps
Ochotona curzoniae
51
40
30
20
10
0
Time (MYA)
