# Supplementary material for: Structural and functional studies of rabbit SAMD9 reveal a distinct tRNase module that underlies the antiviral activity
Source: PLoS Pathog. 2025 Jul 31;21(7):e1013118. doi: 10.1371/journal.ppat.1013118 (PMC12331169; doi:10.1371/journal.ppat.1013118)
Supplement: S3 Fig — rSAMD9158-389 forms a stable complex with tRNAPhe and dsDNA. (A) Purified rSAMD9158-389 protein was incubated with yeast tRNAPhe and analyzed by native agarose gel electrophoresis (EMSA). The gel was first stained with ethidium bromide (EtBr, top) to visualize tRNAPhe and then with Coomassie stain (bottom) to detect protein. A stable rSAMD9158-389:tRNA complex is indicated by the red arrow. (B) rSAMD9158-389 was tested for binding with a 26 nt dsDNA at various molar ratios, analyzed by EMSA as in (A). Note that the stable complex was predominant at the 2:1 ratio. Red arrows indicate the rSAMD9158-389:dsDNA complex. (C) Purified rSAMD9158-389 proteins (WT and mutants) were tested for binding to yeast tRNAPhe. Note that the K200E and K216E mutations abolished binding. (PDF) [file ppat.1013118.s003.pdf]

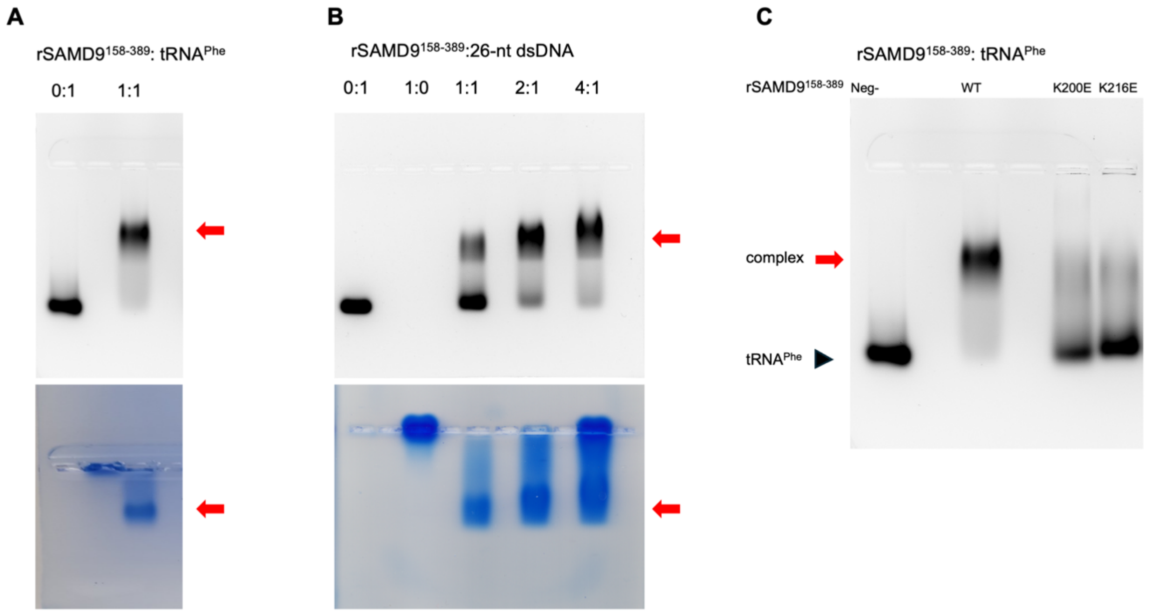
A
rSAMD9158-389: tRNAPhe
0:1
1:1
B
rSAMD9158-389:26-nt dsDNA
0:1
1:0
1:1
2:1
4:1
C
rSAMD9158-389: tRNAPhe
rSAMD9158-389
Neg-
WT
K200E
K216E
complex
tRNAPhe
