# Supplementary figures and images for: Structural and functional studies of rabbit SAMD9 reveal a distinct tRNase module that underlies the antiviral activity
Source: PLoS Pathog. 2025 Jul 31;21(7):e1013118. doi: 10.1371/journal.ppat.1013118 (PMC12331169; doi:10.1371/journal.ppat.1013118)

**A**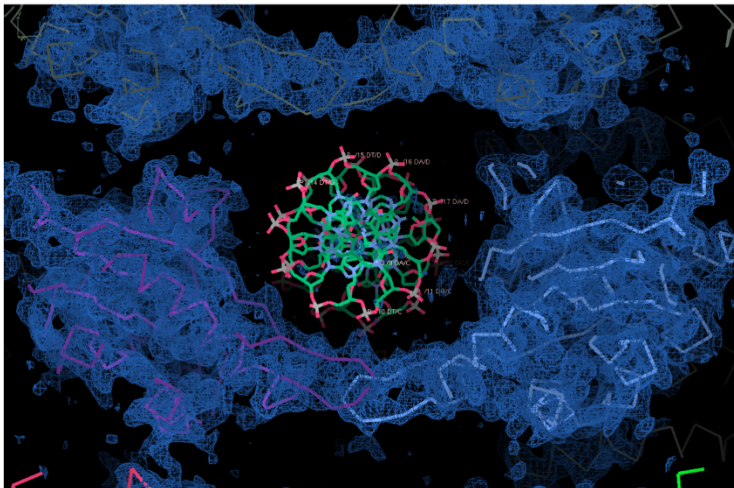**B**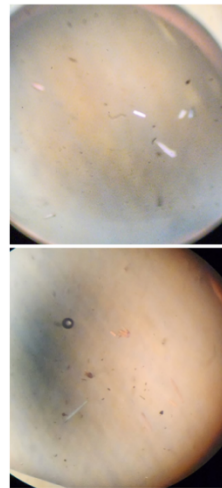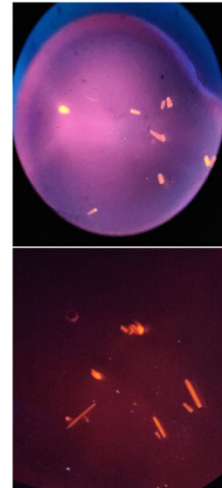

Supplement: S4 Fig — (A) The 2mFo-DFc electron density maps for protein molecules are shown in blue. The modeled dsDNA backbone is shown as sticks. Notice available space for dsDNA in crystal packing. (B) EtBr staining of the crystals, which shines strongly in red under UV light (right) indicating presence of dsDNA. (PDF) [file ppat.1013118.s004.pdf]

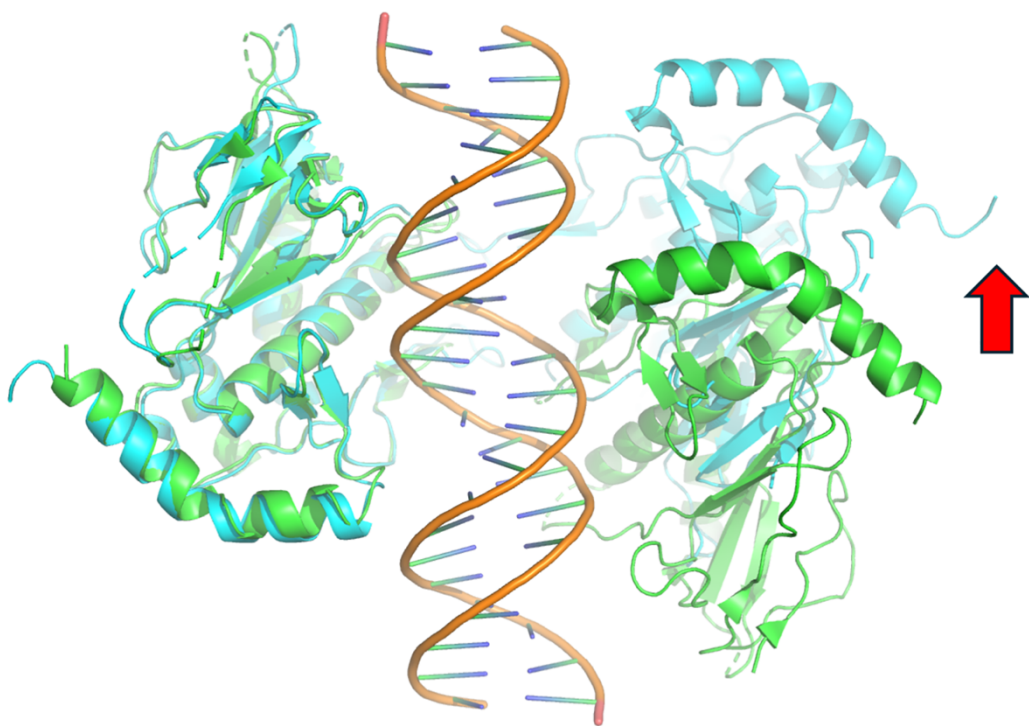

Supplement: S5 Fig — The dsDNA in the crystal structure from hSAMD9156-385:dsDNA complex is shown. Red arrow indicates the shift of the second protomer in rSAMD9158-389 structure relative to that in hSAMD9156-385 structure. (PDF) [file ppat.1013118.s005.pdf]
