# Supplementary material for: Structural and functional studies of rabbit SAMD9 reveal a distinct tRNase module that underlies the antiviral activity
Source: PLoS Pathog. 2025 Jul 31;21(7):e1013118. doi: 10.1371/journal.ppat.1013118 (PMC12331169; doi:10.1371/journal.ppat.1013118)
Supplement: S1 Table — (DOCX) [file ppat.1013118.s006.docx]

Table S1. Crystallographic data and Refinement Statistics

| Data collection | rSAMD9^158-389^ |  |
| --- | --- | --- |
| Beamline | 19-ID, APS |  |
| Wavelength, Å | 0.97918 |  |
| Space group | P2_1_2_1_2 |  |
| Cell parameters a, b, c, Å | 108.0, 142.5, 83.1 |  |
| Resolution, Å | 50.00–2.40 (2.49–2.40) |  |
| Total reflections | 322,350 |  |
| Unique reflections | 51,039 (5,002) |  |
| Redundancy | 6.3 (6.1) |  |
| Completeness, % | 99.8 (99.7) |  |
| I/σ | 15.5 (1.4) |  |
| Rsym, % | 8.1 (113.8) |  |
| CC1/2, % | 99.4 (73.8) |  |
| Refinement statistics | | |
| Resolution range used, Å | 43.5-2.40 |  |
| No. reflections used | 50,819 |  |
| *R*work/*R*free, % | 22.8/27.7 |  |
| Rmsd bond lengths, Å | 0.012 |  |
| Rmsd bond angles, ° | 1.394 |  |
| Number of atoms (average B, Å^2^) | | |
| Protein | 7,053 (64.9) |  |
| Water | 21 (30.0) |  |
| Ramachandran values | | |
| Preferred regions, % | 93.5 |  |
| Allowed regions, % | 6.5 |  |

Values in parentheses are for the highest-resolution shell.

Rsym = Σ |*I*obs − *I*avg|/Σ *I*avg; *R*work = Σ|| *F*obs | − |*F*calc||/Σ *F*obs.

*R*free was calculated using 5% of data.
