# Supplementary material for: Structural and functional studies of rabbit SAMD9 reveal a distinct tRNase module that underlies the antiviral activity
Source: PLoS Pathog. 2025 Jul 31;21(7):e1013118. doi: 10.1371/journal.ppat.1013118 (PMC12331169; doi:10.1371/journal.ppat.1013118)
Supplement: S2 Fig — HEK 293T cells were transfected with mCherry-hSAMD9 (wild-type or the indicated mutations) and infected with vK1-C7-/GFP+. Representative flow cytometry plots from one of the biological replicates are shown, with percentage of cells in each quadrant indicated. Relative infection rates between hSAMD9-expressing and nontransfected cells are derived from the flow cytometry data. Each biological replicate and SD are shown in the bar graph. Statistics: one-way ANOVA compared to the WT (****P < 0.0001). (PDF) [file ppat.1013118.s002.pdf]

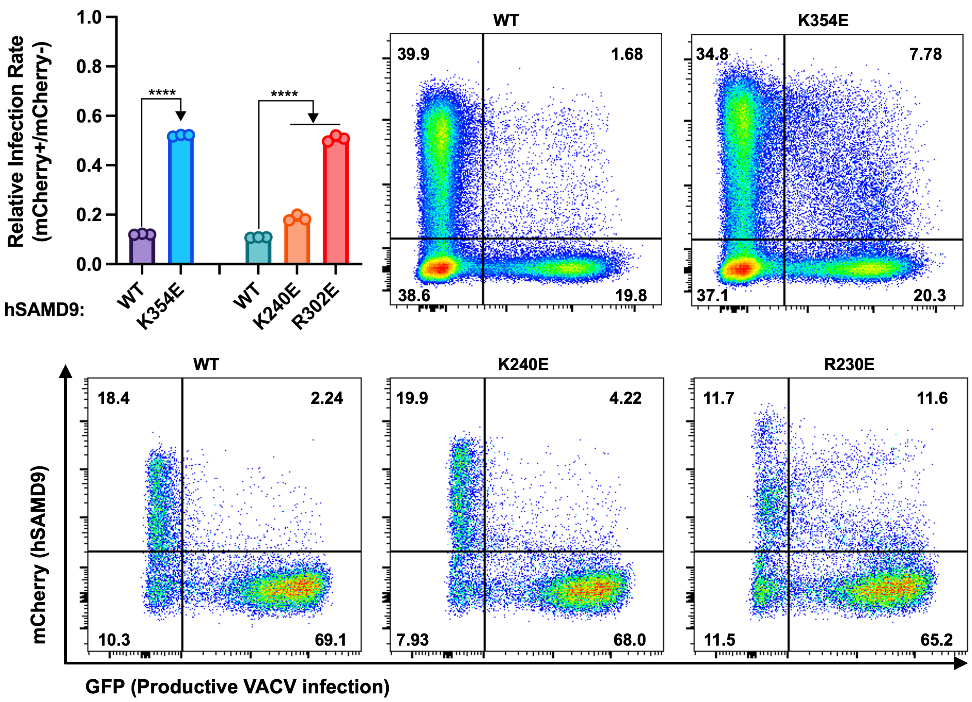

Relative Infection Rate (mCherry+/mCherry-)
1.0
0.8
0.6
0.4
0.2
0.0
****
****
hSAMD9:
WT
K354E
WT
K240E
R302E
WT
39.9
1.68
38.6
19.8
K354E
34.8
7.78
37.1
20.3
WT
18.4
2.24
10.3
69.1
K240E
19.9
4.22
7.93
68.0
R230E
11.7
11.6
11.5
65.2
mCherry (hSAMD9)
GFP (Productive VACV infection)

Figure S2
